# Natural active botanical metabolites: targeting AMPK signaling pathway to treat metabolic dysfunction-associated fatty liver disease

**DOI:** 10.3389/fphar.2025.1611400

**Published:** 2025-07-14

**Authors:** Hualing Wang, Xinyu Liu, Chunyi Wang, Shishuang Yu, Xiuli Yang, Xiyu Cao, Maocai Luo, Shiwei Liu, Chuantao Zhang

**Affiliations:** ^1^ Department of Respiratory Medicine, Hospital of Chengdu University of Traditional Chinese Medicine, Chengdu, China; ^2^ Wangjing Hospital, China Academy of Chinese Medical Sciences, Beijing, China

**Keywords:** metabolic dysfunction-associated fatty liver disease, AMPK, natural active botanical metabolites, lipid metabolism, *de novo* lipogenesis, fatty acid oxidation

## Abstract

Metabolic dysfunction-associated fatty liver disease (MAFLD), also known as non-alcoholic fatty liver disease (NAFLD), has emerged as one of the most common chronic liver diseases globally, with a tendency to progress gradually. With persistent disease progression, it may subsequently manifest as complications, including non-alcoholic steatohepatitis (NASH), cirrhosis, and liver cancer, and has been clinically established as a primary causative factor for liver failure and clinical scenarios necessitating liver transplantation. AMP-activated protein kinase (AMPK) is the central regulatory hub governing cellular energy homeostasis. It plays a central regulatory role in improving lipid metabolic disorders and represents a key molecular nexus for the management of MAFLD. Currently, the pathogenesis of MAFLD remains unclear, and treatment options are still limited, posing a significant public health challenge. Natural active botanical metabolites, which are important sources of novel therapeutic drugs, are widely available in nature and characterized by strong practicability and low cost. Growing evidence suggests that natural active botanical metabolites have definite therapeutic effects on MAFLD and hold broad application prospects. This study aims to systematically review *in vivo* and *in vitro* experimental evidence on natural active botanical metabolites targeting the AMPK pathway for the treatment of MAFLD. Based on our research findings, it is anticipated that effective natural active botanical metabolites can be incorporated into novel formulations in the future, which are expected to facilitate its bench-to-bedside transformation.

## Graphical Abstract

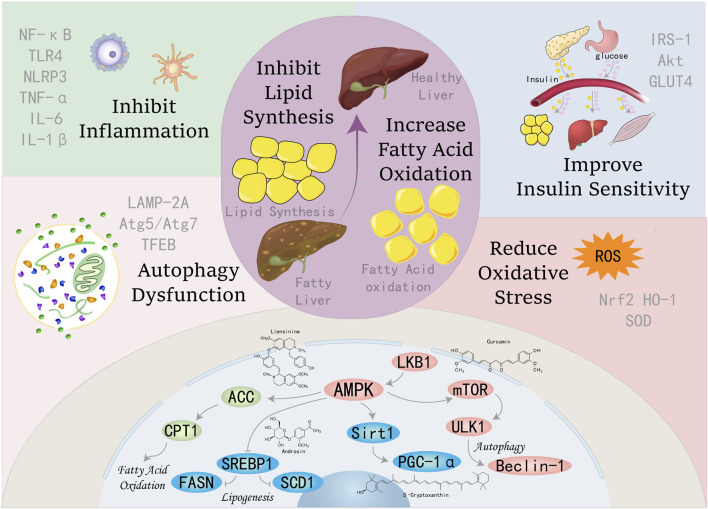

## 1 Introduction

The nomenclature for non-alcoholic fatty liver disease (NAFLD) has been updated to metabolic dysfunction-associated fatty liver disease (MAFLD), which is the latest terminology for fatty liver disease associated with metabolic syndrome (Chan et al., 2023). This change was first proposed by Eslam et al., in 2020 (Eslam et al., 2020a; Eslam et al., 2020b). The diagnostic criteria for MAFLD have been redefined as follows: based on imaging or histologically confirmed hepatic steatosis, patients must exhibit at least one concurrent metabolic abnormality: ① dysmetabolic weight status; ②confirmed type 2 diabetes mellitus (meeting WHO diagnostic criteria); ③metabolic dysfunction evidenced by two or more cardiometabolic risk factors (abdominal adiposity, atherogenic dyslipidemia, hypertension, or prediabetic states). It is worth noting that, unlike the diagnostic criteria for NAFLD, excluding other liver diseases (including alcoholic, autoimmune, or viral hepatitis) is not a prerequisite for diagnosing MAFLD. This updated definition was introduced to better reflect the metabolic nature of the disease to remove the “non-alcoholic” label, and place greater emphasis on clinical phenotypes (Eslam et al., 2020a). MAFLD is a high-prevalence hepatopathy that causes extensive liver damage. Histologically, it manifests as lobular inflammation and hepatocyte ballooning, among other features (Chan et al., 2023). In severe cases, it can lead to adverse hepatic outcomes such as liver fibrosis, cirrhosis, and hepatocellular carcinoma (Huang and Liu, 2023). Therefore, MAFLD is also considered a significant factor influencing the mortality rate of liver-related diseases. With the gradual prevalence of diabetes, and obesity globally, the overall prevalence of MAFLD has also shown an upward trend (Sangro et al., 2023; Younossi et al., 2023; Liu K. et al., 2024). MAFLD has emerged as a major public health issue, affecting up to 25% of the global adult population (Saiman et al., 2022). Studies estimate that from 1991 to 2019, the prevalence of MAFLD has increased from 21.9% to 37.3%, with a yearly increase of 0.7% (*P* < 0.0001), and it is still increasing, posing a significant economic, health, and social burden globally (Le et al., 2022). The current understanding of the pathogenesis of MAFLD remains unclear. Early researchers proposed the “two-hit” theory to explain the pathomechanism of MAFLD. This theory suggests that hepatic steatosis and insulin resistance (IR) caused by abnormal accumulation of free fatty acids constitute the “first hit”. The “second hit” phenomenon is characterized by secondary tissue injury, inflammatory responses, metabolic dysregulation including insulin resistance, fibrotic remodeling, and other pathological alterations, mediated through mechanisms involving elevated oxidative stress, peroxidative damage to lipids, and compromised mitochondrial bioenergetics (Basaranoglu et al., 2013; Friedman et al., 2018; Engin, 2024). Recently, it has been generally accepted that the more accurate pathogenesis of MAFLD is the “multiple-hit” hypothesis, which builds upon the “second-hit” theory. It indicates that various harmful factors team up to affect genetically prone individuals, causing MAFLD. These factors include oxidative stress, mitochondrial dysfunction, endoplasmic reticulum stress, gut microbiota changes, and lipotoxicity (Pafili and Roden, 2021; Guo et al., 2022).

Adenosine 5′-monophosphate (AMP)-activated protein kinase (AMPK) is a ubiquitously expressed serine/threonine protein kinase in the human body. AMPK consists of a heterotrimeric complex comprising a catalytic α subunit (α1, α2), a structurally and regulatory essential β subunit (β1, β2), and a regulatory γ subunit (γ1, γ2, γ3) (Cui et al., 2023). AMPK can sense the intracellular energy state and is activated when intracellular ATP concentration decreases and AMP or ADP concentration increases (Steinberg and Hardie, 2023). Activated AMPK initiates corresponding biological responses by phosphorylating a series of downstream target proteins to restore cellular energy homeostasis. The specific mechanisms include inhibiting *de novo* lipogenesis (DNL), promoting fatty acid oxidation (FAO) and lipid breakdown, as well as maintaining mitochondrial functional integrity to regulate autophagy and oxidative stress, among others (Smith et al., 2016; Fang et al., 2022). It maintains the steady state of lipid metabolism through the activation of its mediated signaling axes such as the LKB1-AMPK axis, SIRT1-AMPK axis, AMPK-ACC axis, AMPK-SREBP1 axis, and AMPK-mTOR axis. Numerous studies have shown that the AMPK pathway may be a promising target of action for the treatment of MAFLD (Fang et al., 2022).

Unfortunately, the complex pathophysiological characteristics of MAFLD make it difficult to find a single effective treatment method. Clinical options for treating MAFLD are limited, mainly through lifestyle changes such as dietary changes and increased exercise (Romero-Gómez, 2022). The development of pharmacological treatment regimens remains ongoing. Current drugs under clinical investigation include antidiabetic agents, Farnesoid X Receptor (FXR) agonists, Peroxisome Proliferator-Activated Receptor (PPAR) agonists, thyroid hormone receptor (THR) agonists, etc. However, these therapies face challenges such as toxicity/side effects, undefined optimal dosing regimens, unclear mechanisms of action, and insufficient validation through large-scale clinical trials (Sangro et al., 2023). As of 2024, Resmetirom is the only drug approved by the Food and Drug Administration (FDA). It is an oral, liver-targeted, thyroid hormone beta receptor-selective agonist. Although clinical trials have demonstrated resmetirom’s therapeutic potential for non-cirrhotic MASH and moderate to advanced liver fibrosis, gastrointestinal adverse events such as diarrhea and nausea remain significantly higher compared to the placebo group. The potential risks of diseases related to the thyroid, gonads, or bones also need to be monitored. Furthermore, its long-term safety and sustainability have not yet been confirmed through large-scale clinical trials (Bittla et al., 2024; Petta et al., 2024; Suvarna et al., 2024). Consequently, the clinical implementation of resmetirom remains a significant challenge. Compared to Western medicine, traditional Chinese medicine has garnered increasing attention from researchers due to its multi-target and multi-channel mechanisms of action. Natural active botanical metabolites refer to single chemical metabolites isolated, purified, and identified from plants, possessing a well-defined chemical structure (e.g., alkaloids, flavonoids, terpenoids) and exhibiting specific biological activities. They demonstrate a range of biological activities, such as anti-inflammatory and antioxidative effects (Zheng et al., 2023). As isolated bioactive metabolites, they are pivotal in deciphering the molecular mechanisms of traditional herbal therapies and represent a critical source for novel drug discovery. According to a previous study, it was found that many natural active botanical metabolites show great potential in treating MAFLD by inhibiting lipogenesis and promoting FAO, among other effects. Therefore, in this article, we review the research progress on natural active botanical metabolites targeting AMPK-related pathways for the treatment of MAFLD and summarize the associated mechanisms. It is anticipated that effective active metabolites can be incorporated into novel formulations in the future, further advancing the development of clinically effective drugs for MAFLD.

## 2 Mechanisms by natural active botanical metabolites target AMPK to ameliorate MAFLD

**FIGURE 1:**
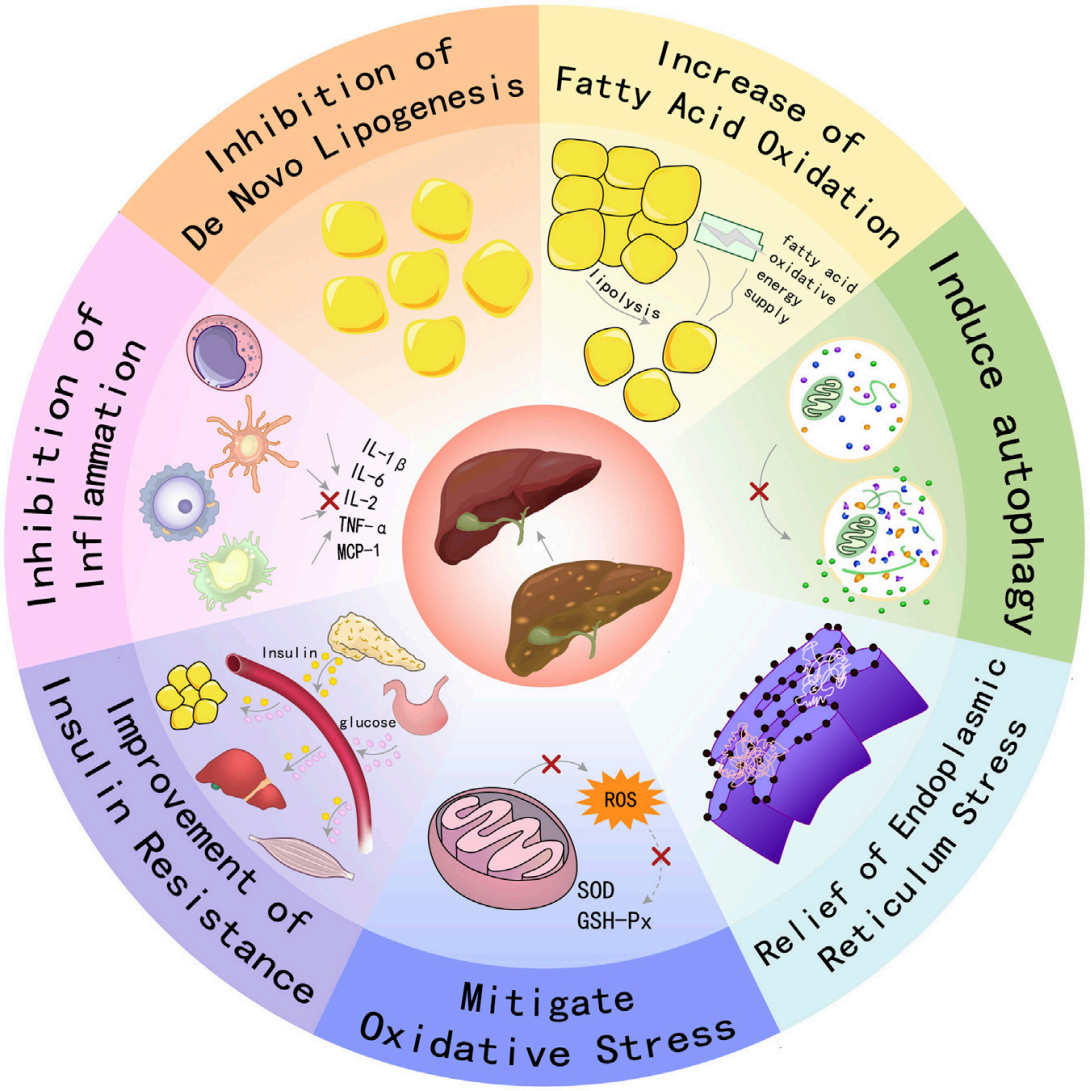
Schematic illustration showing partial mechanisms of activated AMPK against MAFLD: (1) Activating AMPK can inhibit key enzymes in the *de novo* lipogenesis pathway, reduce the synthesis and accumulation of fat in the liver, and thereby alleviate the severity of fatty liver; (2) Promoting FAO increases energy supply and reduces the accumulation of fatty acids in the liver, which helps improve hepatic steatosis; (3) Inducing the process of autophagy to clear damaged organelles and protein aggregates, reducing endoplasmic reticulum stress, thereby protecting hepatocytes from damage; (4) Enhancing the cellular antioxidant defense system, reducing the production of ROS, and up-regulating the activities of antioxidant enzymes such as SOD and GSH-Px to protect hepatocytes from oxidative damage; (5) Enhancing insulin signal transduction, increasing insulin sensitivity, promoting glucose uptake and utilization, reducing hepatic gluconeogenesis, thereby improving insulin resistance. (6) Inhibiting inflammatory signaling pathways, reducing the production of inflammatory factors (such as IL-1β, IL-6, TNF-α, etc.), alleviating inflammatory responses in the liver, and preventing the progression of MAFLD to more severe liver diseases.

AMPK functions as a pivotal regulator of metabolic homeostasis, employing a repertoire of mechanisms to suppress lipogenesis, enhance FAO, mitigate inflammation and oxidative stress, induce autophagy, and alleviate ER stress and insulin resistance. These pleiotropic effects position AMPK as a promising therapeutic target for the management of MAFLD and other metabolic disorders, see Figure 1.

### 2.1 Inhibition of *de novo* lipogenesis

*De novo* lipogenesis (DNL) is the core regulatory axis of energy metabolism homeostasis. The metabolic reprogramming process converts excess carbohydrate substrates (mainly glucose/fructose) into triglycerides (TG) and cholesterol through a series of chemical reactions, and then these TG and cholesterol lately provide energy through β-oxidation. The DNL pathway is a metabolic process primarily active in the liver (Cross et al., 2023). DNL encompasses a series of coordinated enzymatic reactions. The process initiates with ATP-citrate lyase (ACLY) catalyzing the conversion of citrate into acetyl-CoA. Subsequently, acetyl-CoA carboxylase (ACC) carboxylates the resulting acetyl-CoA to produce malonyl-CoA. Malonyl-CoA then enters the fatty acid synthesis pathway to synthesize fat. Notably, ACC exists in two distinct isoforms - ACC1 and ACC2, which are encoded by separate genes and perform divergent physiological functions (Smith et al., 2016). In detail, ACC1 is key in the DNL process, while ACC2 primarily oversees FAO (Wang et al., 2022b).

Studies have found that AMPK inhibits DNL through multiple pathways. Firstly, AMPK activation phosphorylates ACC, leading to a decrease or inactivation of ACC activity, thereby blocking the first step in fatty acid synthesis. Concurrently, activated AMPK also inhibits the nuclear translocation of SREBP-1c, phosphorylates the precursor of SREBP-1c, and prevents SREBP-1c from converting into its mature form, thus reducing abnormal lipogenesis. In addition, activated AMPK can also inhibit lipogenesis by enhancing the expression of SIRT1. SIRT1, a member of the Sirtuins family, is an NAD^+^-dependent class III histone deacetylase. AMPK enhances SIRT1 activity by increasing intracellular NAD^+^ levels (Cantó et al., 2009). Activated SIRT1 inhibits SREBP-1c activity in the liver, thereby reducing fat synthesis. For instance, treatment with resveratrol, a potent SIRT1 activator, has been demonstrated to significantly reduce acetylated SREBP-1c levels in obese mouse models (Ponugoti et al., 2010). Interestingly, SIRT1 also promotes the deacetylation of liver kinase B1 (LKB1), an upstream kinase of AMPK, to increase AMPK phosphorylation and activity, and ultimately, through the LKB1-AMPK-SIRT1 signaling axis can attenuate MAFLD through the modulation of DNL process (Lan et al., 2008).

### 2.2 Increase of fatty acid oxidation

Fatty acid oxidation (FAO) refers to the process by which fatty acids are broken down into CO_2_ and H_2_O in the presence of oxygen, releasing a significant amount of energy, primarily occurring in mitochondria (Lu and George, 2024). Dysregulation of FAO creates an imbalance between lipid acquisition and processing, resulting in abnormal deposition of lipid droplets in hepatocytes, a pathologic process that is a central driving mechanism of MAFLD (Ipsen et al., 2018). Carnitine palmitoyltransferase 1 (CPT1) is a crucial rate-limiting enzyme in FAO (Schlaepfer and Joshi, 2020), catalyzing the complete oxidation of fatty acids through a series of biochemical reactions. Malonyl-CoA functions as an allosteric inhibitor of CPT1. When AMPK is activated, it phosphorylates and inactivates ACC, which in turn decreases the synthesis of malonyl-CoA. Thereby increasing CPT1 expression and promoting FAO (Tian et al., 2019). Activation of AMPK significantly increases the activity of lipolytic enzymes such as adipose triglyceride lipase (ATGL) and hormone-sensitive lipase (HSL), thus promoting lipolysis, increasing the concentration of fatty acids in the cell, and providing substrates for the subsequent oxidation process. The main site of FAO is in the mitochondria, and the activation of AMPK can also regulate the function of mitochondria, which is also conducive to the promotion of FAO. Therefore, multiple regulatory mechanisms interact to form positive feedback, driving the metabolic process of FAO while ensuring precise maintenance of energy homeostasis at the cellular and body levels.

### 2.3 Inhibition of inflammation

The inflammatory reaction is closely associated with the body’s metabolism and often influences each other. For example, obesity and type 2 diabetes, among others, trigger an increase in inflammatory markers in the liver, adipose tissue, and skeletal muscle, while immune dysfunction exacerbates various metabolic disorders (Saltiel and Olefsky, 2017; Fan et al., 2022). Inflammation also plays an important role in MAFLD, and the immune system plays an integral role in the gradual evolution of MAFLD from simple steatosis to NASH and then to the more advanced NASH-associated fibrosis (Friedman et al., 2018; Peiseler et al., 2022). Activated AMPK achieves negative regulation of inflammatory response by inhibiting the expression of pro-inflammatory cytokines (TNF - α, IL-6) and NF - κ B signaling transduction, accompanied by compensatory upregulation of anti-inflammatory proteins. For instance, the activation of AMPK can enhance the activity of SIRT1, which modifies histones and other proteins through deacetylation to suppress the expression of inflammatory genes (Xu C. et al., 2021). AMPK also interacts with other signaling pathways to jointly regulate inflammatory responses. For example, there is an intimate contact between AMPK and the PI3K/Akt/mTOR signaling pathway.

### 2.4 Induction of autophagy

Autophagy is a lysosome-mediated self-degradative process that eliminates misfolded proteins, aggregated macromolecules, and damaged organelles (e.g., mitochondria and endoplasmic reticulum). By clearing these metabolites, autophagy mitigates oxidative stress and inflammation, thereby maintaining cellular homeostasis. Autophagy is generally regarded as a survival mechanism that breaks down cellular debris to supply recycled metabolites and energy, thereby supporting cellular renewal and homeostasis (Hotamisligil, 2006; Liang L. et al., 2022). The regulatory function of autophagy cannot be overlooked in the pathophysiological progression of multiple disorders, including but not limited to neoplastic transformations, metabolic dysregulations, immune-mediated pathologies, and pathogen-associated conditions. Lipophagy, a subtype of autophagy, can target intracellular lipid droplets and degrade them to regulate fat storage in the liver, and impaired autophagic flux is closely associated with the development of MAFLD (Czaja, 2016; Niture et al., 2021). As an evolutionarily conserved serine/threonine kinase, the mammalian target of rapamycin (mTOR) serves as a central regulatory hub by integrating nutrient signals to coordinate metabolic reprogramming, translational regulation, and programmed cell death, while also dynamically regulating cellular growth and proliferation. Functioning as a critical modulator of cellular energetics, mTOR enhances metabolism and inhibits autophagy (Xie et al., 2023; Liu X. et al., 2024). mTOR is a key effector in AMPK signaling cascades, and it oppositely regulates autophagy induction with crosstalk in modulating energy balance (Xu et al., 2012). Specifically, mTORC complex 1 (mTORC1) downregulates the autophagy process, whereas AMPK has a direct or indirect positive impact on autophagy induction (Alers et al., 2012). Therefore, the AMPK-mTOR axis plays a crucial role in regulating lipid metabolism. To date, numerous scientific studies have confirmed this notion. Silibinin promotes autophagy in high fructose-exposed HepG2 cells through AMPK activation, mTOR inhibition, and downregulation of autophagy suppressors LC3/beclin-1 (Li et al., 2019). AMPK also alleviates hepatic steatosis by activating autophagy through AMPK-mediated PGC-1α/PPARα pathways (Yang et al., 2024).

### 2.5 Mitigation of oxidative stress

The fundamental biological nature of oxidative stress (OS) lies in the dynamic imbalance between the intracellular production rate of reactive oxygen species (ROS) and the antioxidant defense capacity, resulting in the accumulation of oxidative damage (Hajam et al., 2022; Sadasivam et al., 2022). Excessive ROS generation depletes endogenous antioxidants, rendering the body unable to neutralize the surplus ROS, thereby leading to cellular damage. OS serves as a pivotal pathogenic driver in MAFLD progression, inducing hepatic inflammatory cascades, compromising mitochondrial bioenergetics, and triggering extracellular matrix deposition within hepatocytes. More severely, excessive OS leading to progressive hepatocyte death can facilitate the development of liver cirrhosis or hepatocellular carcinoma (HCC) (Takaki et al., 2013; Spahis et al., 2017; Clare et al., 2022). Studies have confirmed that berbamine enhances the antioxidant defense system by activating the antioxidant transcription factor nucleus factor erythroid 2-related factor 2 (Nrf2), upregulating the expression of HO-1, Nqo-1, SOD2, and catalase. This effect is primarily achieved through the activation of AMPK, which stimulates the Nrf2/ARE signaling pathway (Sharma et al., 2020). AMPK also inhibits oxidative stress in liver tissue by stimulating SIRT1 expression, which increases GSH-Px, GSH, SOD, and CAT content and significantly decreases MDA levels (Chen et al., 2024).

### 2.6 Other mechanisms of action

In addition to the major mechanisms mentioned above, endoplasmic reticulum stress (ER stress) is also one of the key pathological mechanisms. As a cellular protective stress response, ER stress functions to alleviate the disruption of cellular homeostasis caused by protein misfolding, thereby triggering the unfolded protein response (UPR) (Lebeaupin et al., 2018). ER stress can initiate and accelerate the progression of diverse liver ailments, including metabolic-associated fatty liver disease (MAFLD), viral hepatitis, liver injury caused by medications, as well as hepatocellular carcinoma. It has been found that AMPK activation reduces ER stress markers Bip, ATF, and CHOP as well as phosphorylated ERK/JNK, thereby alleviating ER stress. While AMPK-stimulated autophagy is also directly related to the reduction of ER stress (Seo et al., 2024). In addition, insulin resistance is a central driver of MAFLD, promoting disease progression through systemic metabolic disturbances (adipose tissue, skeletal muscle, intestinal tract) and local intrahepatic signaling dysregulation (selective resistance, ER stress) (Sakurai et al., 2021). Activated AMPK improves metabolic status by regulating signaling pathways and metabolic pathways associated with insulin resistance to enhance insulin sensitivity to treat MAFLD (Fouqueray et al., 2021).

To determine the mechanisms by which natural active botanical metabolites ameliorate MAFLD through targeting the AMPK signaling pathway, Non-alcoholic Fatty Liver Disease, Metabolic dysfunction-associated fatty liver disease, NAFLD, MAFLD, AMP-Activated Protein Kinases, AMPK, Chinese herbs, Chinese medicine, Herbal medicine, Plant medicine, Natural medicine, Botanical drug, Phytomedicine were used as keywords or subject headings to search for relevant articles in the Web of Science and PubMed databases from 2019 to 2024. After identifying 1,111 potentially relevant articles, the screening protocol sequentially discarded 342 duplicate records and disqualified 85 review papers, ultimately retaining 684 articles for in-depth analysis. Following initial screening, publications were systematically filtered to eliminate entries conforming to pre-defined exclusion criteria: (1) non-medical articles; (2) articles that study combinations composed of various botanical drugs (e.g., decoctions); (3) articles where the study target was not natural active botanical metabolites; (4) articles classified as commentaries; (5) articles on natural active botanical metabolites unrelated to the AMPK pathway; (6) articles with severely missing experimental data; (7) articles lacking animal experiments; and (8) articles with unavailable full texts. Finally, 120 articles were included, involving 101 natural active botanical metabolites, and summarized information on plant sources, classifications, and other details were summarized (Supplementary Table 1).

## 3 Natural active botanical metabolites improving MAFLD by targeting AMPK

### 3.1 Alkaloids

**FIGURE 2:**
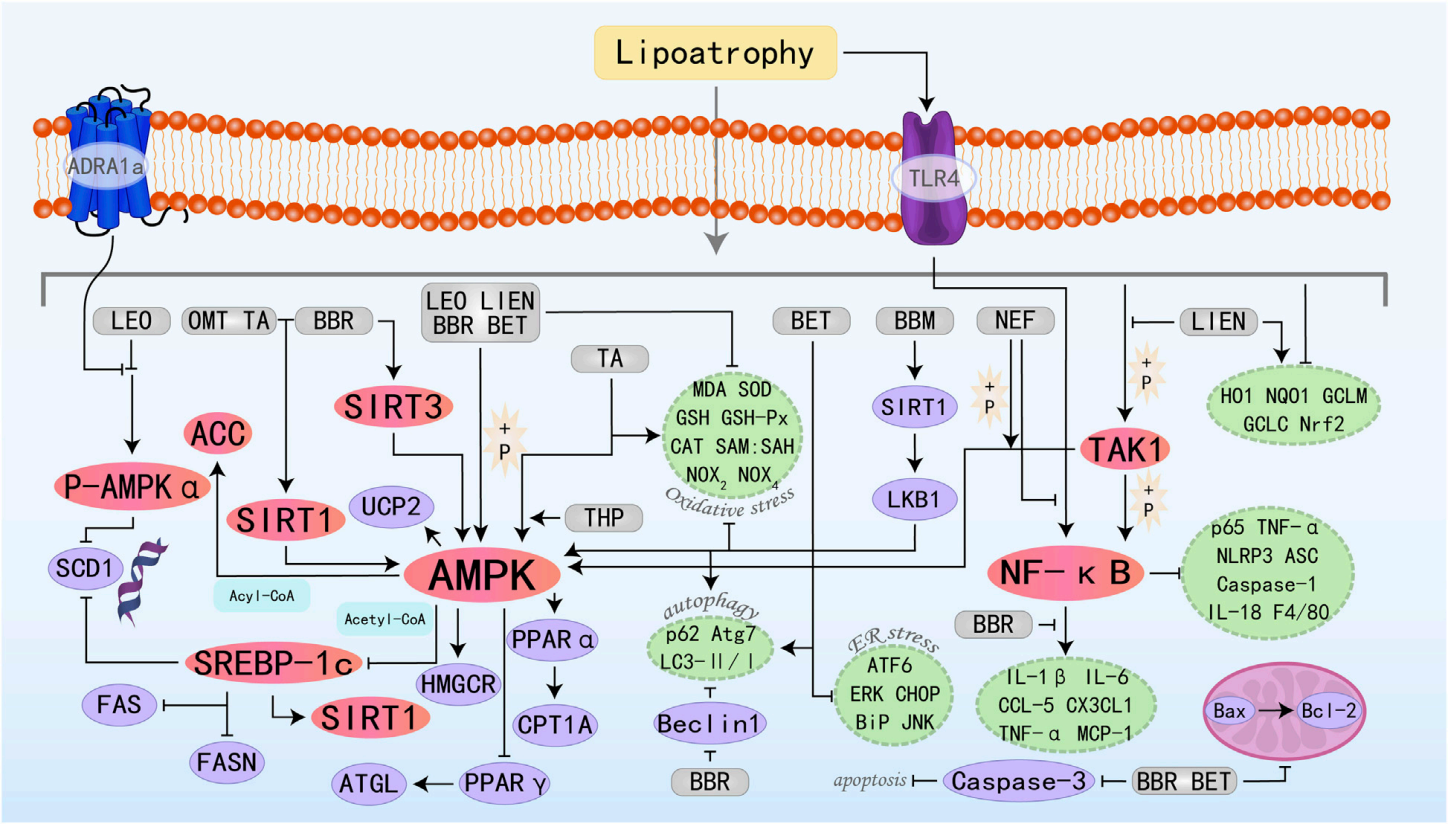
Molecular mechanism of alkaloid metabolites from botanical drugs targeting AMPK to treat MAFLD. LEO, Leonurine; LIEN, Liensinine; BBR, Berberine; OMT, Oxymatrine; BET, Betaine; BBM, Berbamine; TA, Tomatidine; NEF, Neferine; THP, Tetrahydropalmatine.

Plant alkaloids are metabolites with complex structures found in natural plants. Due to the different arrangements and combinations of functional groups, various alkaloids can be formed, possessing multifaceted pharmacological profiles, such as anticancer, inflammatory cascade attenuation, pathogen eradication, oxidative damage mitigation, hypertensive state amelioration, and immune homeostasis maintenance (Bhambhani et al., 2021). Therefore, they are regarded as a potentially important source of drugs for the treatment of related diseases in the future. Many alkaloid metabolites have demonstrated positive protective effects against MAFLD. The specific molecular pathways by which nine alkaloid plant metabolites target the AMPK pathway to effectively suppress the onset and advancement of MAFLD are illustrated in Figure 2.

Leonurine (LEO) is a natural alkaloid extract derived from *Herba leonuri*. Studies have shown that LEO exhibits anti-MAFLD effects both *in vivo* and *in vitro* experiments. It regulates lipid homeostasis through modulation of the AMPK/SREBP1 signaling axis, suppressing hepatic fat accumulation while concurrently attenuating oxidative damage and inflammatory responses in liver parenchyma (Zhang et al., 2019a). LEO exerts its regulatory function by upregulating ADRA1a expression—a G protein-coupled receptor—which sequentially induces phosphorylation and activates the downstream AMPK signaling cascade. This regulates the expression of its downstream protein stearoyl-CoA desaturase 1 (SCD1), reducing fatty acid levels (Fan et al., 2024). Liensinine (LIEN) is also a plant-derived isoquinoline alkaloid with various pharmacological effects, including anti-inflammatory, antioxidant, anti-apoptotic, and autophagy-modulating properties (Zhang W. et al., 2023). *In vitro* analyses reveal that LIEN can stimulate the AMPK/ACC signaling axis, which in turn rescues the expression of key FAO-regulatory factors including PPARα, CPT-1α, and uncoupling protein 2 (UCP2). This hierarchical modulation ultimately enhances FAO capacity. Furthermore, it suppresses oxidative stress and inflammation via activation of the transforming growth factor-β-activated kinase 1 (TAK1)-dependent AMPK pathway, culminating in MAFLD improvement (Liang L. et al., 2022). Berberine (BBR) is the main active component in *Coptis chinensis Franch.* In traditional medicine and is regarded as one of the most promising natural product-derived drugs for the treatment of cardiovascular and metabolic diseases (Feng et al., 2019). *In vivo* studies have shown that BBR can inhibit lipogenesis and promote FAO by activating the SIRT3/AMPK/ACC pathway in the liver, thereby improving hepatic steatosis (Zhang et al., 2019a). Further studies have also demonstrated that BBR inhibits the transcriptional activity of the SRE motif (a potential SREBP-1c binding site) within the SCD1 promoter through the AMPK-SREBP-1c pathway, thereby reducing hepatic lipogenesis (Zhu et al., 2019). BBR can also stimulate AMPK/SIRT1 signaling to regulate downstream effector molecules controlling lipid synthesis, transport, and catabolism—specifically SREBP-1c and PPARα. Additionally, it modulates FOXO transcription factors, NF-κB, Bcl-2/Bax, and cleaved caspase 3, thereby controlling oxidative stress, inflammation, and apoptosis, ultimately achieving the goal of treating MAFLD (Chen et al., 2024). Oxymatrine (OMT) belongs to the class of matrine-type alkaloids, which are primarily isolated from *Sophora flavescens Aiton*. Studies have shown that OMT, similar to BBR, primarily regulates hepatic lipid metabolism by activating the SIRT1/AMPK pathway and modulating downstream relevant molecules (Xu et al., 2020). Betaine (BET), a compound also termed trimethylglycine, occurs naturally in various life forms such as animals, plants, and microorganisms. It is a non-essential amino acid that plays a crucial role in metabolic diseases through various effects such as exhibiting anti-inflammatory properties, restoring mitochondrial function, and improving insulin resistance (Chen et al., 2022b). Fibroblast growth factor 10 (FGF10), a key component of the FGF family, is indispensable for the proper development of multiple organs. A previous study suggested that the miR-327-FGF10-FGFR2 signaling axis represents a therapeutic target for the treatment of obesity and metabolic diseases (Fischer et al., 2017). Building upon this foundation, Chen W et al. conducted research and confirmed that BET treatment induces FGF10 secretion, which stimulates AMPK activation and inhibits lipogenesis, increases FAO, and ultimately reduces hepatic lipid accumulation, identifying a potential new therapeutic target for MAFLD (Chen et al., 2021). Additionally, studies have found that BET can also activate autophagy (LC3II/I and p62), thereby reducing downstream signals such as ER stress (BiP, ATF6, and CHOP), apoptosis (Bax, cleaved caspase 3), and steatosis (Seo et al., 2024). Berbamine (BBM) is a dibenzylisoquinoline alkaloid extracted from the Chinese herbal medicine genus Berberis, which possesses anti-inflammatory, leukocytosis-inducing, and anti-tumor effects. For decades, it has maintained its clinical relevance in addressing a broad spectrum of pathological conditions. Among these studies, Sharma A et al. found that BBM activates SIRT1-mediated LKB1 deacetylation, upregulates AMPK signaling, and ultimately regulates the expression of downstream lipid metabolism factors, improves mitochondrial physiological structure and function, restores autophagy, and alleviates oxidative stress, playing a therapeutic role in MAFLD (Sharma et al., 2021). Tomatidine (TA) is a natural steroidal alkaloid and is the aglycone of α-tomatine (αTM). Previous studies have found that TA can act as a vitamin D agonist, activating AMPK through calmodulin-dependent protein kinase β (CaMKKβ) in response to increased intracellular Ca2+ concentrations, inhibiting lipid accumulation (Kusu et al., 2019). Neferine (NEF) exerts its therapeutic potential in MAFLD management by stimulating AMPK-dependent cellular energy reprogramming, demonstrating efficacy *in vitro* and *in vivo*. Additionally, it prevents MAFLD-related fibrosis by inhibiting the TGFβ-Smad2/3 pathway (Wang et al., 2023). Tetrahydropalmatine (THP) is known for its anti-inflammatory and analgesic effects with promising applications and potential development value (Chen X. et al., 2023). Yin X et al. have demonstrated that the AMPKα protein is a direct target of THP, which can promote FAO, improve lipid metabolism, and alleviate lipotoxicity through the AMPK/SREBP-1c/SIRT1 pathway (Yin et al., 2023).

### 3.2 Flavonoids

**FIGURE 3:**
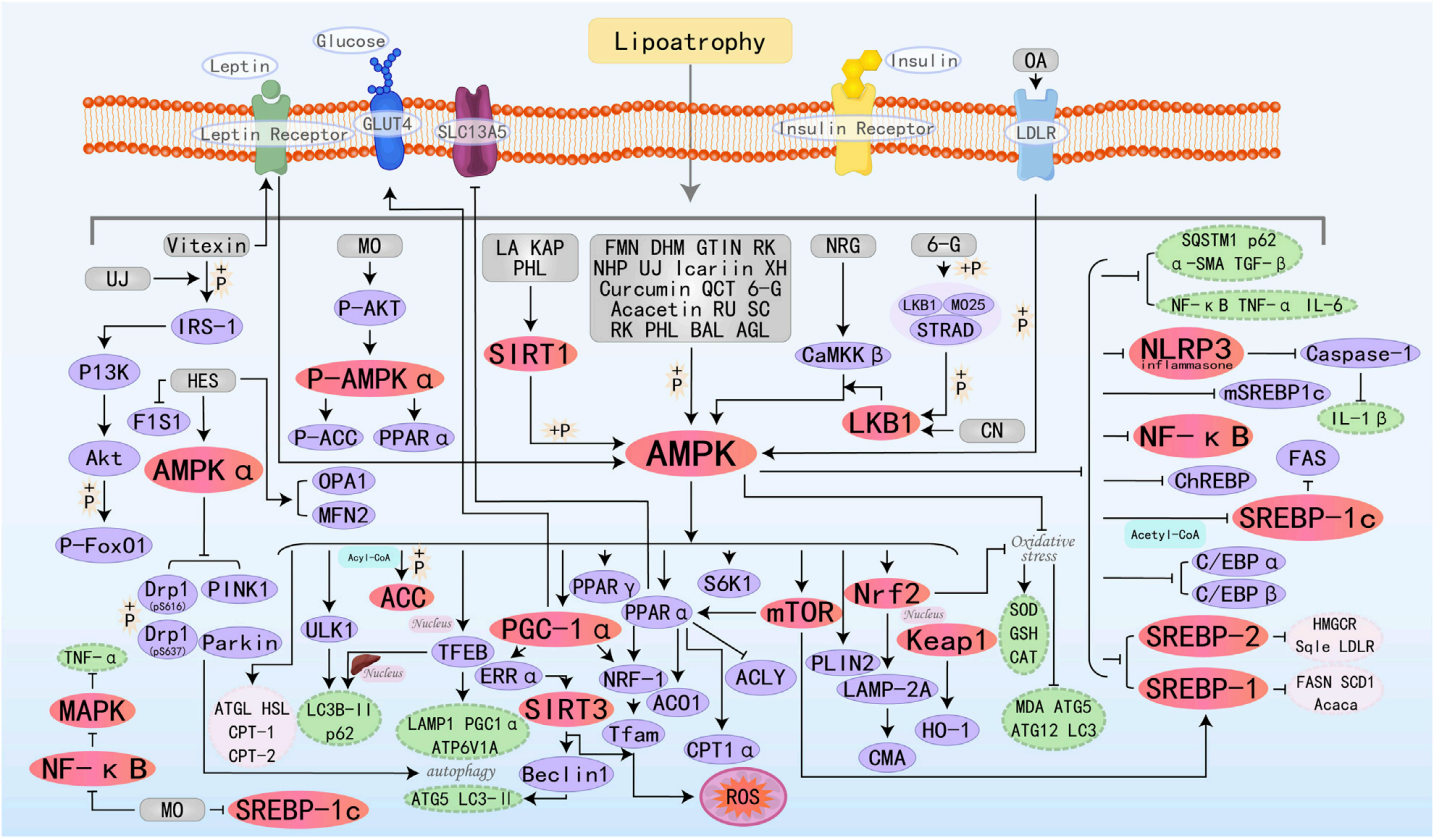
Molecular mechanism of flavonoid metabolites from botanical drugs targeting AMPK to treat MAFLD. LA, Licochalcone A; FMN, Formononetin; MO, Morin; DHM, Dihydromyricetin; BAL, Baicalein; NHP, Neohesperidin; UJ, Ugonin J; NRG, Naringenin; XH, Xanthohumol; QCT, Quercetin; CN, Chrysin; BA, Baicalin; KAP, Kaempferol; 6-G, 6-Gingerol; GTIN, Gossypetin; OA, Oroxin A; RU, Rutin; AGL, Apigenin; HES, Hesperitin; SC, Scopoletin; RK, Raspberry ketone; PHL, Phloretin.

Flavonoids represent a class of bioactive phytochemicals renowned for their pleiotropic health-promoting activities, with widespread occurrence in edible horticultural produce and medicinal botany. Flavonoids are characterized by a bipartite aromatic system comprising A and B benzene rings, which are conjugated through a central three-carbon linker, establishing the fundamental C6-C3-C6 carbon framework. Both synthetically produced and naturally isolated flavonoid metabolites exhibit a variety of biological activities, such as anti-tumor, antiplatelet, antimalarial, anti-inflammatory, and antidepressant effects (Guan and Liu, 2016). It exhibits therapeutic efficacy against cancer, inflammatory disorders (particularly cardiovascular and neurological diseases), and related pathologies (Rauf et al., 2023). Notably, flavonoids also demonstrate a potent role in the prevention of MAFLD. The specific molecular mechanisms by which 26 flavonoid active metabolites target the AMPK pathway to improve MAFLD are illustrated in Figure 3.

Vitexin is a natural flavonoid glycoside compound. Research has found that Vitexin activates AMPK by promoting the binding of leptin receptor (LepR) to AMPK, thereby inhibiting lipogenesis and promoting lipolysis and FAO. Additionally, Vitexin improves insulin signaling by activating insulin receptor substrate-1 (IRS-1) and its downstream target protein kinase B (AKT), ultimately leading to an improvement in MAFLD (Inamdar et al., 2019b). Formononetin (FMN) can promote the nuclear translocation of TFEB, a central regulator of the autophagy/lysosome-nuclear signaling pathway, by activating AMPK, thereby activating autophagy (upregulating LAMP1, ATP6V1A, and PGC1α, as well as promoting hepatic nuclear translocation of LC3B-II and p62) to improve autophagosome-lysosome fusion. Additionally, it can also promote FAO by increasing the expression of PPARα and CPT1α(Wang et al., 2019). Dihydromyricetin (DHM) promotes autophagy by activating AMPK/PGC-1α and PPARα, which leads to an increase in the levels of Beclin 1, ATG 5, and LC3-II (Yang et al., 2024). Furthermore, it can also prevent MAFLD by enhancing mitochondrial function through a SIRT3-dependent mechanism (Zeng et al., 2019). Neohesperidin (NHP) is a flavonoid glycoside extracted from citrus peel and utilized as a natural antioxidant. Studies have shown that NHP activates PGC-1α to mediate mitochondrial biogenesis and upregulates nuclear respiratory factor-1(NRF-1) and mitochondrial transcription factor A (TFAM) to enhance mitochondrial capacity and FAO (Wang S. W. et al., 2020). Ugonin J (UJ) is a flavonoid derived from *Helminthostachys zeylanica*, which exhibits anti-inflammatory and anti-osteoporotic effects (Huang et al., 2017). Chang TC et al. showed that UJ regulates lipid metabolism through the activation of factors related to downstream AMPK regulation, increases insulin secretion, improves insulin resistance, and regulates and lipid metabolism disorders by decreasing the ratio of pIRS-1 (Ser307))/IRS-1, and upregulates Akt activity and FoxO1 phosphorylation for the treatment of MAFLD (Chang et al., 2021). Icariin enhances the biosynthesis and membrane redistribution of GLUT4, the transporter protein responsible for glucose uptake, by activating the AMPK/PGC1α pathway. This process enhances glucose uptake and metabolism, while decreasing insulin resistance, ultimately contributing to the improvement of MAFLD (Lin et al., 2021). Curcumin is an active component derived from *Curcuma longa Linn.* With various biological activities including anti-inflammatory, antioxidant, and anticancer properties. It can be used in the treatment of cancer, and metabolic diseases, and as a neuroprotective agent (Mhillaj et al., 2019; Tomeh et al., 2019; Marton et al., 2021; Sadeghi et al., 2023). SLC13A5 is a citrate-selective transmembrane protein in hepatic citrate homeostasis and serves as an alternative energy source for metabolism. ACLY serves as a critical metabolic checkpoint coupling cytosolic glucose-derived carbon flux to DNL via acetyl-CoA availability. Sun QS et al. found that Curcumin may correct the deregulated expression of SLC13A5/ACLY by activating the AMPK-mTOR signaling pathway, thereby inhibiting DNL and reducing hepatic lipid accumulation (Sun et al., 2021). Chrysin (CN) possesses hepatoprotective potential due to its anti-inflammatory properties. Specific investigations have found that CN can activate the LKB1/AMPK/mTOR/SREBP-1c pathway, thereby inhibiting lipogenesis pathways. Additionally, CN also regulates the increased expression of genes involved in mitochondrial biogenesis, such as PGC-1α, NRF-1, and Tfam (Oriquat et al., 2023). The protective effects of Baicalin (BA) on MAFLD rely on AMPK-mediated downstream pathways. Specifically, inhibiting the AMPK/SREBP1 pathway and the AMPK/NF-κB pathway reduces fat synthesis and inflammatory responses, while activating the AMPK/Nrf2 pathway alleviates oxidative stress (Gao et al., 2023b). Kaempferol (KAP) primarily promotes the SIRT1/AMPK pathway, which leads to reduced hepatic lipogenesis and enhanced FAO (Li et al., 2023). 6-Gingerol (6-G) is one of the most biologically potent metabolites in *Zingiber officinale*, exhibiting anti-inflammatory, antioxidant, and anticancer pharmacological activities. STE20-related adapter (STRAD) and MO25 can form a complex with AMPK, promoting the cytosolic distribution and kinase activity of LKB1. Studies have shown that 6-G can induce the activation of the LKB1/AMPK pathway cascade by regulating the stability of the LKB1/STRAD/MO25 complex and activating LKB1, ultimately alleviating MAFLD (Liu Y. et al., 2023). Apigenin (AGL) is an edible flavonoid derived from plants, possessing various biological activities with significant potential applications in cancer and dermatological conditions (Imran et al., 2020; Yoon et al., 2023). Current research has also highlighted its value in treating MAFLD. Previous studies have demonstrated that phosphorylation of perilipin 2 (PLIN2) is a prerequisite for participating in chaperone-mediated autophagy (CMA), while LAMP-2A serves as a rate-limiting enzyme in the degradation of CMA-regulated substrate proteins. In a recent study by Lu J et al., it was found that AGL enhances CMA activity by activating AMPK, which promotes PLIN2 phosphorylation and Nrf2 nuclear translocation, and upregulates LAMP-2A protein. These effects ultimately facilitate lipid droplet degradation and improve MAFLD (Lu et al., 2024). Hesperitin (HES) alleviates MAFLD by reducing the expression of dynamin-related protein 1 (Drp1) through an AMPKα-dependent mechanism, phosphorylating Drp1 at serine 616 (Drp1-pS616), inducing phosphorylated Drp1 at serine 637 (Drp1-pS637), and enhancing mitochondrial autophagy through the induction of PTEN-induced kinase 1 (PINK1) and E3 ubiquitin protein ligase Parkin (Parkin) (Chen et al., 2025a). Many other types of flavonoids can improve hepatic lipid homeostasis by stimulating AMPK and regulating upstream and downstream molecular targets.

### 3.3 Lignans

**FIGURE 4:**
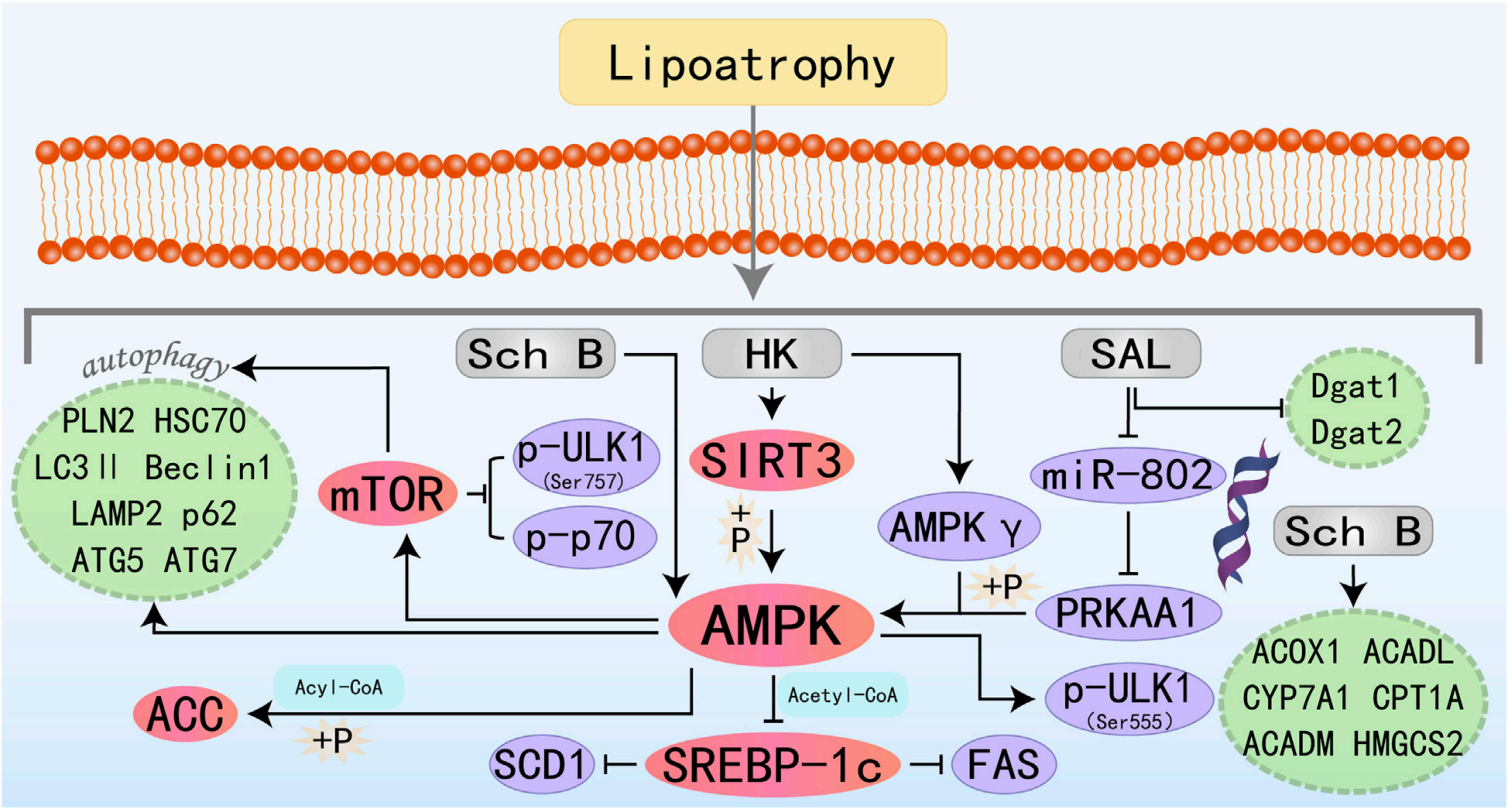
Molecular mechanism of lignans metabolites from botanical drugs targeting AMPK to treat MAFLD. HK, Honokiol; Sch B, Schisandrin B; SAL, Schisanhenol.

Lignans are secondary metabolites with phytoestrogenic physiological activity that are widely present in plants and human food sources, and have a variety of biological activities, including antibacterial, antiviral, antitumor, antiplatelet, antioxidant, and immunosuppressive activities (Fang and Hu, 2018). Lignans have also been found to have a promising role in the prevention and treatment of MAFLD. The specific molecular mechanisms by which three lignan phytomonomers target the AMPK pathway to improve MAFLD are illustrated in Figure 4.

Honokiol (HK) is an active substance isolated from Magnoliaceae species, existing as a fine brown to white powder. It demonstrates antibacterial, anti-inflammatory, oxidative stress-reducing, and cancer-inhibiting activities (Rauf et al., 2018). Emerging evidence suggests that HK exerts hepatoprotective effects by modulating lipid metabolism and mitigating oxidative stress associated with hepatic steatosis. Liu J et al. demonstrated that HK confers hepatoprotection against lipotoxic injury by enhancing SIRT3-AMPK-mediated autophagic flux and preserving mitochondrial ultrastructure integrity. These findings establish HK as a promising pharmacological candidate for MAFLD management (Liu et al., 2021b). Interestingly, another study has also found that HK-mediated activation of the AMPK complex does not depend on its classic upstream regulators, but rather directly binds to the AMPKγ1 subunit to act as an agonist of the AMPK complex, thereby modulating downstream molecules and ameliorating hepatic lipid accumulation (Tian et al., 2023). Schisandrin B (Sch B) is one of the most promising bioactive metabolites isolated from *Schisandra chinensis (Turcz.)*. Sch B has a wide range of promising applications in liver diseases and can ameliorate acute liver injury and MAFLD by activating autophagy, anti-inflammation, and direct regulation of adipocyte metabolism (Kwan et al., 2017; Ma et al., 2022; Li X. et al., 2023). The specific molecular mechanism of Sch B treatment of MAFLD may be related to the activation of the autophagy-lysosomal pathway by the AMPK/mTOR signaling axis and the promotion of FAO (Yan et al., 2022). Schisanhenol (SAL) is another lignan with antioxidant and anti-apoptotic properties (Han et al., 2019; Zhang et al., 2025b). MicroRNAs are short non-coding RNA molecules that regulate various biological pathways. Abnormal expression of mRNAs is strongly associated with disorders of glucose-lipid metabolism and contributes to many metabolic diseases, including obesity and MAFLD (Rottiers and Näär, 2012; Agbu and Carthew, 2021; Sun and Kemper, 2023). Research has demonstrated that SAL may activate the AMPK signaling pathway by inhibiting miR-802-mediated PRKAA1 repression, showing a promising therapeutic intervention for MAFLD (Li et al., 2024).

### 3.4 Phenolic acids

**FIGURE 5:**
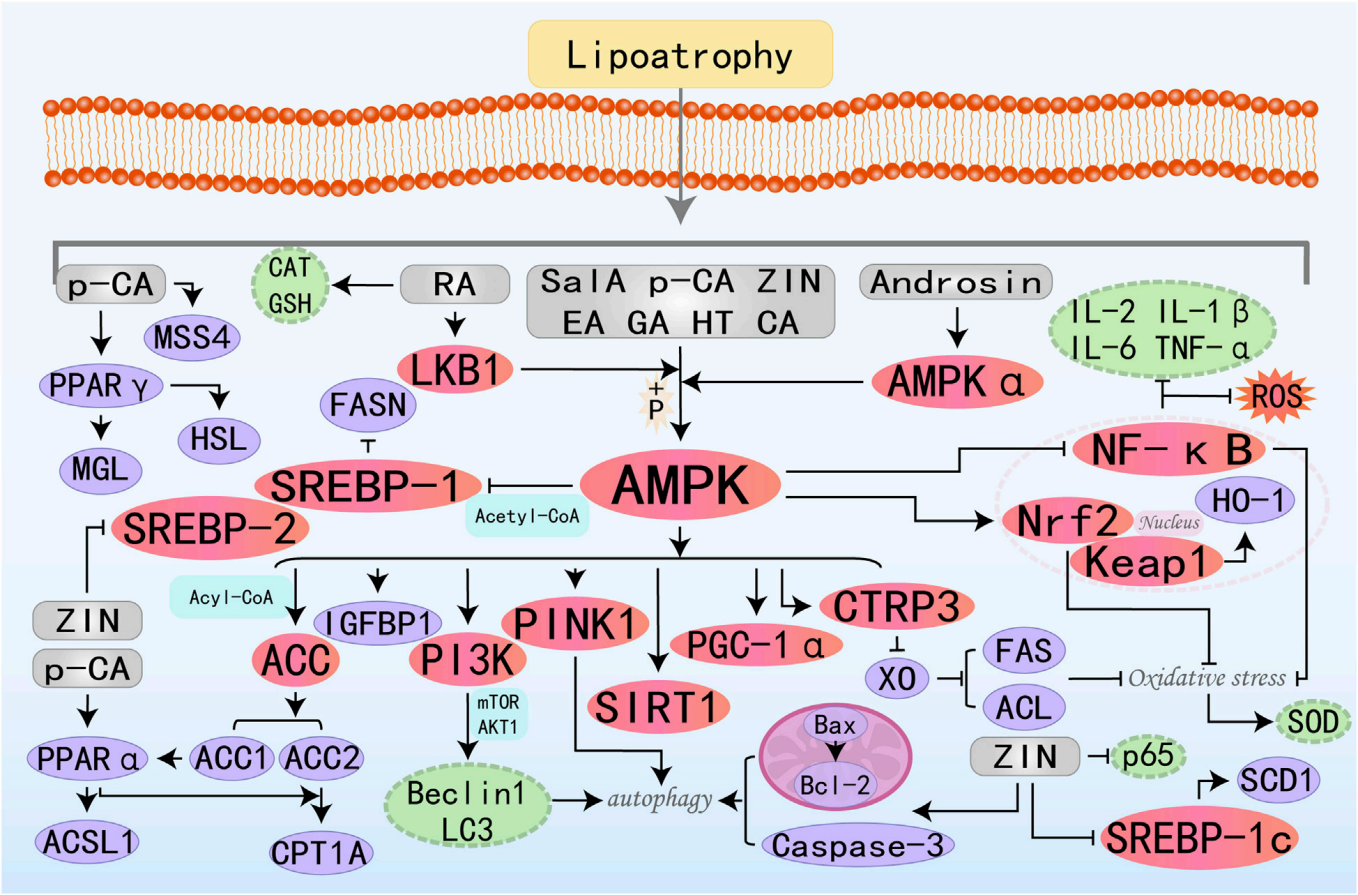
Molecular mechanism of phenolic acid metabolites from botanical drugs targeting AMPK to treat MAFLD. Sal A, Salvianolic acid A; HT, Hydroxytyrosol; EA, Ellagic acid; ZIN, Zingerone; GA, Gallic acid; *p*-CA, *p*-Coumaric Acid; RA, Rosmarinic Acid; CA, Chicoric Acid.

Phenolic acids represent a subclass of plant-derived metabolites defined by their aromatic systems bearing multiple hydroxyl substituents on a shared benzene ring, with C1-C6 and C3-C6 carbon frameworks constituting their primary structural variants. They are abundant in the seeds and peels of fruits as well as in the leaves of vegetables. Phenolic acids are extensively utilized in pharmaceutical formulations, dermatological products, and other fields, offering benefits such as oxidative stress mitigation, inflammation modulation, and carcinogenesis inhibition effects. Research findings indicate various phenolic acids can improve MAFLD. The specific molecular mechanisms by which nine phenolic acids treat MAFLD through the AMPK pathway are illustrated in Figure 5.

Salvianolic acid A (Sal A) exhibits anticancer, anti-inflammatory, and cardioprotection effects and is clinically used to treat cancer and various metabolic diseases such as atherosclerosis and diabetes (Qin et al., 2019; Zhu et al., 2022). Notably, research suggested that Sal A exerts hepatoprotective effects through modulation of the AMPK-SIRT1 signaling axis, thereby counteracting hepatic lipid toxicity (Li et al., 2020). Insulin-like growth factors 1(IGF1) and 2 (IGF2) and IGF-binding proteins (IGFBPs) are produced by the liver to regulate metabolism through insulin. This carrier protein IGFBP-1 plays a pivotal role in governing the metabolic fate of IGF1, with its regulatory action directly impacting glycemic control mechanisms and the development of insulin resistance. Studies have shown that Sal A can improve hepatic fatty acid metabolism by activating the AMPK and IGFBP1 pathways. Additionally, activated AMPK can ameliorate inflammation, fibrosis, and mitochondrial dysfunction (Zhu et al., 2024). Hydroxytyrosol (HT) is the primary polyphenol contained in olive oil and leaves and can produce advantageous effects on MAFLD by regulating mitochondrial function. PINK1, as a protein kinase anchored to the mitochondrial membrane, plays a critical role in autophagy and activates parkin ubiquitin ligase activity to facilitate mitophagy. Studies have shown that HT can activate the AMPK/PINK1 pathway to promote mitophagy, thereby enhancing lipid metabolism, reducing oxidative stress, and mitigating mitochondrial dysfunction (Dong et al., 2022). Ellagic acid (EA) is a polyphenolic compound naturally abundant in dicotyledonous plant species, known for its potent anti-inflammatory and antioxidant capacities. C1q/tumor necrosis factor-related protein-3 (CTRP3) is a widely distributed and functionally diverse adipokine that plays a crucial role in endocrine and metabolic diseases such as inflammatory responses, obesity, and type 2 diabetes. Research has found that EA has the potential to treat MAFLD by improving insulin resistance and reducing liver damage through the activation of the AMPK/CTRP3 pathway (Elseweidy et al., 2022). Zingerone (ZIN), isolated from ginger, is a highly effective compound. Studies have found that it can prevent liver deposition and steatosis induced by high-fat feeding in rats by activating the AMPK/Nrf2 axis to counteract oxidative stress and increasing cleaved caspase-3 and Bax/Bcl2 ratios to promote autophagy (Mohammed, 2022). *p*-Coumaric Acid (*p* -CA) is a phenolic acid abundantly present in various edible plants including vegetables, fruits, and fungi, where it exhibits significant antineoplastic activity and oxidative stress-modulating capabilities. Research has found that p-CA reduces serum and liver lipids by activating PPARα/γ and upregulating HSL, HTGL, MGL, CPT1A, and ACSL1 through AMPK activation. Additionally, it inhibits lipid droplet fusion and growth by increasing MSS4 expression levels, thereby treating MAFLD (Yuan et al., 2023a). Studies have found that Androsin alleviates metabolic-associated fatty liver disease (MAFLD) by stimulating AMPKα, which in turn activates the SREBP-1c/FASN pathway to inhibit DNL and the AMPKα/PI3K/Beclin1/LC3 pathway to activate autophagy (Singh et al., 2024). Rosmarinic Acid (RA) and Chicoric Acid (CA) primarily treat metabolic-associated fatty liver disease (MAFLD) by activating AMPK, which inhibits lipogenesis, promotes fatty acid β-oxidation, and improves oxidative stress and inflammation (Ding et al., 2020a; Kim M. et al., 2020).

### 3.5 Quinones

**FIGURE 6:**
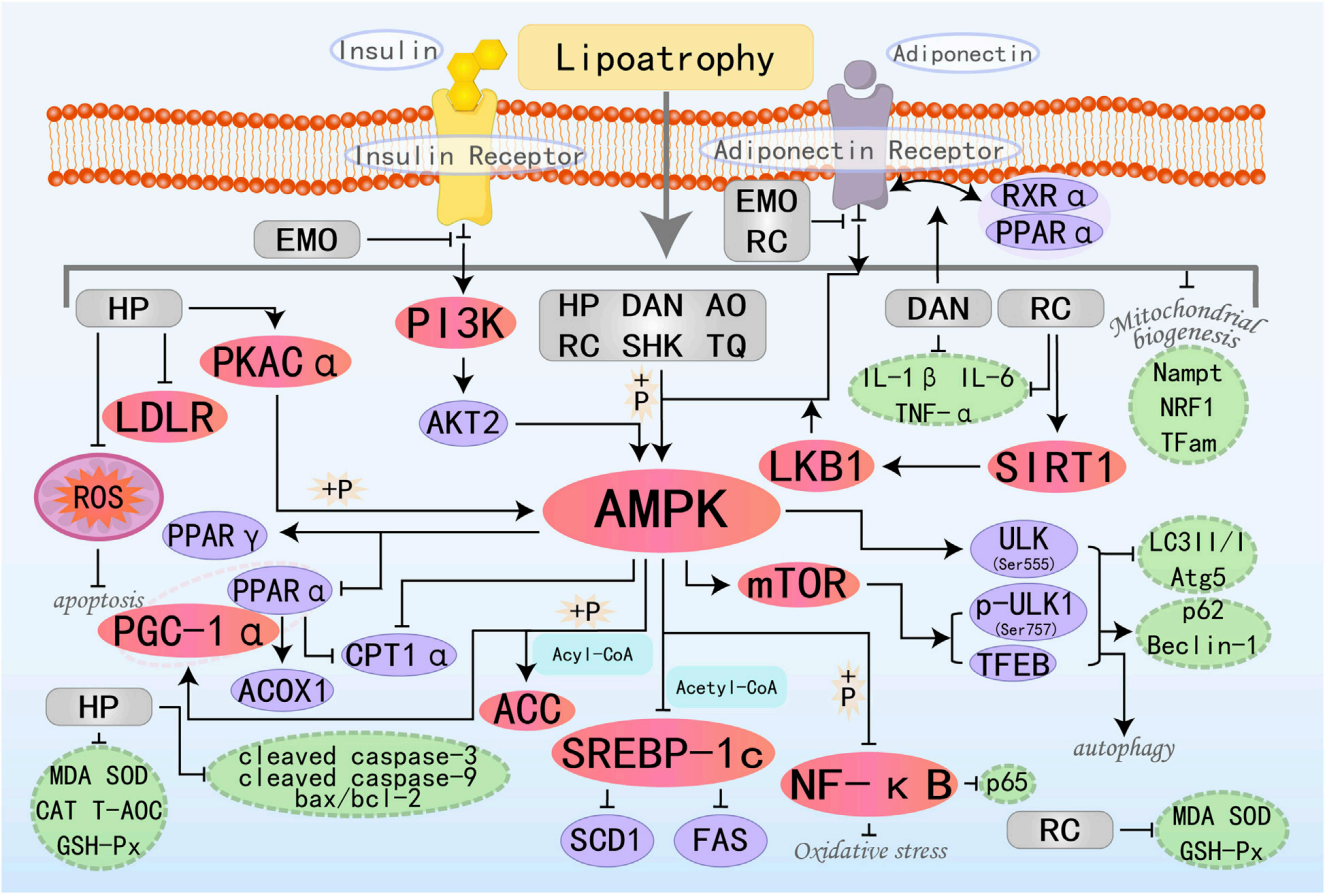
Molecular mechanism of quinone metabolites from botanical drugs targeting AMPK to treat MAFLD. HP, Hypericin; EMO, Emodin; DAN, Danthron; AO, Aurantio-Obtusin; RC, Rhinacanthin C; TQ, Thymoquinone; SHK, Shikonin.

Quinones are natural products widely distributed in nature, collectively referring to a class of organic metabolites containing cyclohexadienedione or cyclohexadienedimethylene structures. Quinones can trigger cytoprotective effects through multiple mechanisms: activation of detoxification enzyme systems, modulation of anti-inflammatory signaling pathways, and remodeling of intracellular redox homeostasis. Studies have found that quinones also have some therapeutic potential for MAFLD (Bolton and Dunlap, 2017). The specific molecular mechanisms by which seven quinone substances treat MAFLD through the AMPK pathway are illustrated in Figure 6.

Emodin (EMO) is an anthraquinone derivative derived from various herbal medicines. EMO possesses a wide range of pharmacological properties, including anticancer, hepatoprotective, anti-inflammatory, and antioxidant activities (Dong et al., 2016). Research by Yu LY et al. has found that EMO is the principal bioactive component in *Radix Polygoni Multiflori Preparata* (RPMP) for the therapy of MAFLD. EMO can reduce hepatic lipogenesis and increase insulin sensitivity to combat insulin resistance (IR) by upregulating phosphatidylinositol 3-kinase (PI3K), AKT2, and AMPKα. Additionally, EMO can promote the binding of adiponectin to AdipoR2, thereby activating AMPK-mediated FAO, ultimately improving hepatic lipid accumulation (Yu et al., 2020). Danthron (DAN) is one of the active metabolites found in the Chinese herbal medicine *Rheum rhaponticum L.* Previous studies have shown that DAN can activate AMPK and regulate lipid and glucose metabolism *in vitro*, making it a potentially effective compound for the treatment of obesity and MAFLD (Zhou et al., 2013). PPARα, as a nuclear receptor, coordinates FAO and maintains mitochondrial homeostasis, while the retinoid X receptor (RXR) functions as the indispensable heterodimeric partner for PPARα activity. Recent research by Ma C et al. has found that DAN can enhance nuclear receptor crosstalk between PPARα and RXRα. Additionally, RXRα promotes the upregulation of AdipoR2 by DAN. The ultimately activated AdipoR2 then promotes the expression of AMPKα and PPARα, ultimately restoring mitochondrial biogenesis to enhance FAO (Ma et al., 2021). Aurantio-Obtusin (AO), primarily derived from cassia seed extract, is a major active ingredient within the anthraquinone class. Studies have confirmed that AO activates autophagy and improves lipid accumulation in the liver by upregulating the expression of a series of autophagy-related proteins, including AMPK, mTORC1, ULK, and TFEB (Zhou et al., 2022). Thymoquinone (TQ) is the primary active ingredient isolated from Nigella sativa, and numerous previous *in vivo* and *in vitro* studies have demonstrated its diverse pharmacological activities, including anti-inflammatory, anti-cancer, antioxidant, and neuroprotective effects (Woo et al., 2012; Mahmoud and Abdelrazek, 2019; Isaev et al., 2020). Recent research has found that TQ also holds great potential in the treatment of MAFLD. ULK1 is an autophagy-initiating kinase that can be oppositely regulated by mTOR and AMPK to initiate autophagy (Alers et al., 2012). Studies by Zhang D et al. have shown that TQ triggers autophagy through the activation of the AMPK/ULK1(Ser555) and AMPK/mTOR/ULK1(Ser757) pathway-dependent mechanisms, thereby reducing body weight, alleviating hepatic steatosis, and improving glucose homeostasis (Zhang D. et al., 2023). Shikonin (SHK) is a natural active ingredient with anti-inflammatory and antioxidant properties. Research has found that SHK can act as an AMPK agonist, activating AMPK to inhibit fat synthesis while also promoting the cooperation between PGC-1α and PPARα, inducing mitochondrial FAO. It has certain preventive and therapeutic effects on liver lipid metabolism and MAFLD (Gwon et al., 2020).

### 3.6 Terpenoids

**FIGURE 7:**
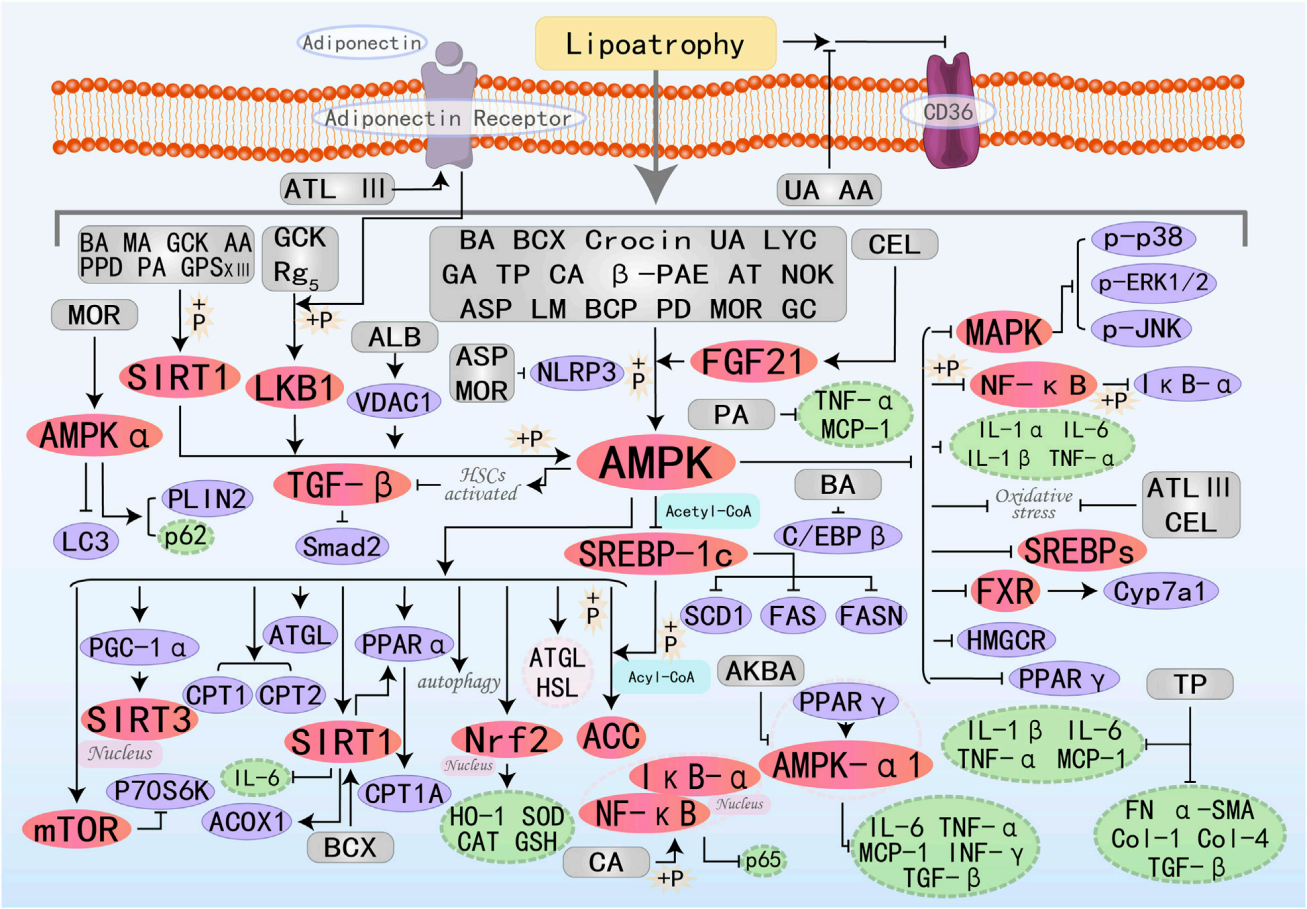
Molecular mechanism of terpenoid metabolites from botanical drugs targeting AMPK to treat MAFLD. BA, Betulinic acid; BCX, β-Cryptoxanthin; MA, Maslinic acid; UA, Ursolic acid; GA, Ganoderic acid A; LYC, Lycopene; TP, Triptolide; CA, Corosolic acid; PA, Patchouli alcohol; β-PAE, β-patchoulene; AA, Arjunolic acid; CEL, Celastrol; ATL III, Atractylenolide III; AT, Astaxanthin; LM, Limonin; NOK, Nootkatone; GCK, Ginsenoside CK; AKBA, Acetyl-11-Keto-Beta-Boswellic Acid; PPD, Protopanaxadiol; ASP, Asperuloside; GPS XIII, Gypenoside XIII; ALB, Alisol B; BCP, β-Caryophyllene; Rg_5_, Ginsenoside Rg_5_; PD, Platycodon D; MOR, Morroniside; GC, Ginkgolide C.

Terpenoids are one of the most extensive and structural variability classes of both essential and specialized metabolites in nature. All terpenoids are formed by linking multiple isoprene units in a head-to-tail manner and exhibit a range of effects, including anti-inflammatory, antioxidant, antitumor, and immunomodulatory properties (Bergman et al., 2024; Khan et al., 2024). The specific molecular mechanisms by which 28 terpenoids treat MAFLD through the AMPK pathway are illustrated in Figure 7.

β-Cryptoxanthin (BCX) is a provitamin A carotenoid with diverse biological activities, capable of treating numerous diseases, including neoplasm and osteoporosis. Latest research has found its significant potential in treating fatty liver disease (Yamaguchi, 2012; Burri et al., 2016; Clugston, 2023). β-Carotene-15,15′-oxygenase (BCO1) and β-carotene-9′,10′-oxygenase (BCO2) can cleave BCX to generate therapeutic metabolic derivatives, including vitamin A. Experimental evidence elucidates BCX’s capacity to enhance hepatic lipid homeostasis via multi-target regulatory effects on the FXR-SIRT1-AMPK signaling axis, demonstrating significant cholesterol metabolic modulation. Specifically, it depends on the presence or absence of BCO1/BCO2 (Lim et al., 2019). Maslinic acid (MA) (Liou et al., 2019a), Crocin (Luo et al., 2019), and Ursolic acid (UA) (Cheng et al., 2020) all improve hepatic steatosis and treat MAFLD by activating the AMPK pathway, promoting fatty acid oxidation, lipolysis, and inhibiting fat synthesis. Lycopene (LYC) is a lipophilic antioxidant carotenoid derived from tomatoes. Research by Wang J et al. has demonstrated that LYC can reduce lipid synthesis, restore mitochondrial function, and ultimately decrease hepatic lipid accumulation to treat MAFLD by increasing PPARα expression and promoting the activation of the AMPK/SIRT1/PGC1α pathway (Wang J. et al., 2020). Patchouli alcohol (PA) is a characteristic tricyclic terpenoid compound naturally occurring in the *Pogostemon cablin*. Previous studies have shown that it has numerous effects, including anti-inflammatory, anti-cancer, anti-depressant, and anti-viral properties (Lee et al., 2020). Research by Pyun D et al. has found that PA also has potential in hepatic lipid metabolism: it activates the AMPK/SIRT1 pathway to inhibit cellular inflammation (such as TNF-α, MCP-1), improve insulin resistance (IRS-1, HOMA-IR index, IPGTT, and ITT), and positively regulate FAO (Pyun et al., 2021). Arjunolic acid (AA) exhibits antioxidant, anti-inflammatory, and free radical scavenging activities (Hemalatha et al., 2010). Recently, Zheng X et al. discovered that AA indirectly activates SIRT1/AMPK-regulated lipid metabolism by increasing NAMPT-mediated NAD + levels and triggering autophagy, collectively mediating lipid-lowering effects (Zheng et al., 2021). Celastrol (CEL) is a pentacyclic triterpenoid compound separated from the Chinese medicinal plant *Celastrus orbiculatus Thunb.*, possesses various pharmacological activities, including anti-tumor and anti-inflammatory effects. Currently, it is considered to have broad application prospects in metabolic diseases, such as in the treatment of type 2 diabetes, atherosclerosis, cholestasis, and osteoporosis (Xu S. et al., 2021). FGF21 is predominantly secreted by hepatic tissues to coordinate glycemic regulation across both hepatic and adipose metabolic networks, providing protective benefits. Recently, Xue JL et al. explored that CEL treatment can lead to improved mitochondrial morphology, liver lipid accumulation, oxidative stress, and inflammation by activating the FGF21/AMPK/PGC-1α signaling pathway, thereby protecting against MAFLD (Xue et al., 2024a). Atractylenolide III(ATL III) is a natural monomeric herbal bioactive compound with extensive effects in antioxidation and anti-inflammation (Xu et al., 2023). Li Q et al. discovered that ATL III treatment *in vitro* activates the AMPK/SIRT1 signaling pathway downstream of AdipoR1, thereby enhancing oxidative stress resistance (SIRT3, NRF2) and FAO(CPT1A, PGC-1α), ultimately protecting the liver (Li et al., 2022c). Ginsenoside CK(GCK) is the primary intestinal metabolite of protopanaxadiol saponins and exhibits multiple therapeutic effects. Zhang JJ et al. found that GCK can activate LKB1 and AMPK phosphorylation, increase ATGL and SIRT1 expression, and inhibit SREBP-1c activity, thereby promoting lipolysis and FAO while suppressing fat synthesis (Zhang J. J. et al., 2022). Both Protopanaxadiol (PPD) (Li Y. et al., 2023) and Gypenoside XIII(GPS XIII) (Cheng et al., 2024) can reduce lipogenesis, increase lipolysis, and enhance fatty acid β-oxidation by increasing SIRT1 and AMPK phosphorylation, thereby regulating downstream molecules. Alisol B (ALB) is a triterpenoid monomer isolated from classic Chinese medicinal herbs, which plays a role in inhibiting lipogenesis and reducing subcutaneous adipose tissue mass. VDAC1, a voltage-dependent anion channel protein, maintains the balance of the intracellular and extracellular environment by regulating mitochondrial permeability. Gao G et al. found that ALB directly targets VDAC1 to increase the ADP/ATP and AMP/ATP ratios, thereby modulating the AMPK/mTOR/SREBPs pathway to inhibit lipid synthesis (Gao et al., 2024). Ginsenoside Rg5 (Rg5) activates the LKB1/AMPK/mTOR signaling pathway, stimulating energy metabolism and thereby impeding the progression of MAFLD (Shi et al., 2024). Morroniside (MOR) inhibits the progression of NASH by promoting AMPK-dependent lipophagy and inhibiting NLRP3 inflammasome activation (Zhang et al., 2024). β-Caryophyllene (BCP) (Kamikubo et al., 2024) and Ginkgolide C (GC) (Xie et al., 2024) can act as AMPK agonists, regulating downstream molecules involved in lipid synthesis and FAO by activating the AMPK signaling pathway, ultimately improving MAFLD.

### 3.7 Glycosides

**FIGURE 8:**
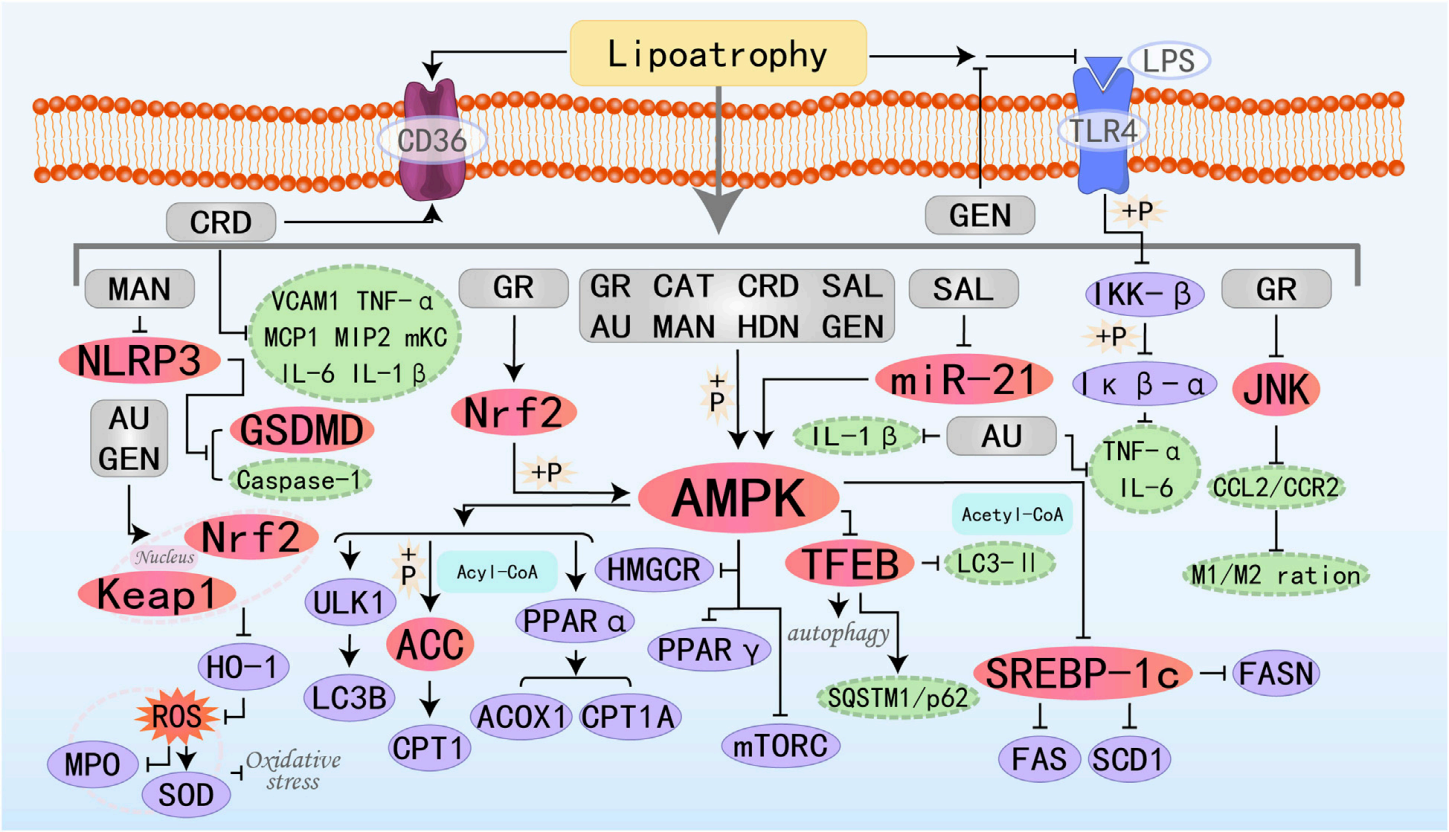
Molecular mechanism of glycoside metabolites from botanical drugs targeting AMPK to treat MAFLD. GR, Glucoraphanin; CAT, Catalpol; AU, Aucubin; CRD, Cordycepin; SAL, Salidroside; MAN, Mangiferin; HDN, Hesperidin; GEN, Geniposide.

Glycosides, also known as “saponins” or “glycosides,” are molecules in which one part is linked to a sugar moiety, while the other part, which is non-sugar, is called the aglycone. Glycosides exhibit extensive application prospects in the medical field, demonstrating favorable pharmacological effects in regulating blood glucose and lipids, as well as possessing antitumor activity, which can be used as adjuvant therapy for cancer. The specific molecular mechanisms by which eight glycosides treat MAFLD through the AMPK pathway are illustrated in Figure 8.

Catalpol (CAT), is a functional substance derived from *Rehmannia glutinosa*. It is commonly used in the treatment of various inflammatory diseases, diabetes, cardiovascular and cerebrovascular diseases, among others (Yan et al., 2018; Bhattamisra et al., 2021; Liu J. et al., 2023; Zhang Z. et al., 2023). Transcription Factor EB (TFEB) is a master regulator whose activation promotes the transcription of genes involved in lysosomal network expansion and regulates autophagy when translocated to the nucleus. CAT can significantly upregulate autophagy-related genes (including Atg7, Atg5, Becn1, Ulk1, and Lamp1) to induce autophagy and regulate hepatic lipid metabolism genes (ACC1α, FAS, PPARα, ACOX1, and CPT1) to improve hepatic lipid accumulation by activating the AMPK/TFEB pathway, and it is a novel therapeutic candidate for MAFLD (Ren et al., 2019; Tian et al., 2020). Aucubin (AU) is an iridoid glycoside derived from natural plants, possessing anti-inflammatory, antioxidant, and anti-fibrotic properties. It has broad application potential in the treatment of atherosclerosis, fatty liver disease, acute hepatitis, diabetes, and other conditions (Wang H. et al., 2020; Bao et al., 2022; Huang et al., 2022; Liu et al., 2022). Shen B et al. found that the protective effect of AU on MAFLD may be exerted by promoting the escape of Nrf2 from the control of Keap1 and its translocation to the nucleus, which in turn inhibits oxidative stress (reduction of ROS, increase in SOD levels, and decrease in MPO levels) (Shen et al., 2019b). Cordycepin (CRD) is a bioactive compound extracted from Cordyceps sinensis, possessing multiple pharmacological effects. Recent *in vivo* studies have also shown that CRD primarily treats MAFLD by activating AMPK, regulating the expression of key genes related to lipid metabolism (such as SREBP1-c, ACC, SCD-1, LXRα, and CD36), as well as β-oxidation genes (CPT-1 and PPARα), and improving the inflammatory state (Gong et al., 2021). Salidroside (SAL) is also an AMPK agonist that prevents the progression of NASH induced by metabolic stress, inflammation, and other factors by activating AMPK signaling (Hu et al., 2021). Geniposide, an iridoid glycoside extracted from the fruit of Gardenia jasminoides, holds great potential in improving glucose and lipid metabolism (Gao and Feng, 2022). Research by Yi M et al. has demonstrated that GEN can inhibit the inflammatory response induced by LPS directly binding to TLR4 protein and activating NF-κB. Additionally, it regulates lipid metabolism through the AMPK/ACC/CPT1A and AMPK/ULK1/LC3B signaling pathways, thereby preventing and treating MAFLD (Yi et al., 2023).

### 3.8 Stilbenes

**FIGURE 9:**
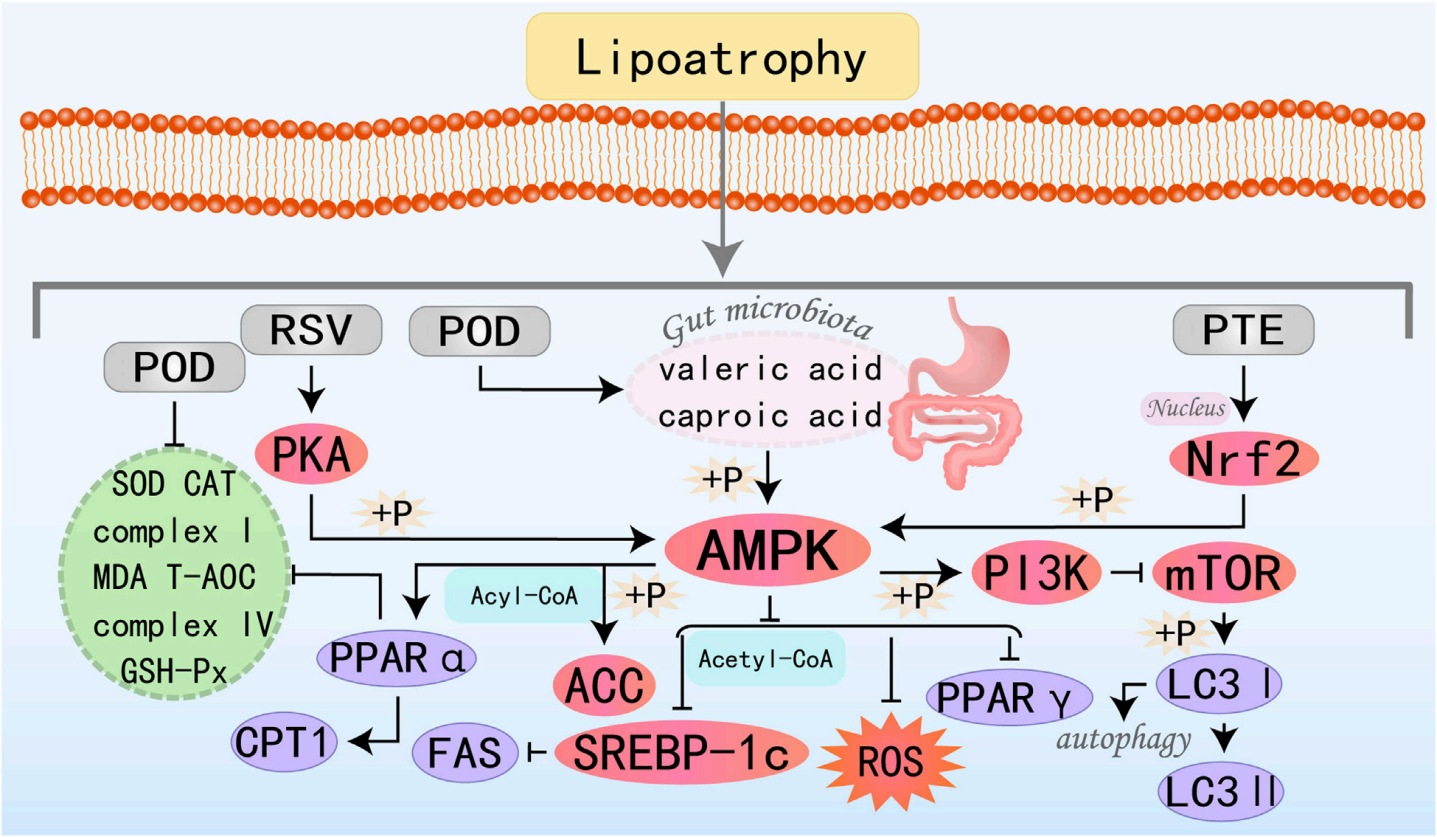
Molecular mechanism of stilbenes metabolites from botanical drugs targeting AMPK to treat MAFLD. RSV, Resveratrol; POD, Polydatin; PTE, Pterostilbene.

Stilbenoids constitute a group of metabolites defined by their stilbene core structure or polymeric derivatives, functioning as phenolic secondary metabolites in plants. Among these, the most widely recognized is resveratrol, known for its roles as a cardioprotective agent, potent antioxidant, anti-inflammatory agent, and anticancer agent, among others. Current research on the stilbene scaffold continues with the aim of discovering new analogs with higher bioavailability. The specific molecular mechanisms by which three stilbenoid metabolites defend against MAFLD through the AMPK pathway are illustrated in the accompanying Figure 9.

Resveratrol (RSV), a polyphenolic compound of significant research interest, exhibits pleiotropic effects such as quenching oxidative stress, resolving inflammatory cascades, reducing blood pressure and blood sugar levels, combating aging, and exerting anticancer effects. Clinically, it can be utilized in the treatment of metabolic diseases, cardiovascular diseases, and various types of tumors (Ren et al., 2021; Zhang et al., 2021a; Zhou et al., 2021). Research by Huang YJ et al. has shown that RSV modulates lipid metabolism and redox homeostasis by regulating CPT-1, SREBP-1c, and FAS through the PKA/AMPK/PPARα pathway, indicating significant potential for the prevention and treatment of MAFLD (Huang et al., 2020). Polydatin (POD), a glucoside derivative of RSV, boasts higher bioavailability and is primarily involved in modulating homeostatic regulation such as inflammation, oxidative stress, and apoptosis. It plays a significant role in the prevention and treatment of tumors, cardiovascular diseases, and metabolic disorders including diabetes, NASH, and fibrosis (Li et al., 2018; Karami et al., 2022). Research by Zhao G et al. has discovered that POD can significantly elevate the levels of valeric acid and caproic acid in feces by modulating the gut microbiota, thereby activating the AMPK. This activation leads to a reduction in lipid accumulation in the liver and serum, thereby ameliorating MAFLD (Li et al., 2018). Pterostilbene (PTE) is a dimethylated analog of RSV, endowed with physiological activities such as anti-inflammatory, antioxidative stress, and anticancer properties (Estrela et al., 2013; Kim H. et al., 2020; Lin et al., 2020; Gómez-Zorita et al., 2021). Recent research by Shen B et al. has revealed that PTE promotes the nuclear translocation of Nrf2, induces AMPK phosphorylation through Nrf2, and subsequently promotes ACC phosphorylation while inhibiting mTORC, among others. Ultimately, it enhances autophagy, suppresses oxidative stress, and promotes the metabolism and breakdown of fatty acids, thereby ameliorating MAFLD (Shen et al., 2023a).

### 3.9 Others

**FIGURE 10:**
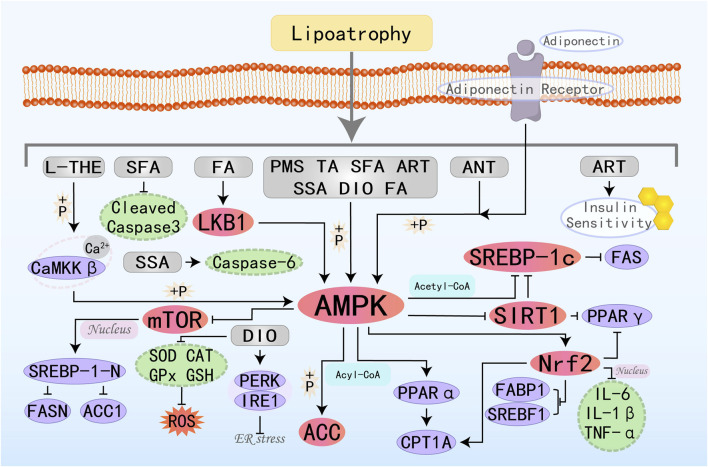
Molecular mechanism of other metabolites from botanical drugs targeting AMPK to treat MAFLD. L-THE, L-theanine; PMS, Plantamajoside; TA, Tartaric acid; SFA, Sulforaphane; ART, Atractylodin; ANT, Antrodan; SSA, Salsalate; DIO, Diosgenin; FA, Folic acid.

In addition to the eight bioactive metabolites extracted from phytomedicines mentioned above, other types of natural active monomers have also been found to improve MAFLD. The specific molecular mechanisms by which nine other metabolites defend against MAFLD through the AMPK pathway are illustrated in Figure 10.

L-Theanine (L-THE), a natural component derived from tea, exhibits immune-regulatory and sedative effects and is commonly used in the management of many psychiatric disorders (Chen S. et al., 2023). Research by Liang J et al. has demonstrated that L-theanine ameliorates hepatic steatosis by boosting the Ca^2+^-CaMKKβ-AMPK signaling pathway, thereby modulating hepatocyte lipid metabolism pathways (Liang J. et al., 2022). Plantamajoside (PMS), the primary active ingredient in *Plantago asiatica L.*, exhibits various biological activities. It can improve immune dysregulation (downregulate IL-6, IL-1β, TNF-α), reduce fatty acid uptake (downregulate FABP1), and ameliorate abnormal liver lipid metabolism (inhibit SREBF1, PPARγ) in MAFLD rats by activating the AMPK/Nrf2 pathway, thereby treating MAFLD (Wu et al., 2023). Antrodan (ANT) has been shown to effectively alleviate MAFLD through the AMPK/SIRT1/SREBP-1c/PPARγ pathway (Chyau et al., 2020). Diosgenin (DIO) is a natural saponin, and research findings consistently highlight that DIO can treat metabolic diseases through various pathways and mechanisms (Zhang S. Z. et al., 2022). DIO inhibits DNL and increases FAO by modulating the AMPK-ACC/SREBP1 pathway. Additionally, it can suppress ER stress by regulating the PERK and IRE1 branches, reduce ROS by increasing levels of SOD, CAT, and GPx, and enhance antioxidant capacity, thereby offering therapeutic benefits for MAFLD (Zhong et al., 2022).

## 4 Discussion

MAFLD is a term introduced in 2020 as an improvement over the previous nomenclature of NAFLD, aiming to capture the metabolic essence more precisely. In terms of diagnosis, MAFLD is identified based on metabolic abnormalities such as type 2 diabetes, obesity, or metabolic syndrome, and it is compatible with other hepatic disorders (e.g., viral hepatitis), making the diagnosis more comprehensive and practical. From a clinical perspective, the diagnostic criteria for MAFLD are better at recognizing populations susceptible to liver fibrosis and metabolic complications and are more intuitive, which helps to enhance understanding of the disease among the public and primary care physicians. The pathogenesis of MAFLD is highly complex, involving multiple processes such as DNL, FAO, oxidative stress, inflammatory responses, autophagy, and endoplasmic reticulum stress. These factors interact, ultimately leading to hepatic fat deposition, inflammation, and fibrosis, which may progress to cirrhosis and hepatocellular carcinoma. It is the complexity and diversity of MAFLD pathogenesis and disease progression that makes drug therapy a challenge, including the difficulty of achieving significant efficacy with single-mechanism drugs, the lack of drugs that are effective at all stages of the disease, the difficulty of identifying drug targets, and the side effects and safety issues of drugs. Consequently, future studies should prioritize investigating the synergy of multidrug combinations to optimize treatment outcomes and minimize adverse reactions, as well as continuing the search and development of novel drug targets directing the pathogenesis of MAFLD. AMPK is a crucial regulatory enzyme for glucose and lipid metabolism, capable of modulating energy metabolism, lipid metabolism, and glucose metabolism. Therefore, it holds potential application value in the treatment of MAFLD. Despite progress, pharmacological interventions targeting AMPK for MAFLD management are still in early-phase development. Compared to Western medicine, natural plant medicine has garnered growing interest in the treatment of MAFLD due to its multi-target, multi-pathway mechanisms of action, as well as its minimal side effects and cost-effectiveness. Emerging evidence from recent investigations suggests natural active botanical metabolites functioning as AMPK activators could represent novel strategies for both prophylactic and therapeutic management of MAFLD. With this goal in view, this study summarizes the *in vivo* and *in vitro* experimental literature from the past 5 years on Natural Active Botanical metabolites improving MAFLD by targeting the AMPK pathway. In this review, we conclude that natural active botanical metabolites ameliorate hepatic lipid accumulation and degeneration, ultimately treating MAFLD, by activating AMPK and its related pathways, inhibiting lipogenesis, exerting anti-inflammatory and antioxidant effects, promoting fatty acid oxidation, lipolysis, autophagy, and improving insulin resistance.

Specifically, natural active botanical metabolites can activate AMPK and increase its phosphorylation by modulating upstream molecules such as kinases (e.g., LKB1 and CaMKK2), energy sensors (AMP), and other upstream regulators (e.g., leptin, adiponectin). They can also directly activate AMPK, thereby inhibiting the activity of lipogenic gene targets like ACC, HMGR, SCD1, and SREBP-1c, and promoting the expression of genes involved in FAO such as PPARα, CPT1α, and ACOX1. Additionally, they improve mitochondrial function, restore mitochondrial homeostasis, regulate oxidative stress-related factors (e.g., ROS, MDA, SOD, GSH-Px, CAT), and modulate autophagy factors (e.g., Bax, p62, cleaved caspase 3, Bcl-2, Atg7, LC3II/I, Beclin1). They also reduce inflammatory cytokines like IL-1β, IL-6, TNF-α, and MCP-1, suppress endoplasmic reticulum stress (downregulating BiP, ATF6, CHOP, ERK, JNK, etc.), promote the expression of HSL and ATGL to accelerate lipolysis, and regulate AUC of ITT, GTT, HOMA-IR index, fasting blood glucose, insulin level and so on to improve the body’s insulin resistance, and finally play a role in the treatment of MAFLD. In addition, α-SMA, TGF-β, Col-1, and Col-4 can be regulated to prevent liver fibrosis, prevent the risk of complications, and improve the patient’s quality of life and prognosis. The pathways involved are diverse, including AMPK/SREBP1, AMPK/ACC, AMPK/PGC-1α, LKB1/AMPK, SIRT1/AMPK, AMPK/mTOR, AMPK/ULK1, AMPK/NF-κB, AMPK/Nrf2, AMPK/PINK1, and TGFβ-Smad2/3. Natural plant medicine, with its multi-pathway, multi-level, and multi-target characteristics, acts on various links within these pathways, forming a complex network that collectively promotes the alleviation of MAFLD.

Our study demonstrates that natural active botanical metabolites currently targeting MAFLD treatment are predominantly clustered in flavonoids and terpenoids. Flavonoids typically exert their therapeutic effects through multi-target mechanisms, with particularly significant roles in modulating oxidative stress (such as baicalein, baicalin, neohesperidin, and hesperetin), regulating lipid metabolism, and combating inflammatory pathways (such as quercetin, chrysin, and 6-Gingerol). However, we do not know which flavonoids are the most effective or which ones are suitable for dietary therapy. Therefore, more clinical trials are needed to validate this. Our study also demonstrates that multiple terpenoids exhibit a certain degree of convergence in their targeted signaling pathways and molecular mechanisms when treating MAFLD. For example, by modulating PPAR-α, PPAR-γ, Nrf2, and SIRT1, they exert antioxidant, anti-inflammatory, and hepatoprotective effects. Furthermore, alkaloids, phenolic acids, and quinones also demonstrate considerable therapeutic potential, while lignans and stilbenes remain relatively understudied, warranting focused exploration as promising yet underexplored candidates.

Furthermore, it is noteworthy that natural active botanical metabolites demonstrate promising therapeutic potential in the treatment of MAFLD, their clinical application faces multiple challenges: (1) physicochemical limitations: natural monomers often suffer from poor aqueous solubility and strong lipophilicity, leading to restricted oral bioavailability (primarily due to significant hepatic first-pass effects); (2) targeted delivery barriers: inefficient hepatic accumulation and nonspecific systemic distribution may result in off-target effects; (3) pharmacodynamic shortcomings: the lack of tissue-specific targeting capability hampers precise modulation of metabolic dysregulation pathways in the liver. These factors collectively impede their clinical translation. Emerging strategies such as hepatic-targeted drug delivery systems (HTDDS), derivatization, and structural modifications are progressively addressing these issues. These approaches not only enhance drug delivery efficiency and stability but also improve therapeutic efficacy and safety profiles (Tang et al., 2021). In typical situations, the solubility, absorption, and metabolism rates of dietary flavonoids are low, while flavonoid nanoparticles and flavonoid-metal ion complexes not only enhance their effects but also reduce the systemic toxicity side effects of the drugs, demonstrating great potential in the treatment of MAFLD (Selvaraj et al., 2014; Dobrzynska et al., 2020). For example, quercetin-iron complex nanoparticles can significantly improve the stability and solubility of quercetin while also enhancing its antioxidant capacity (Prestianni et al., 2023). Silybin is treated with nanotechnologies such as nanoparticles, liposomes, and nano-suspensions, as well as CD44 receptors, folic acid, vitamin A, and other liver-targeting methods to treat various liver diseases more effectively (Wu et al., 2024). Based on these significant research findings, we are confident in the development prospects of individual metabolites of natural plant medicine in the field of MAFLD treatment.

Natural active botanical metabolites, leveraging their unique advantages of multi-pathway and multi-target actions, have demonstrated remarkable efficacy in improving MAFLD. These metabolites are not only economical and efficient but also exhibit relatively few side effects. In numerous experimental studies focused on weight control, liver tissue repair, and improvements in TC, TG, LDL-C, and HDL-C levels, natural active botanical metabolites have shown a broad and effective therapeutic dose range. However, there are still some pressing issues in current research in this field. Firstly, in developing approaches for the intervention and therapy of MAFLD, existing experimental studies primarily focus on therapeutic effects and their mechanisms, while assessments of the safety and toxicological characteristics of these metabolites are relatively inadequate. Therefore, there is an urgent need to strengthen systematic safety evaluations of individual metabolites from natural plant medicine and conduct standardized toxicity studies. Secondly, some individual metabolites from natural plant medicine that have been proven to have good therapeutic effects face technical challenges such as poor water solubility, low oral absorption efficiency, and unclear pharmacokinetic profiles, which severely limit their clinical translation and application. Although the development of novel drug delivery systems brings hope for improved liver-targeted drug delivery and enhanced bioavailability, current related research is still mainly confined to animal experiments and *in vitro* cellular studies. The absorption, distribution, metabolism, and excretion processes of these metabolites in humans still require further in-depth research and elucidation. It is particularly important to note that the study is primarily based on animal and cellular research, but lacks clinical trial data, which fails to validate the efficacy, safety, and optimal dosage of these components in humans. Thirdly, Currently, common MAFLD mouse models whether diet-induced (high-fat diet, high-cholesterol diet, methionine, and choline-deficient diet, high-fat, high-cholesterol diets and high-fat, high-fructose diets), genetic (db/db mice, ob/ob mice, and ApoE^−/−^ mice), or chemically induced (CCl4 administration), have certain limitations and fail to meet the characteristics of an “ideal” MAFLD animal model. Furthermore, while some animal models can accurately replicate specific stages of MAFLD, they do not fully reproduce the entire human pathophysiological process. Finally, although natural active metabolites exert preventive and therapeutic effects on MAFLD by acting on the AMPK target, this process is regulated by multiple intertwined and closely related signaling pathways. All conclusions drawn in this study are solely based on the AMPK pathway, overlooking the complexity of the integrated regulatory network.

Given these research gaps, future studies should focus on: (1) advancing clinical translation to validate the efficacy and safety of natural metabolites in humans; (2) systematically investigating long-term toxicity, drug interactions, and effects in special populations; (3) developing nanocarriers and liver-targeting technologies to enhance bioavailability and minimize systemic side effects; and (4) integrating multi-omics approaches to comprehensively map mechanistic networks and clarify multi-target synergistic effects.

Taking all factors into consideration, this article summarizes the significant therapeutic effects of natural active botanical metabolites on MAFLD through targeted regulation of AMPK and its various specific pathway mechanisms. Additionally, we have also pointed out the deficiencies in current research regarding experimental design and subsequent development and application, as well as our future expectations. We believe that if these widely available, low-cost, and complex natural active botanical metabolites can be better utilized, they could provide new and reliable means for the treatment of MAFLD and even various metabolic diseases.

## Glossary

MAFLD: metabolic dysfunction-associated fatty liver disease
NAFLD: non-alcoholic fatty liver disease
NASH: non-alcoholic steatohepatitis
IR: insulin resistance
AMPK: AMP-activated protein kinase
DNL: *de novo* lipogenesis
FAO: fatty acid oxidation
FXR: farnesoid x receptor
PPAR: peroxisome proliferator-activated receptor
THR: thyroid hormone receptor
TG: triglycerides
ACLY: ATP-citrate lyase
ACC: acetyl-CoA carboxylase
LKB1: liver kinase B1
CPT1: Carnitine palmitoyltransferase 1
ATGL: adipose triglyceride lipase
HSL: hormone-sensitive lipase
mTOR: mammalian target of rapamycin
LC3: light chain 3
PGC-1α: peroxisome proliferator-activated receptor gamma coactivator 1-alpha
PPARα: peroxisome proliferator-activated receptor alpha
OS: oxidative stress
ROS: reactive oxygen species
HCC: hepatocellular carcinoma
Nrf2: nucleus factor erythroid 2-related factor 2
ER: endoplasmic reticulum
UPR: unfolded protein response
SCD1: stearoyl-CoA desaturase 1
UCP2: uncoupling protein 2
TAK1: transforming growth factor-β-activated kinase 1
CaMKKβ: calmodulin-dependent protein kinase β
LepR: leptin receptor
IRS-1: insulin receptor substrate-1
AKT: protein kinase B
NRF-1: nuclear respiratory factor-1
STRAD: STE20-related adapter
PLIN2: perilipin 2
CMA: chaperone-mediated autophagy
Drp1: dynamin-related protein 1
PINK1: PTEN-induced kinase 1
IGF1: insulin-like growth factors 1
IGF2: insulin-like growth factors 2
IGFBPs: IGF-binding proteins
CTRP3: C1q/tumor necrosis factor-related protein-3
PI3K: phosphatidylinositol 3-kinase
RXR: retinoid X receptor
BCO1: β-Carotene-15,15′-oxygenase
BCO2: β-carotene-9′,10′-oxygenase
TFEB: transcription Factor EB
