# Supplementary material for: Natural active botanical metabolites: targeting AMPK signaling pathway to treat metabolic dysfunction-associated fatty liver disease
Source: Front Pharmacol. 2025 Jul 14;16:1611400. doi: 10.3389/fphar.2025.1611400 (PMC12301338; doi:10.3389/fphar.2025.1611400)
Supplement: Supplementary file 1 [file Table1.docx]

**Table 1. Potential mechanism of natural active botanical metabolites alleviating MAFLD by activating AMPK signaling pathway**

| **Categories** | **Metabolites** | **Plant sources** | **Family** | **Animals/Cell model** | **Dose/Concentration** | **Control Group** | **Mechanisms** | **Related molecular targets** | **Data support for mitigating MAFLD** | **References** |
| --- | --- | --- | --- | --- | --- | --- | --- | --- | --- | --- |
| **Alkaloid** | Leonurine(C_14_H_21_N_3_O_5_) | *Leonurus japonicus Houtt.* | *Lamiaceae* | C57BL/6 J mice fed by MCD | (50, 100, 200 mg/kg/day, p.o) for 6w  (125, 250, 500 µM) for 24h | - | Reducing the lipid synthesis | MDA, SREBP-1c, Fas, SCD1↓  SOD, GSH, cysteine, SAM: SAH, p-AMPK↑ | lipid accumulation, TC, TG, AST, ALT↓ | (Zhang et al., 2019a) |
|  |  |  |  | HepG2 cells stimulated by PA |  |  |  |  |  |  |
|  |  |  |  | C57BL/6N mice fed by HFHSD | (30 mg/kg/day, i.g) for 12w | Fenofibrate (40 mg/kg, i.g) for 12w | Modulating hepatic lipid metabolism and inhibiting lipid synthesis | p-AMPKα/t-AMPK, ADRA1a ↑  SREBP-1c , ACC1 , FASN ↓ | body weight, GLU, GSP, ALT, LDL-C, TC, TG↓  HDL-C↑ | (Fan et al., 2024) |
|  | Liensinine(C_37_H_42_N_2_O_6_) | *Nelumbo nucifera Gaertn.* | *Nelumbonaceae* | C57BL/6 mice fed by HFD | (15, 30, 60 mg/kg/day, i.g) for 16w | - | Mitigating metabolic disorder, insulin resistance, dyslipidemia, oxidative stress, and inflammatory response | p-AMPK, p-ACC, PPARα, CPT1A , UCP2 , SOD, HO1, NQO1, GCLM, GCLC, Nrf2 ↑  FAS , SCD , PPARγ , ROS, MDA, H2O2, NOX2, NOX4, Keap-1, IL-1β, IL-6, TNF-α, MCP-1, CCL-5, CX3CL1, p-TAK1, p-IκBα, p-NF-κB↓ | lipid deposition, body weight, FBG, insulin, AUC of GTT, AUC of ITT, ALT, AST, AKP, NASH score, steatosis score, ballooning score, inflammation score, TG, TC, NEFA, LW/BW↓ | (Liang et al., 2022b) |
|  |  |  |  | AML12 and L02 cells stimulated by PA | (10, 20, 30 µM) for 24h |  |  |  |  |  |
|  | Berberine(C_20_H_18_NO4+) | Coptis chinensis Franch. | *Ranunculaceae* | Sprague-Dawley rats fed by HFD | (100 mg/kg/day, i.g) for 16w | - | Suppressing lipogenesis and stimulating fatty acid oxidation | p-AMPK , p-ACC , CPT-1A , SIRT3 , SIRT1↑ | body weight, liver weight, TC, TG, LDL-C, ALT, AST↓  HDL-C↑ | (Zhang et al., 2019b) |
|  |  |  |  | C57BL/6 J mice fed by HFD | (300 mg/kg/day, p.o) for 4w | - | Reducing the overnutrition-induced hepatic TG accumulation | SCD1 , SREBP-1c, FAS ↓  p-AMPK, p-SREBP-1c↑ | TG, TC, body weight, AUC of IPITT, AUC of IPGTT↓ | (Zhu et al., 2019) |
|  |  |  |  | HepG2 cells stimulated by PA | (20 µM) for 24h |  |  |  |  |  |
|  |  |  |  | db/db mice | (300 mg/kg/day) for 8w | Metformin (300 mg/kg/day) for 8w | Inhibiting inflammation, oxidative stress, and apoptosis | GSH-Px, GSH, SOD, CAT, Bcl-2, p-AMPK, SIRT1, p-ACC, FOXO1↑  MDA, IL-1β, IL-6, TNF-α, MCP-1, Bax, cleaved caspase 3, NF-κB↓ | body weight, liver weight, liver index, TC, TG, LDL-C, AST, ALT↓  HDL-C↑ | (Chen et al., 2024) |
|  | Oxymatrine(C_15_H_24_N_2_O_2_) | *Sophora flavescens Aiton.* | *Fabaceae* | Sprague-Dawley rats fed by HFHSD | (100 mg/kg/day, i.g) for 8w | - | Controlling the downstream key regulators of lipid synthesis, transport, and degradation | Sirt1, p-AMPKα↑  L-FABP, Plin2, FASN, SCD1↓ | body weight, liver weight, TG, TC, FFA, lipid droplets↓ | (Xu et al., 2020) |
|  | Betaine(C_5_H_11_NO_2_) | *Beta vulgaris L.* | *Amaranthaceae* | ApoE^–/–^ mice fed by HFD | (2% (wt/v) betaine, p.o) for 8w | - | Reducing lipid synthesis and improving fatty acid oxidation | SOD, AMPK , ATGL , LCAT, Rxra, FGF10 ↑  MDA, FASN , SREBF1, NR1H3 , ACC , SIRT1 , PPARγ↓ | body weight, TC, TG, FFA, LDL-C, yellow lipid plaque deposition, AST, ALT↓  HDL-C↑ | (Chen et al., 2021) |
|  |  |  |  | HepG2 cells stimulated by OA | (20, 40, 80, 160 mM) for 48h |  |  |  |  |  |
|  |  |  |  | ICR mice fed by CDAHFD | (0.5%, 1% betaine, p.o) for 1w | - | Inhibiting steatosis, ER stress, and apoptosis | TBARS, CDO, ER stress markers, BiP, ATF6, CHOP, p-ERK, p-JNK, P62, HCY↓  BHMT, MAT1A, CβS, CγL, SAM/SAH, PEMT, p-AMPK, p-ACC, Atg7, LC3II/I, Beclin1, p-mTOR, PPARα, ACS1↑ | ALT, LW/BW, TG↓ | (Seo et al., 2024) |
|  | Berbamine(C_37_H_40_N_2_O_6_) | *Phellodendron chinense Schneid* | *Rutaceae* | Wistar rats fed by HFD | (50, 150 mg/kg/day, p.o) for 4w | Fenofibrate (100 mg/kg/day, p.o) for 4w | Promoting fatty acid oxidation, inhibiting lipogenesis, and inducing autophagy | LPO, p-mTOR, SREBP-1c, FAS, SCD1, ChREBP-α, LC3A/B , p62↓  SOD, HO-1, SIRT1 , LKB1 , AMPK , p-AMPK, PPAR-α, p-ACC, CD36, Beclin 1↑ | body weight, liver index, visceral fat, epididymal fat, AST, ALT, ALP, TG, TC, serum glucose, lipid droplets, ballooning degeneration, vacuolated cytoplasm↓  albumin/globulin, hepatic glycogen↑ | (Sharma et al., 2021) |
|  | Tomatidine(C_27_H_45_NO_2_) | *Solanum lycopersicum L.* | *Solanaceae* | C57BL/6 mice fed by HFD | (5, 10 mg/kg/day, p.o) for 4w | - | Enhancing fatty acid β-oxidation, increasing lipolysis, and reducing oxidative stress damage | Srebp-1c, C/EBPβ, FAS↓  ATGL, HSL, sirt1, CPT-1, PPAR-α, p-AMPK, p-ACC↑ | body weight, adipocyte size, lipid droplets, glycogen, NAFLD scores, TG, TC, AST, ALT, HOMA-IR, leptin, adiponectin, lipid droplet accumulation↓ | (Wu et al., 2021) |
|  |  |  |  | FL83B cells stimulated by OA | (3, 10 µM) for 24h |  |  |  |  |  |
|  | Neferine(C_38_H_44_N_2_O_6_) | *Nelumbo nucifera Gaertn.* | *Nelumbonaceae* | C57BL/6 mice fed by HFD | (5, 10 mg/kg/day, i.p) for 4w | Obeticholic acid (10 mg/kg/day, i,g) for 4w | Increasing fatty acid oxidation, alleviating inflammation | FAS, SREBP-1C , PPARγ, TLR4, NF-κB p65, IL-18, TNF-α, TLR4, IL-1β, NLRP3, IL-6, FASN , SCD-1 , Col1a1 , Col3a1 , α-sma , Tgfb , Timp1 ↓  SIRT1, ATGL, p-AMPK, p-ACC, Atgl, Cpt1a, PPARα↑ | lipid droplet accumulation, liver weight, lipid droplets, TC, TG, AST, ALT, LDL-C↓  HDL-C↑ | (Wang et al., 2023) |
|  |  |  |  | HepG2 cells stimulated by OA | (12.5, 25 µM) for 24h |  |  |  |  |  |
|  | Tetrahydropalmatine(C_21_H_25_NO_4_) | *Corydalis yanhusuo (Y.H.Chou & Chun C.Hsu) W.T.Wang ex Z.Y.Su & C.Y.Wu* | *Papaveraceae* | C57BL/6 J mice fed by HFD | (40, 80 mg/kg/day, p.o) for 16w | - | Promoting mitochondrial β-oxidation | Fas, ACC, SREBP-1c ↓  CPT-1A , PGC-1α, AMPK , PPARα, Sirt1, p-AMPK, HMGCR, TBC1D1 ↑ | lipid droplet accumulation, liver weight, body weight, TG, T-CHO, LDL-C, AST, ALT↓  HDL-C↑ | (Yin et al., 2023) |
|  |  |  |  | SMMC-7721 stimulated by FFA, BEL-7402 cells stimulated by FFA | (50, 75, 100 µM) for 24h |  |  |  |  |  |
| **Flavonoid** | Vitexin(C_21_H_20_O_10_) | *Crataegus pinnatifida Bunge* | *Rosaceae* | C57BL/6J mice fed by HFD | (10, 20 mg/kg/day, p.o) for 8w | - | Inhibiting lipogenesis and activating lipolysis and fatty acid oxidation | PPARγ, FAS, ACC, SREBP-1c, p-IRS-1↓  ATGL, PPARα，PGC-1α, CPT-1α, p-AKT, LepR↑ | body weight, liver weight, TG, TC, AST, ALT↓ | (Inamdar et al., 2019) |
|  | Licochalcone A(C_21_H_22_O_4_) | *Glycyrrhiza uralensis Fisch. ex DC.* | *Fabaceae* | C57BL/6 mice fed by HFD | (5, 10 mg/kg/day, i.p) for 12w | - | Enhancing the lipolysis of fatty liver cells and blocking fatty acid uptake to decrease lipid accumulation | TNF-α, SREBP-1c, PPAR-γ, FAS, C/EBPα↓  p-HSL, p-ATGL, CPT-1, p-sirt1，p-AMPKα, p-ACC-1↑ | adipocyte sizes, liver weight, body weight, TC, TG, NAFLD scores, GOT, GPT, LDL, HOMA-IR, leptin, glucose, insulin, lipid droplets, fatty acid uptake↓  HDL, adiponectin↑ | (Liou et al., 2019b) |
|  |  |  |  | HepG2 cells stimulated by OA | (1.5-12 µM) for 12h |  |  |  |  |  |
|  | Formononetin(C_16_H_12_O_4_) | *Astragalus membranaceus（Fisch.）Bge.* | *Fabaceae* | C57BL/6 J mice fed by HFD | (100mg/kg/day, i.g) for 16w | - | Restoring autophagy activity and attenuating lipid accumulation | LC3B-II, PPARα, CPT1α, p-AMPK, Beclin 1, TFEB, LAMP1, ATP6V1A , PGC1α↑  p62 ↓ | body weight, TG, TC, LDL-C, lipid droplets, AST, ALT, AUC of IPGTT↓ | (Wang et al., 2019) |
|  |  |  |  | HepG2 cells stimulated by FFA | (10, 20 µM) for 24h |  |  |  |  |  |
|  | Morin(C_15_H_10_O_7_) | *Morus alba L.* | *Moraceae* | C57BL/6 mice stimulated by intraperitoneal injection of tyloxapol | (60 mg/kg/day, i.p) for 12h | Fenofibrate (100 mg/kg/day, p.o) for 12h | Inhibiting SREBP-1c expression and promoting PPARα expression, ultimately regulating adipogenesis and fatty acid oxidation | TNFα, p-IκB, p-p65, MyD88 , TLR4 , p-ERK1/2, p-p38, p-JNK1/2, SREBP-1c↓  PPARα, p-AMPKα, p-AKT, p-ACC↑ | TG, TC, lipid accumulation, vacuoles formation↓ | (Wu et al., 2019) |
|  |  |  |  | HepG2 cells stimulated by OA | (40, 80 µM) for 24h |  |  |  |  |  |
|  | Dihydromyricetin(C_15_H_12_O_8_) | *Nekemias grossedentata (Hand.-Mazz.) J.Wen & Z.L.Nie* | *Vitaceae* | 129-Sirt3tm1.1Fwa/J(SIRT3KO, SIRT3 knockout) mice fed by HFD  129S1/SvImJ(WT) mice fed by HFD | (300 mg/kg/day, p.o) for 12w | - | Regulating of mitochondrial function and activating of SIRT3 | SIRT3 , SIRT3 , hepatic  ATP content, Nrf1, Tfam, MT-CO1, MT-ATP6, SOD2 activity, pAMPKα/AMPKα, PGC1a, p-AMPK, ERRa↑  mitochondrial complex I and IV activities, SOD2 acetylation↓ | TG, lipid accumulation, body weight, LDL-C, TG, ALT, and NAFLD activity score↓  cell viability↑ | (Zeng et al., 2019) |
|  |  |  |  | Primary hepatocytes from SIRT3KO mice stimulated by PA  Primary hepatocytes from WT mice stimulated by PA  HepG2 cells stimulated by PA | (1, 5, 15, 20, 20, 30 µM) for 24h |  |  |  |  |  |
|  | Baicalein(C_15_H_10_O_5_) | *Scutellaria baicalensis Georgi* | *Lamiaceae* | Kunming mice fed by HFD | (50, 200 mg/kg/day, i,g) 12w | - | Suppressing the cleavage of SREBP1, inhibiting the transcriptional activity of SREBP1, and the synthesis of hepatic fat | ACC, FASN, SREBP1 transcription, abundance of mSREBP, MDA↓  p-SREBP1, abundance of pSREBP1, p-AMPK, p-ACC, T-SOD, T-AOC, GPx↑ | TG, TC, LDL-C, AST, ALT↓  HDL-C↑ | (Sun et al., 2020) |
|  |  |  |  | HepG2 cells stimulated by OA | (1, 5 µM) for 24h |  |  |  |  |  |
|  |  |  |  | Sprague-Dawley rats stimulated by 10% fructose solution | (25, 100 mg/kg/day, i,g) for 5w | - | Promoting fatty acid oxidation | SCD1 , ELOVL6, ChREBP , SREBP1c , ACC , FASN , ACLY , ROS↓  PGC1α, p-AMPK, CPT1α, PPARα↑ | TG, LDL-C, AST, lipid droplet accumulation↓ | (Li et al., 2022b) |
|  | Neohesperidin(C_28_H_34_O_15_) | *Foeniculum piperitum (Ucria) C.Presl* | *Apiaceae* | C57BL/6 mice fed by HFD | (50 mg/kg/day, i.g) for 12w | - | Inhibiting mitochondrial biogenesis through PGC-1α expression | IL-6, IL-1β, Tnf-α, MDA, ROS, Srebf1, Fasn, Scd1, Acc1↓  Cat , Sod1 , Gpx1 , Ucp2 , SOD, CAT, GSH, Pparα, Acaa2, Cpt-1, Pdk4, Acox1, Nrf-1, Tfam, PGC-1α, p-AMPK↑ | body weight, liver weight, adipose tissue weight, ALT, AST, NAS, TC, TG, NEFA, FBG, FINS, HOMA-IR, AUC of OGTT, AUC of ITT↓ | (Wang et al., 2020b) |
|  |  |  |  | HepG2 cells stimulated by PA | (100 µM) for 16h |  |  |  |  |  |
|  | Ugonin J(C_25_H_26_O_6_) | *Helminthostachys zeylanica (L.) Hook.* | *Ophioglossaceae* | C57BL/6 J mice fed by HFD | (15, 30 mg/kg/day p.o) for 12w | - | Upregulating of fatty acid β oxidation and downregulating of de novo lipogenesis | PAI-1, GIP, ratio of pIRS-1(Ser307)/IRS-1, Srebp1c , Fasn , ROS↓  Pparα, Pparδ, pAMPK, pACC, CPT-1, Akt, pFoxO1/FoxO1↑ | body weight, TG, TC, LDL-C, FFA, AUC of IPGTTs, FBG, insulin, GOT, GPT↓ | (Chang et al., 2021) |
|  |  |  |  | HuS-E/2 cells stimulated by PA | (2.5, 5, 10, 20 µM) for 24h |  |  |  |  |  |
|  | Icariin(C_33_H_40_O_15_) | *Epimediumbrevicornu Maxim.* | *Berberidaceae* | C57BL/6J mice fed by HFD | (50, 100, 200 mg/kg/day, p.o) for 3w | Dorsomorphin (15 mg/kg/day, p.o) for 3w | Decreasing blood glucose and improving lipid metabolism | activation of caspase3 and caspase9, SREBP1c , DGAT2 ↓  CPT1 , pACC/ACC, AMPKα1 , PGC1α, GLUT4 ↑ | TC, TG, LDL-C, AUC of ITT, AUC of GTT, AST, ALT, liver apoptotic cells, lipid droplets↓ | (Lin et al., 2021) |
|  | Curcumin(C_21_H_20_O_6_) | *Curcuma longa L.* | *Zingiberaceae* | C57BL/6J mice fed by HFHFD | (50, 150 mg/kg/day, i.g) for 6w | Gemfibrozil (150 mg/kg/day, p.o) for 6w | Restoring citrate homeostasis in hepatic cytoplasm by directly inhibiting the functions and modulating the expressions of SLC13A5 and ACLY | SLC13A5, ACLY, p-mTOR↓  p-AMPK↑ | lipid droplets, TG, TC, NEFA, LDL-C, ALT, AST↓  HDL-C↑ | (Sun et al., 2021) |
|  |  |  |  | primary hepatocytes stimulated by PA and OA | (10 µM) for 24h |  |  |  |  |  |
|  | Naringenin(C_15_H_12_O_5_) | *Citrus maxima (Burm.) Merr.* | *Rutaceae* | Sprague-Dawley rats fed by HFD | (10, 30, 90 mg/kg/day, p.o) for 2w | - | Regulating autophagy and enhancing mitochondrial biogenesis | CaMKKβ, p-AMPK, p-ACC, mitochondrial biogenesis, SIRT1, PGC1α, NRF1, TFAM, p-ULK1, LC3-II/LC3-I↑  p62↓ | TG, TC, ALT, AST, ballooning degeneration, lipid accumulation, total ATP content↓  glucose uptake↑ | (Yang et al., 2021) |
|  |  |  |  | L02 cells stimulated by sodium oleate  Huh-7 cells stimulated by sodium oleate  3T3-L1 adipocytes and C2C12 myotubes | (0.7, 2.2, 6.7, 20 µM) for 24h |  |  |  |  |  |
|  | Acacetin(C_16_H_12_O_5_) | *Saussurea involucrata (Kar. & Kir.) Sch.Bip.* | *Asteraceae* | C57BL/6 mice fed by HFD | (5, 10 mg/kg/day, i.p) for 12w | - | Inhibiting lipogenesis, increasing lipid breakdown, increasing fatty acid β-oxidation, and reducing liver inflammation | Srebp-1c, C/EBPα, C/EBPβ, FAS, TNF-α, IL-6↓  ATGL, HSL, PPAR-α, CPT-1, CPT-2, sirt1, p-AMPK↑ | body weight, the number of lipid vacuoles, the fat vacuole size, TC, TG, NAFLD scores, LDL, GOT, GPT, lipid droplet accumulation↓  HDL↑ | (Liou et al., 2022) |
|  |  |  |  | FL83B cells stimulated by OA | (10, 30 µM) for 24h |  |  |  |  |  |
|  | Xanthohumol(C_21_H_22_O_5_) | *Humulus lupulus L.* | *Cannabaceae* | Wistar–Dawley rats fed by HFD | (20 mg/kg/day, p.o) for 12w | Dorsomophin (0.2 mg/kg/day, p.o) for 12w | Enhancing WAT energy expenditure, suppressing hepatic de novo lipogenesis, and attenuating hepatic inflammation, oxidative stress, fibrosis, and apoptosis | MDA, NF-κB, TNF-α, IL-6, ACC-1 , SREBP1 ↓  GSH, SOD, CAT, Nrf2, p-AMPK↑ | food intake, body weight, AUC of OGTT, AUC of IPITT, final body weights, weights of subcutaneous, epididymal, and peritoneal fats, FBG, fasting insulin, HOMA-IR, leptin, TNF-α, FFAs, TGs, CHOL, FFA, LDL-c, ox-LDL-c↓  HDL-c↑ | (Atteia et al., 2023) |
|  | Quercetin(C_15_H_10_O_7_) | *Ginkgo biloba L.* | *Ginkgoaceae* | C57BL/6J mice fed by MCD | (20, 80 mg/kg/day, p.o) for 4w | Chloroquine(50 mg/kg/day, p.o) for  4w | Promoting mitophagy | TNF-α, IL-6, IL-1β , NLRP3 , ACS, Caspase-1, IL-18, p-NF-κB p65 , ROS↓  AMPK , SIRT1, DHE, PINK1 , Parkin ↑ | hepatic lipid accumulation, ALT, AST, ROS↓ | (Cao et al., 2023) |
|  |  |  |  | HepG2 cells stimulated by OA | (100 µM) |  |  |  |  |  |
|  | Chrysin(C_15_H_10_O_4_) | *Oroxylum indicum (L.) Kurz* | *Bignoniaceae* | C57BL/ mice fed by HFD | (40 mg/kg/day, i,g) for 21w | - | Reducing cholesterol synthesis, and inflammation and improving liver lipid deposition | TNF-α, IL-6, α-SMA , TGF-β1 , SREBP1c , NLRP3, caspase-1 ↓  p-AMPK, P-ACC/ACC↑ | body weight, glucose, TG, TC, LDL, FFA, AST, ALT, HBDH, LDH↓ | (Gao et al., 2023a) |
|  |  |  |  | Wister rats fed by obesogenic Diet | (25, 50, 75 mg/kg/day, p.o) for 4w | - | Inhibiting hepatic lipogenesis, enhancing antioxidants, and boosting mitochondrial | MDA, GSSG, SREBP-1c , mTOR ↓  GSH, GSH/GSSG, AMPK , p-AMPK , LKB1 , PGC-1α , NRF-1 , Tfam ↑ | final weight, weight gain, FBG, insulin, HOMA-IR, TG, TC, LDL-C, ALT, AST, leptin↓  HDL-C, adiponectin↑ | (Oriquat et al., 2023) |
|  | Baicalin(C_21_H_18_O_11_) | *Scutellaria baicalensis Georgi* | *Lamiaceae* | C57BL/6J mice fed by HFD | (100 mg/kg/day, i.g) for 8w | - | Improving lipid accumulation, oxidative imbalance, and inflammation | ROS, MDA, IL-6, TNF-α, SREBP1, ACC, FAS, NF-κB, p-p65/p65, p-IκBα/IκBα↓  SOD, GSH, p-AMPK, Nrf2, HO-1↑ | body weight, food intake, liver weight, liver weight/body weight ratio, AST, ALT, TC, TG↓ | (Gao et al., 2023b) |
|  |  |  |  | AML-12 cells stimulated by PA | (25 µM) for 24h |  |  |  |  |  |
|  | Kaempferol(C_15_H_10_O_6_) | *Ginkgo biloba L.* | *Ginkgoaceae* | C57BLKS/J background db/db mice fed by HFD | (50 mg/kg/day, i.g) | - | Reducing hepatic lipogenesis and promoting fatty acid oxidation | sirt1 , p-AMPK , PGC1α, p-AMPK/AMPK↑  ACC, FASN, SREBP1↓ | hepatic lipid deposition, TG, TC, LDH, body weight, FBG, fasting insulin, HOMA-IR, AST, ALT, NASH score, ballooning score, inflammation score, hepatic lipid droplets, SAT, VAT↓  CHO↑ | (Li et al., 2023a) |
|  |  |  |  | HepG2 cells stimulated by OA | (10^-8^, 10^-7^, 10^-6^ M) for 24h |  |  |  |  |  |
|  | 6-Gingerol(C_17_H_26_O_4_) | *Zingiber officinale Roscoe* | *Zingiberaceae* | C57BL/6J mice fed by HFD | (10, 20 mg/kg/day, i.g) for 5w | - | Alleviating lipid accumulation, inflammation, and oxidative stress | TNF-α, IL-6, ROS, MDA, Srebf1, Acaca, Fasn, Scd1, SREBP1 , ACC , FAS ↓  CAT, GPx, Cpt1α, Pparα, Pgc1α, p-LKB1 , p-AMPK , p-ACC , CPT1α↑ | body weight, AUC of OGTT, glucose, insulin, HOMA-IR, volume and amount of lipid droplets, TG, TC, ALT, AST↓ | (Liu et al., 2023) |
|  |  |  |  | HepG2 cells stimulated by PA | (5, 10, 20, 40 µM) for 24h |  |  |  |  |  |
|  |  |  |  | C57BL/6 N mice fed by HFD | (100 mg/kg/day, i.g) for 4w | - | Inhibiting SREBPs nuclear translocation, resulting in a reduction of TG and TC biosynthesis | TNF-α, IL-6, SREBP1, SREBP2, SREBP1/2 , p-ACC, FASN, HMGCR ↓  p-INSR, p-AKT, p-GSK3β, p-AMPK, LDLR↑ | body weight, liver weight, liver weight-to-body weight ratio, size and weight of eWAT, hepatocellular vacuoles, lipid droplets, AST, ALT, FFA, TG, LDL-c, TC, AUC of IPGTT, AUC of IPITT, fasting insulin, HOMA-IR↓  HDL-c, glycogen↑ | (Xia et al., 2024) |
|  |  |  |  | HepG2 cells stimulated by PA | (100 µM) for 24h |  |  |  |  |  |
|  | Gossypetin(C_15_H_10_O_8_) | *Hibiscus sabdariffa L.* | *Malvaceae* | C57BL/6 mice fed by CDHFD | (20 mg/kg/day, p.o) for 4w | - | Reducing adipogenesis and improving insulin resistance | SREBP1c, Acly, Acox1, Mcp1, cd68, Col1α, Col3α, ROS, cleaved caspase-3↓  Cpt1α, p-AMPK, p-ACC↑ | spleen weight, AST, ALT, NAS, liver FFA↓ | (Oh et al., 2023) |
|  |  |  |  | AML12 stimulated by palmitate | - |  |  |  |  |  |
|  | Oroxin A(C_21_H_20_O_10_) | *Clerodendrum indicum (L.) Kuntze* | *Lamiaceae* | Sprague‒Dawley rats fed by HFD | (50, 200 mg/kg/day, i.g) for 12w | Essentiale Forte N (196.3 mg/kg, p.o)  Atorvastatin (10, 30mg/kg/day, p.o) for 12w | Reducing lipid synthesis | mSREBP1, mSREBP2, ACC, FASN, HMGCR↓  p-AMPK↑ | TG, TC, LDL-C, AST, ALT↓ | (Cai et al., 2024) |
|  |  |  |  | HepG2 cells stimulated by OA | (5, 50 µM) for 24h |  |  |  |  |  |
|  | Rutin(C_27_H_30_O_16_) | *Styphnolobium japonicum (L.) Schott* | *Fabaceae* | db/db mice (fasting blood glucose above 11.1 mmol/l) | (100, 200 mg/kg/day, p.o) for 8w | - | Decreasing fat synthesis, ameliorating inflammation, and oxidative stress | TNF-α, IL-1β, ROS, FASN, SREBP1, SCD1↓  Nrf2, HO-1, NQO1, p-AMPK, p-ACC↑ | cytoplasmic fat vacuoles and edema, glucose uptake ability, TC, TG, AST, ALT↓ | (Liu et al., 2024) |
|  |  |  |  | HepG2 cells stimulated by PA | (20 µM) for 24h |  |  |  |  |  |
|  | Apigenin(C_15_H_10_O_5_) | *Apium graveolens L.* | *Apiaceae* | C57BL/6 mice fed by HFD | (25, 50, 100 mg/kg/day, i.g) for 4w | - | Accelerating downstream lipid degradation | LAMP-2A, CMA, p-AMPK/AMPK, nuclear translocation of Nrf2↑  PLIN2, PLIN3, p-PLIN2/PLIN2↓ | TG, TC, LDL-C, size and number of lipid droplets↓  HDL↑ | (Lu et al., 2024) |
|  |  |  |  | AML12 cells stimulated by FFA | (0.625, 1.25, 2.5, 5, 10 µM) for 72h |  |  |  |  |  |
|  | Dihydromyricetin(C_15_H_12_O_8_) | *Nekemias grossedentata (Hand.-Mazz.) J.Wen & Z.L.Nie* | *Vitaceae* | Sprague Dawley rats fed by HFD | (50, 100, 200 mg/kg/day, p.o) for 6w | Metformin (150 mg/kg/day, p.o) for 6w | Inducing autophagy | G6Pase , PEPCK ↓  GLUT2 , Beclin1 , ATG5 , LC3-II , p-AMPK , PGC-1α , PPARα ↑ | body weight, liver weight, liver index, eWAT weight, TC, TG, LDL-C, AST, ALT, HOMA-IR, AUC of IPGTT, AUC of IPITT↓ | (Yang et al., 2024) |
|  |  |  |  | HepG2 cells stimulated by PA | (5, 10, 20 µM) for 24h |  |  |  |  |  |
|  | Hesperitin(C_28_H_34_O_15_) | *Citrus reticulata Blanco* | *Rutaceae* | Sprague Dawley rats fed by HFD | (100, 200 mg/kg/day, p.o) for 8w | Mdivi-1(1mg/kg/day, p.o) for 8w | Regulating mitochondrial fission/fusion and mediating mitophagy biogenesis | amount of mtDNA copies, SOD, Mfn2 , Opa1 , PINK1 , Parkin , AMPKα , p-AMPKα↑  ROS, MDA, Drp1 , Fis1↓ | body weight, liver weight, liver  index, hepatic steatosis, TG, TC, ALT, AST↓ | (Chen et al., 2025) |
|  | Scopoletin(C_10_H_8_O_4_) | *Datura metel L.* | *Solanaceae* | C57BL/6j mice stimulated by Tyloxapol | (100, 200 mg/kg/day, p.o) for 14h | Simvastatin (20mg/kg/day, p.o) for 14h  14h | Modulating lipogenesis and lipolysis | IL-1β, TNF-α, MDA, SREBP1c, FAS, SCD1, SREBP2, HMGCR, LDLR, lipin1 , TNF-α, F4/80, cas-1, cas-1p10, IL-1β, p65, p-IκBα↓  IL-10, SOD, ATGL, HSL, p-AMPK/AMPK, p-ACC/ACC, HO-1, Keap1, Nrf2 nuclear transcription↑ | ALT, AST, TG, TC, LDL-C, vacuolar response, inflammatory cell infiltration↓  HDL-C↑ | (Zhang et al., 2025) |
|  |  |  |  | L02 cells stimulated by FFA | (10, 20, 40 µM) for 24h |  |  |  |  |  |
|  | Raspberry ketone(C_10_H_12_O_2_) | *Rubus idaeus L.* | *Rosaceae* | Wistar rats fed by HFFD | (55 mg/kg/day, p.o) for 8w | - | Decreasing lipogenesis, and increasing lipolysis and fatty acid oxidation | p-AMPK, PPAR-α, CPT-1 ↑  SREBP-1c, FAS↓ | body weight, liver weight, AST, ALT, TC, TAG, LDL-C↓  HDL-C↑ | (Askar et al., 2023) |
|  | Phloretin(C15H14O5) | *Malus domestica (Suckow) Borkh.*  */* | *Rosaceae* | C57BL/6 mice | (10, 20 mg/kg/day, i.p) for 12w | - | Inhibiting lipogenesis and accumulation, promoting lipolysis and β-oxidation | Srebp-1c, C/EBPβ, FAS, TNF-α↓  ATGL, pHSL, CPT-1, CPT-2, PPARα, p-Sirt-1, p-ACC, p-AMPK↑ | TC, TG, NAFLD scores, LDL, leptin, glucose, insulin, GOT, GPT↓  HDL↑ | (Liou et al., 2020) |
|  |  |  |  | HepG2 cells stimulated by OA | (10, 30 µM) for 24h |  |  |  |  |  |
|  |  |  |  | C57BL/6J mice fed by Western diet | (50, 100, 200 mg/kg/day, p.o) for 16w | - | Augmenting autophagy, alleviating mitochondrial dysfunction and oxidative stress | MMP, ROS, TNF-α, IL-6, MDA, α-SMA, TGF-β, SREBP1c , FAS , SCD1, SQSTM1/p62 ↓  SOD, p-AMPK, p-ACC, FASN, PPARα , CPT1, CPT2, Beclin1, LC3II/I, Atg7, Atg5↑ | TG, TC, body weight, FBG, LW/BW, AST, ALT↓ | (Chhimwal et al., 2022) |
|  |  |  |  | Huh7 cells stimulated by sodium oleate and sodium palmitate | (50, 100, 150 µM) for 24h |  |  |  |  |  |
| **Lignan** | Honokiol(C_18_H_18_O_2_) | *Schisandra chinensis (Turcz.) Baill.* | *Schisandraceae* | C57BL/6J mice fed by choline-deficient HFD | (2.5, 10 mg/kg/day, i.p) for 4w | Resveratrol (10uM) for 4w | Lowering lipid accumulation, maintaining mitochondrial morphology and integrity | SIRT3, p-AMPK↑  acetylated LCAD↓ | lipid accumulation, TG↓ | (Liu et al., 2021b) |
|  |  |  |  | AML12 cells stimulated by PA and OA | (5, 10 µM) for 12h |  |  |  |  |  |
|  |  |  |  | C57BL/6 J fed by HFD | (100 mg/kg/day, i.g) for 12w | - | Promoting fatty acid oxidation, inhibiting lipid accumulation, inflammation, and cell damage | p-AMPKα, p-ACC, p-LKB1↑  mTOR↓ | body weight, liver weight, size and contents of hepatic lipid droplets, fibrosis, inflammatory cell infiltration, AUC of GTT, AUC of ITT, insulin, TC, TG, AST, ALT, NEFA, LW/BW↓ | (Tian et al., 2023) |
|  |  |  |  | L02 cells stimulated by PA and OA | (10 μM) for 18h |  |  |  |  |  |
|  | Schisandrin B(C_23_H_28_O_6_) | *Schisandra chinensis (Turcz.) Baill.* | *Schisandraceae* | C57BL/6J mice fed by HFD | (50 mg/kg/day, i,g) for 5w | 3-MA(4mM)/3-MA(30 mg/kg) for 5w | Promoting autophagy | LC3B-II, p-AMPK, p-ULK1, PI3KC3, Beclin-1, ATG7, ATG5, ATGL, MGL, LIPE (HSL), LIPA, LIPC, LPL, APOA4, GK, CYP7A1, CPT1A, ACOX1, ACADL, ACADM, HMGCS2↑  p62, p-mTOR, p-ULK1, p-p70↓ | lipid droplet, autolysosomes, TC, TG↓ | (Yan et al., 2022) |
|  |  |  |  | HepG2 cells stimulated by FFA | (12.5, 25, 50 ug/ml) for 16h |  |  |  |  |  |
|  | Schisanhenol(C_23_H_30_O_6_) | *Schisandra chinensis (Turcz.) Baill.* | *Schisandraceae* | C57BL/6J mice fed by HFD | (5, 10, 20 mg/kg/day, i,g) for 4w | Pioglitazone hydrochloride (10mg/kg, p.o) for 4w | Inhibiting adipogenesis and promoting fatty acid β-oxidation | p-AMPK, p-ACC1, PPARα, CPT-1↑  SREBP-1c , miR-802, Acc, Scd1, Fas, Dgat1, Dgat2↓ | lipid accumulation, TG, TC, LDC-C↓  HDC-L↑ | (Li et al., 2024) |
|  |  |  |  | HepG2 cells stimulated by FFA | (5, 10, 20 μmol/L) for 24h |  |  |  |  |  |
| **Phenolic acid** | Salvianolic acid A(C_26_H_22_O_10_) | *Salvia miltiorrhiza Bunge* | *Lamiaceae* | C57BL/6J mice fed by HFCD | (20, 40 mg/kg/day, i.p) for 10w | - | Activating autophagy and improving lipotoxicity | p-p38↓  p-AMPK, SIRT1 ↑ | TG, glycerol, FFA, total cholesterol, HDL-C↓ | (Li et al., 2020) |
|  |  |  |  | HepG2 cells stimulated by PA | (25, 50, 100 µM) for 24h |  |  |  |  |  |
|  |  |  |  | ApoE^−/−^ mice fed by Western diet | (10, 20 mg/kg/day, p.o) for 4w | - | Regulating mitochondrial homeostasis, inflammation, fatty acid metabolism, and ferroptosis | α-Sma , Col3a1 , Tgf-β , TNF-α, MCP-1, IL-6, FASN, Srebp1, PPARγ, ACC, CD36, LPL, ROS↓  pAMPK, PPARα , PCK2, ATGL, CPT1α, pGSK3β, IGFBP↑ | lipid droplet vacuoles, TG, AST, ALT↓ | (Zhu et al., 2024) |
|  |  |  |  | AML-12 cells stimulated by FFA | (10, 25, 50 µM) for 24h |  |  |  |  |  |
|  | Hydroxytyrosol(C_8_H_10_O_3_) | *Olea europaea L.* | *Oleaceae* | spotted seabass juveniles  Zebrafish | (200 mg/kg/day, p.o) for 8w | - | Promoting mitophagy | SOD, SDH, CS, PINK1, Mul1, Atg5, LC3B II, P-AMPK↑  ROS, MDA↓ | AST, ALT, TAG, TC↓ | (Dong et al., 2022) |
|  |  |  |  | liver cell line | (50 ug/ml) for 24h |  |  |  |  |  |
|  | Ellagic acid(C_14_H_6_O_8_) | *Terminalia chebula Retz.* | *Combretaceae* | Wistar albino rats fed by 60% fructose-enriched diet | (15 mg/kg/day, p.o) for 4w | Allopurinol (30 mg/kg/day, p.o) for 4w | Inhibiting lipogenesis and reducing oxidative stress | MDA, FAS, aldolase B, ACL, SERBP-1c↓  GSH, p-AMPK, CTRP3↑ | liver weight, insulin, glucose, HOMA-IR, FFA, TG, TC, LDL-C, TC/HDL-C, AST, ALT↓  HDL-C↑ | (Elseweidy et al., 2022) |
|  | Zingerone(C_11_H_14_O_3_) | *Zingiber officinale Roscoe* | *Zingiberaceae* | Wistar rats fed by HFD | (100 mg/kg/day, i.g) for 12w | Dorsomorphin (0.2 mg/kg/day, p.o) for 12w | Ameliorating hepatic steatosis and oxido-inflammatory damage | TNF-α, IL-6, NF-κβ, ROS, MDA, SREBP1c, SREBP2↓  GSH, SOD, Nrf2, Bax, cleaved caspase-3, Bax/Bcl2, p-AMPK↑ | liver weights, FBG, fasting insulin levels, HOMA-IR, TGs, CHOL, LDL-c, ALT, AST↓ | (Mohammed, 2022) |
|  | Gallic acid(C_7_H_6_O_5_) | *Rhus chinensis Mill.* | *Anacardiaceae* | Kunming mice fed by 30% fructose | (100, 200 mg/kg/day, p.o) for 8w | - | Suppressing hepatic de novo lipogenesis, increasing fatty acid β-oxidation, promoting the mitochondrial function/integrity | MDA, ROS, COX-2, TNF-α, IL-6, SREBP-1, FASN, ACC↓  T-SOD, GSH-Px, Nrf2, HO-1, p-AMPKα, CPT1α, ACOX1 ↑ | lipid droplet, TC, TG, AST, ALT, LDL-C↓  HDL-C↑ | (Lu et al., 2023) |
|  |  |  |  | HepG2 cells stimulated by fructose | (0, 25, 50, 100 µM) for 24h |  |  |  |  |  |
|  |  |  |  | C57BL/6 J mice fed by HFD | (50, 100, 200 mg/kg/day, i.g) for 12w | - | Increasing β-oxidation and improving mitochondrial function | Scd1, Fasn, Acaca, FAS, Pparg, ROS↓  p-AMPK, p-ACC, PPARα, CPT1A, OCR↑ | liver weight, body weight, lipid droplet, AST, ALT, TG, TC, LDL↓  HDL↑ | (Zhang et al., 2023b) |
|  |  |  |  | HepG2 cells stimulated by OA/PA | (20 µM) for 24h |  |  |  |  |  |
|  | *P*-Coumaric acid(C_9_H_8_O_3_) | *Angelica sinensis (Oliv.) Diels* | *Apiaceae* | ICR mice fed by Triton WR1339 | (100 mg/kg/day, i,g) for 24h | - | Increasing the activity of hepatic lipolytic enzymes HSL, HTGL, and MGL to promote hepatic lipolysis | HTGL, MGL, HSL, MSS4, p-AMPK, PPARγ, CPT1A, ACSL1↑ | size of lipid droplets, TC, TG↓  lipase activity↑ | (Yuan et al., 2023) |
|  |  |  |  | HepG2 cells stimulated by OA | (10 µg/mL) for 24h |  |  |  |  |  |
|  | Androsin(C_15_H_20_O_8_) | *Picrorhiza kurroa Royle ex Benth.* | *Plantaginaceae* | ApoE ^- / -^ mice fed by D09100310 diet | (10 mg/kg/day, i.p) for 7w | Silymarin (50 mg/kg) for 7w | Reducing lipogenesis and lipid uptake, and increasing autophagy | IL-1α, IL-1β, IL-6, TNFα, α-SMA, NF-κB, IL-1β, SREBP1c, ChREBP, mTOR↓  CYP2E1, PI3K, AMPKα, Beclin 1, LC3B, CPT1α, ACOX1↑ | NAS, AST, ALT, TC, LDL↓ | (Singh et al., 2024) |
|  |  |  |  | HepG2 cells stimulated by PA and OA | (5, 10 µM) for 12h |  |  |  |  |  |
|  | Rosmarinic Acid(C_18_H_16_O_8_) | *Melissa officinalis L.* | *Lamiaceae* | db/db mice fed by MCD diet | (10, 30 mg/kg/day, p.o) for 2w | - | Inhibiting fatty acid synthesis and enhancing fatty acid oxidation | SREBP-1c, FAS, SCD-1, α-SMA, COL1A1↓  PPARα, PGC-1α, CPT-1L, NRF2, SOD1, HO-1, p-AMPK, p-ACC, LKB1↑ | TG, AST, ALT↓ | (Kim et al., 2020) |
|  |  |  |  | HepG2 cells stimulated by PA | (20, 40 µM) for 24h |  |  |  |  |  |
|  | Chicoric acid(C_22_H_18_O_12_) | *Cichorium intybus L.* | *Asteraceae* | C57BL/6 mice fed by HFD | (15, 30 mg/kg/day, i,g) for 9w | - | Protecting hepatocytes from lipid dysregulation, oxidative damage, and inflammation, and NFκB inactivation and shaping gut microbiota | IL-2, IL-6, IL-1β, TNF-α, MDA, ROS, p-IKKα/β, p-IκBα, p-NFκB, keap1↓  SOD, Nrf2, HO-1, SOD1, SOD2 , p-AMPK↑ | body weight, WAT, FBG, TC, TG, LDL-C, GPT-ALT, GOT-AST↓  HDL-C↑ | (Ding et al., 2020) |
|  |  |  |  | HepG2 cells stimulated by PA | (10, 20 µM) for 24h |  |  |  |  |  |
| **Quinone** | Hypericin(C_30_H_16_O_8_) | *Hypericum perforatum L.* | *Hypericaceae* | C57BL/6 J mice fed by HFHS | (0.5, 1, 2 mg/kg/day, i.p) for 30days | - | Promoting lipolysis, inhibiting lipid anabolism, and thereby restricting lipid accumulation in hepatocytes | ROS, cleaved caspase-3, cleaved caspase-9, Bax/Bcl-2, FAS, CD36 , LDLR, SCD1, PPAR-γ↓  p-AMPK, CPT1A, PKACα↑ | lipid droplets, TG, LDL, CRP, FFA, GHbA1c↓  HDL, GLP-1↑ | (Liang et al., 2020) |
|  |  |  |  | L02 cells stimulated by FFA  HepG2 cells stimulated by FFA | (20 nM, 200 nM, 2 µM) for 24h |  |  |  |  |  |
|  | Emodin(C_15_H_10_O_5_) | *Reynoutria multiflora (Thunb.) Moldenke* | *Polygonaceae* | Zebrafish (Danio rerio) of wild-type AB strain fed by Egg yolk powder | 0.25, 0.5 µg/mL for 72h | - | Decreasing lipogenesis and increasing mitochondrial fatty acid β-oxidation | P-AMPKα, PI3K, AKT2, AMPKα, PPARα, ACOX1, CPT-1a, adipoR2↑ | body weight, body length, BMI, TC, TG, NEFA, lipid accumulation, lipid droplets↓ | (Yu et al., 2020) |
|  | Danthron(C_14_H_8_O_4_) | *Rheum rhaponticum L.* | *Polygonaceae* | C57BL/6J mice fed by HFD | (10 mg/kg/day, p.o) for 6w | - | Regulating fatty acid metabolism and mitochondrial biogenesis, reducing the lipid deposition in adipose tissue | SREBP1c, FASN, SCD1, ACC1, CD36, FABP1, FATP1, IL-1β, TNF-α, IL-6↓  ACOX1, CPT1α, LCAD, MCAD, UCP2, SIRT1, PGC1α, AMPKα, AdipoR2, TFam, PPARα, ACOX1 , CPT1α ↑ | body weight, glucose, insulin, GTT, ITT, NEFA, AST, ALT, TG, TC↓ | (Ma et al., 2021) |
|  |  |  |  | HepG2 cells stimulated by insulin(5µg/mL) | (10, 20 µM) |  |  |  |  |  |
|  | Aurantio-obtusin(C_17_H_14_O_7)_ | *Senna tora (L.) Roxb.* | *Fabaceae* | C57BL/6J mice fed by HFSW | (5, 10, 15 mg/kg/day, p.o) for 8w | - | Promoting autophagy flux and alleviating liver steatosis | Ccl2, Tgfb1, Col1a1, FASN, SREBP-1, p62, p-mTOR↓  Acox1, PPARα, LC3-II, LC3-II/LC3-I, Atg5, Atg7, p-AMPK, p-ULK, TFEB↑ | ALT, AST, glucose, weight of WAT, TG, TC, number and size of lipid droplets↓ | (Zhou et al., 2022) |
|  |  |  |  | HepG2 cells stimulated by PA and OA | (12.5, 25, 50 µM) for 24h |  |  |  |  |  |
|  | Rhinacanthin C(C_25_H_30_O_5_) | *Rhinacanthus nasutus (L.) Kurz* | *Acanthaceae* | C57BL/6JNifdc mice fed by HFD | (5, 10, 20 mg/kg/day, p.o) for 12w | Metformin (250 mg/kg/day, p.o) for 12w | Decreasing NAFLD fatty acid β oxidation, oxidative stress, and inflammation | MCP-1, TNF-α, IL-6, SOD, GSH-Px, p-NF-κ B p65, SREBP-1C, FAS, ACC, PPAR-γ , SCD1↓  MDA, PPARα , ACOX1, CPT-1α, p-AMPKα, Sirt1↑ | body weight, liver weight, the liver/body weight ratio, NAS scores, AST, ALT, NEFA, TG, TC, LDL-C, insulin, glucose, leptin, resistin, HOMA-IR, AUC of OGTT, AUC of ITT↓  HDL-C, ADIPOQ, ADIPOR2↑ | (Gong et al., 2023) |
|  | Thymoquinone(C_10_H_12_O_2_) | *Nigella sativa L.* | *Ranunculaceae* | C57BL/6 N mice fed by HFD | (10, 20, 40 mg/kg/day, i,g) for 8w | - | Triggering autophagy | p62, p-mTOR/mTOR, p-ULK1(Ser757) /ULK1↓  LC3-II, p-AMPK/AMPK, p-ULK1(Ser555) /ULK1, Beclin-1↑ | body weight, liver weight, average food intake, AUC of IPGTT, AUC of IPITT, TC, TG, LDL-C↓  HDL-C↑ | (Zhang et al., 2023a) |
|  |  |  |  | HepG2 cells stimulated by FFA | (1, 2, 4 µM) for 24h |  |  |  |  |  |
|  | Shikonin(C_16_H_16_O_5_) | *Lithospermum erythrorhizon Siebold and Zucc (LE)* | *Boraginaceae* | C57BL/6 mice fed by HFD | (30 mg/kg/day, p.o) for 4w | - | Suppressing fatty acid synthesis, enhancing β-oxidation and energy expenditure | p-AMPKα, p-ACC, PPARα, CPT1, PGC-1α, UCP2↑  SREBP1c↓ | body weight, adipocyte size, weight of WAT, TG, TC, hepatic total lipid↓  VCO2↑ | (Gwon et al., 2020) |
|  |  |  |  | Hepa 1-6 cells stimulated by OA | (0.5, 1, 2 uM) for 24h |  |  |  |  |  |
| **Terpenoid** | Betulinic acid(C_30_H_48_O_3_) | *Betula pendula subsp. mandshurica (Regel) Ashburner & McAll.* | *Betulaceae* | C57BL/6 mice fed by HFD | (150 mg/kg/day, p.o) for 11w | - | Suppressing the lipogenesis and degrading the fat | PPARγ, GPAT, DGAT1, DGAT2, FAS, IL-1α, IL-1β, IL-6, TNF-α, SREBP-1c, ApoC2, RBP, FAS, SCD1↓  PGC-1α, UCP-2, CPT1, Acox1, FABP, MCAD, p-AMPK↑ | WAT, BAT, body weight, liver weight, TC, TG, insulin, leptin↓ | (Kim et al., 2019) |
|  |  |  |  | 3T3-L1 adipocytes stimulated by MO | (12.5, 25, 50 ug/ml) for 20h |  |  |  |  |  |
|  | β-Cryptoxanthin(C_40_H_56_O) | *Lycium chinense Mill.* | *Solanaceae* | C57BL/6 J DKO mice fed by HRCD  C57BL/6 J WT mice fed by HRCD | (10 mg/kg/day, p.o) for 24w | - | Suppressing lipogenesis and increasing fatty acid β-oxidation | Fas, Acc2, ACC, SCD1, Hmgcr , Hmgs1, IL-6, IL-4↓  Ppara, Acox1, Cyp7a1, Abcg5, SIRT1, AMPK↑ | large and small fat droplets, TG, TC↓ | (Lim et al., 2019) |
|  | Maslinic acid(C_30_H_48_O_4_) | *Ligustrum lucidum W. T. Aiton* | *Oleaceae* | C57BL/6 mice fed by HFD | (10, 20 mg/kg/day, i.p) for 12w | - | Inhibiting lipogenesis and increasing lipolysis and fatty acid β-oxidation | TNF-α, Srebp-1c, PPAR-γ, C/EBP-β, FAS, C/EBP-β, Srebp-1c↓  ATGL, HSL, Sirt1, CPT-1, CPT-2, p-AMPK, p-ACC↑ | adipocyte size, fat vacuoles, lipid droplets, lipoperoxidation, fatty acid uptake, TC, TG, LDL, ALT, AST, HOMA-IR, NAFLD scores↓  HDL↑ | (Liou et al., 2019a) |
|  |  |  |  | HepG2 cells stimulated by OA | (0-50 µM) for 24h |  |  |  |  |  |
|  | Crocin(C_44_H_64_O_24_) | *Crocus sativus L.* | *Iridaceae* | db/db mice fed by free access to water and a regular chow diet | (20 mg/kg/day, p.o) for 8w | - | Inhibiting lipogenesis and promoting β-oxidation | p-AMPK, PPARα, Acox1, Cpt1, Hmgcs2↑  p-mTOR, SREBP-1c, FAS, SCD1, PPARγ, DGAT↓ | liver weight, AST, ALT, TG, TC, EFA, AUC of OGTT, AUC of IPITT↓ | (Luo et al., 2019) |
|  | Ursolic acid(C_30_H_48_O_3_) | *Prunella vulgaris L.* | *Lamiaceae* | C57BL/6J mice fed by HFD | (60, 80, 100 mg/kg/day, p.o) for 15w | - | Reducing lipogenesis and increasing lipolysis | ACC, FAS, SREBP-1C, SREBP-2, HMGCR↓  CPT-1, CD36, PPARα, ACOX1, AMPK, p-AMPK↑ | average body weight, liver weight, epididymal and perirenal adipose contents, TC, TG, non-HDL-C, size of adipose cells↓ | (Cheng et al., 2020) |
|  |  |  |  | HepG2 stimulated by FFA | (5, 10, 15, 20 µM) for 24h |  |  |  |  |  |
|  | Ganoderic acid A(C_30_H_44_O_7_) | *Ganodermalucidum (Leyss. ex Fr.)* | *Ganodermataceae* | Sprague–Dawley rats fed by HFD | (20, 40 mg/kg/day, p.o) for 2w | Atorvastatin(20 mg/kg/day, p.o) for 2w | Regulating the lipogenesis and inflammation response | IL-1β, TNF-α, IL-6, LXRs, ACC1, FAS, PPAR-γ, p-NF-κB, p-IκBα↓  SREBP-1c, p-AMPK, p-PPAR-α, p-PGC-1α, PPAR-α, PGC-1α↑ | liver weight, weight of liver per body weight, AST, ALT, TBIL, TG, TC, NAS score↓  HDL-C↑ | (Liu et al., 2020) |
|  | Lycopene(C_40_H_56_O) | *Solanum lycopersicum L.* | *Solanaceae* | C57BL/6J mice fed by HFFD | (30 mg/kg/day, p.o) for 10w | - | Enhancing mitochondrial function | p-ACC, PPARα, MMP, SIRT1, PGC1α, p-AMPK, HO-1, NQO-1, Cox5b, Cox7a1, Cox8b↑  FAS, PPARγ↓ | TG, T-CHO, LDL-C↓  HDL-C↑ | (Wang et al., 2020a) |
|  |  |  |  | HepG2 cells stimulated by PA | (100 µM) for 12h |  |  |  |  |  |
|  | Triptolide(C_20_H_24_O_6_) | *Tripterygium wilfordii Hook.f.* | *Celastraceae* | C57BL/6J mice fed by MCD | (50, 100 µg/kg/day, i.p) for 10w | - | Reducing hepatic inflammation and fibrosis | FN, α-SMA, Col VI, IL-1β, IL-6 , TNF-α, TGF-β, MCP-1, Col-1, Col-4, SREBP-1, SCD-1, FAS, ACC1, UCP-2↓  pACC1, pAMPK, AMPK, PPARα, CPT-1α↑ | TC, TG, LW/BW, AST, ALT, DBil, lipid accumulation, NAFLD activity score, fibrosis stage↓ | (Huang et al., 2021) |
|  | Corosolic acid(C_30_H_48_O_4_) | *Crataegus pinnatifida var. pinnatifida* | *Rosaceae* | C57BL/6J mice fed by HFD | (10, 20 mg/kg/day, p.o) for 9w | Silymarin (30 mg/kg/day, p.o) for 9w | Inhibiting lipid biosynthesis and promoting fatty acid β-oxidation | α-SMA, TIMP-1, F4/80, Caspase-1, IL-1β, TNF-α, IL-6, TGF-β1, p65, p-IκBα, SREBP1c, FAS↓  p-Smad2 phosphorylations, pAMPK, pACC↑ | LW/BW, number and size of lipid droplets, AST, ALT, TG, TC↓ | (Liu et al., 2021a) |
|  |  |  |  | HepG2 cells stimulated by FFA  LX2 cells stimulated by TGF-β(10 ng/mL) | (5, 10, 20 µM) for 24h |  |  |  |  |  |
|  | Patchouli alcohol(C_15_H_26_O) | *Pogostemon cablin (Blanco) Benth.* | *Lamiaceae* | C57BL/6J (B6) mice fed by HFD | (20 mg/kg (once every 2 days), i.p) for 8w | - | Regulating inflammation, insulin resistance, and lipid metabolism | SREBP1, SCD1, p-NF-κB, p-IκB, SIRT1↓  p-LKB1, p-AMPK, FAO↑ | AUC of IPGTT, AUC of ITT, HOMA-IR, body weight↓ | (Pyun et al., 2021) |
|  |  |  |  | HepG2 cells stimulated by palmitate  C2C12 cells stimulated by palmitate | (10, 30 µg/ml) for 24g |  |  |  |  |  |
|  | β-patchoulene(C_15_H_24_) | *Pogostemon cablin (Blanco) Benth.* | *Lamiaceae* | Sprague Dawley rats fed by HFD | (10, 20, 40 mg/kg/day, i.p) for 4w | Rosuvastatin(20 mg/kg, p.o) for 4w | Inhibiting lipid synthesis and promoting mitochondrial β-oxidation | FASN, SCD1, SREBP-1c, HMG-CR, MDA↓  p-ACC1/ACC1, GSH-Px, SOD, SIRT1, PGC-1α, PPARα, FGF21, CPT-1a, ACOX1, p-AMPK/AMPK, ↑ | body weight, liver index, AST, ALT, LDL-C, TG, TC↓  HDL-c↑ | (Xu et al., 2021) |
|  |  |  |  | L02 cells stimulated by FFA | (40 µM) for 24h |  |  |  |  |  |
|  | Arjunolic acid(C_30_H_48_O_5_) | *Cyclocarya paliurus (Batalin) Iljinsk.* | *Juglandaceae* | ICR mice fed by HFD | (80 mg/kg/day, i.g) for 8w | - | Inhibiting fatty acid uptake and synthesis, promoting fatty acid β-oxidation, reducing TG synthesis, enhancing TG decomposition and triggering autophagy, and restoring small intestinal barrier | p-AMPK/AMPK, NAD+, NAMPT, p-ACC/ACC, PPARα, CPT1α↑  CD36, FATP2, SREBP-1c, FAS, LC3Ⅱ/LC3Ⅰ, p62, LPS, IL-6, IL-1β, TNF-α↓ | TG, TC, FFA, body weight, liver weight, liver index, lipid droplets, LDL-c, ALT, AST, ALP, HOMA-IR, AUC of OGTT, AUC of ITT, insulin↓ | (Zheng et al., 2021) |
|  |  |  |  | primary hepatocytes stimulated by FFA | (10, 20, 40 uM) for 24h |  |  |  |  |  |
|  | Celastrol(C_29_H_38_O_4_) | *Tripterygium wilfordii Hook.f.* | *Celastraceae*  *Celastraceae* | C57BL/6 N mice fed by HFD | (0.3 mg/kg/day, i.p) for 16days | - | Promoting lipolysis and inhibiting lipid uptake and lipogenesis | Acc, Fasn, Srebp1c, Cd36↓  Acox-1, Cpt-1, SIRT1, p-AMPK↑ | TC, TG, body weight, LW/BW, AUC of GTT, AUC of ITT, AST, ALT↓ | (Fan et al., 2022) |
|  |  |  |  | HepG2 cells stimulated by FFA | (0.3, 0.6, 1.3 µM) for 24h |  |  |  |  |  |
|  |  | *Celastrus orbiculatus Thunb.* |  | C57BL/6J mice fed by HFD | (1, 3, 5 mg/kg/day, i.g) for 12w | Simvastatin (20 mg/kg/day, p.o) for 12w | Enhancing mitochondrial function | Na+-K+-ATP, Ca2+/Mg2+-ATP, SOD, GSH, GSH-Px, FGF21, AMPK, PGC-1α, PPARγ, SIRT3↑  MDA, TNF-α, IL-6↓ | body weight, liver weight, visceral adiposity index, ALT, AST, TG, TC, LDL-C↓ | (Xue et al., 2024) |
|  |  |  |  | HepG2 cells stimulated by NaOL | (0.1, 0.2, 0.4 µg/mL) for 24h |  |  |  |  |  |
|  | Atractylenolide III(C_15_H_20_O_3_) | *Atractylodes macrocephala Koidz.* | *Asteraceae* | C57BL/6J mice fed by HFD | (10 mg/kg/day, i.v) for 4w | - | Inhibiting lipid accumulation and oxidative stress, and promoting fatty acid oxidation | Acc1, Srebp1c, Scd1, ROS, MDA↓  SOD, GSH-Px, AdipoR1, p-LKB1, p-AMPK, SIRT1, SIRT3, Nrf2, CPT1A, PGC1α↑ | lipid accumulation, ballooning degeneration score, NAFLD activity score, body weight, liver weight, ALT, AST, TG, TC, LDL↓ | (Li et al., 2022c) |
|  |  |  |  | HepG2 cells stimulated by FFA | (12.5, 25, 50 µg/ml) for 24h |  |  |  |  |  |
|  | Astaxanthin(C_40_H_52_O_4_) | *Haematococcus Pluvialis* | *Volvocaceae* | ICR mice fed by HFD | (60 mg /kg/day, i.g) for 10w | - | Improving lipid accumulation and oxidative stress | IL-17A , MDA, SREBP-1, ACC, FAS, ROS↓  SOD, CAT, p-AMPK, Nrf2, HO-1↑ | body weight, daily food intake, water consumption, lipid droplet vacuoles, liver index, fat index, ALT, AST, TC, TG, LDL-C↓  HDL-C↑ | (Li et al., 2022d) |
|  |  |  |  | HepG2 cells stimulated by PA and OA | (200 uM) for 24h |  |  |  |  |  |
|  | Limonin(C_26_H_30_O_8_) | *Citrus × aurantium (Lour.) Engl.* | *Rutaceae* | C57BL/6 mice fed by HFD | (50 mg/kg/day, p.o) for 9w | - | Inhibiting lipid accumulation | p-AMPK, p-ACC, ADP/ATP↑  SREBP1c, SREBP2, Fasn, Acc1, Scd1, Hmgcr, Hmgcs↓ | body weight, liver weight, ALP, ALT, AST, amount of epididymal and subcutaneous white adipose tissue↓ | (Wang et al., 2022) |
|  |  |  |  | AML12 cells stimulated by PA  HepG2 cells stimulated by PA | (50, 100 µM) for 16h |  |  |  |  |  |
|  | Nootkatone(C_15_H_22_O) | *Alpinia oxyphylla Miq.* | *Asteraceae* | Mice fed by HFD | (25, 50 mg/kg/day, p.o) for 12w | - | Regulating hepatocyte glucose and lipid metabolism | AMPK, p-AMPKα, p-ACC↑  p-ERK1/2, p-p38, p-JNK, IL-1β, IL-18, TNFα, IL-6, IL-1β, IL-18, TNFα, IL-6↓ | body weight, FBG, TG, CHO, AUC of OGTT, AST, ALT, liver weight, LW/BW↓  HDL-c↑ | (Yong et al., 2022) |
|  |  |  |  | L02 cells stimulated by PA and OA | (5, 10, 20, 40 µM) for 24h |  |  |  |  |  |
|  | Ginsenoside CK(C_36_H_62_O_8_) | *Panax ginseng C.A.Mey.* | *Araliaceae* | Kunming mice fed by dietary fructose | (30, 60 mg/kg/day, i.g) for 8w | Silibinin (60 mg/kg/day, p.o) for 8w | Inhibiting lipid synthesis and accelerating lipolysis and fatty acid oxidation | IL-6, TNFα, MDA, SREBP-1c, FASN↓  GSH-Px, SOD, p-ACC1/ACC1, ATGL, Sirt1, PPARα, CPT1α, p-AMPK/AMPK, p-LKB1, p-AMPK, p-LKB1↑ | TG, TC, food intake, body weight, liver weight, fat tissue weight, AST, ALT, LDL-C, glucose, steatosis score ↓  HDL-C↑ | (Zhang et al., 2022) |
|  |  |  |  | HepG2 cells stimulated by PA | (1, 2, 5, 10, 15, 20, 30 µM) for 24h |  |  |  |  |  |
|  | Acetyl-11-keto-beta-boswellic acid(C_32_H_48_O_5_) | *Boswellia serrata Roxb* | *Burseraceae* | Wistar rats fed by HFrD | (100 mg/kg/day, p.o) for 2w | - | Preventing the decline of lipid degradation metabolism and inhibiting liver inflammation | MCP-1, CAPS-1, NLRP3, CASP-1, ASC, IL-6, TNF-α , TGF-β, INF-Υ↓  the activity of mMDH, the activity of mCS, AMPK-α1, p-AMPK-α1↑ | TG, TC, final weights, increased weights, insulin, leptin, LDL, AST, ALT↓  adiponectin, HDL↑ | (Kachouei et al., 2023) |
|  | Protopanaxadiol(C_30_H_52_O_3_) | *Panax ginseng C. A. Mey.* | *Araliaceae* | C57BL/6 J mice | (10, 20 mg/kg/day, i.g) for 8w | - | Inhibiting lipogenesis and promoting fatty acid oxidation | SIRT1, p-AMPK, p-ACC, PGC-1α, CPT-1, ACOX1↑  FASN, SREBP-1↓ | LW/BW, ALT, AST, LDH, TC, TG, LDL-C↓  HDL-C↑ | (Li et al., 2023b) |
|  |  |  |  | HepG2 and AML2 cells | (1.25, 2.5, 5 µM) for 12h |  |  |  |  |  |
|  | Asperuloside(C_18_H_22_O_11_) | *Gardenia jasminoides J. Ellis* | *Rubiaceae* | C57BL/6J mice fed by HFD | (20 mg/kg/day, i.g) for 10w | - | Improving lipid deposition | IL-1β, TNF-α, IL-18, Srebf1, Fasn, SREBP-1c, FAS, Nlrp3, pro-IL-1β , NLRP3, ASC, Caspase-1↓  p-AMPK↑ | body weight, TC, TG, AST, ALT, histopathological score, liver coefficient, LDL-C↓  HDL-C↑ | (Shen et al., 2023b) |
|  |  |  |  | HepG2 cells stimulated by PA | (10, 20, 40 uM) for 24h |  |  |  |  |  |
|  | Gypenoside XIII(C_41_H_70_O_12_) | *Gynostemma pentaphyllum (Thunb.) Makino* | *Cucurbitaceae* | C57BL/6 mice fed by MCD | (10 mg/kg/day, i.p) for 4w | - | Inhibiting lipid synthesis in hepatocytes | ROS, SREBP-1c, FAS, α-SMA, F4/80 , COX-2, IL-1β↓  p-ACC, ATGL, CPT-1, CPT-2, SIRT1, p-AMPK↑ | lipid accumulation, GOT↓ | (Cheng et al., 2024) |
|  |  |  |  | HepG2 cells stimulated by OA | (5, 10, 15, 20 µM) for 24h |  |  |  |  |  |
|  | Alisol B(C_30_H_48_O_4_) | *Alisma plantago-aquatica L.* | *Alismataceae* | C57BL/6 J mice fed by WD | (25, 50 mg/kg/day, i,g) for 10w | Lovastatin(10 mg/kg/day, p.o) for 10w | Inhibiting the production of cholesterol and fatty acids | n-SREBP-1, n-SREBP-2, FASN, HMGCR, ACC1, PCSK9, MVK, p-mTOR, p-p70S6K↓  AMPK, p-ACC↑ | TC, TG, LDL-c, AST, ALT, liver weight↓  HDL-c, ↑ | (Gao et al., 2024) |
|  |  |  |  | HepG2 cells stimulated by OA  HL7702 cells stimulated by OA | (2.5, 5, 10, 15 µg/ml) for 24h |  |  |  |  |  |
|  | β-Caryophyllene(C_15_H_24_) | *Myristica fragrans Houtt.* | *Myristicaceae* | C57BL/6J mice fed by HFD | (0.3% w/w β-Caryophyllene, p.o) for 12w | - | Inhibiting lipogenesis to alleviate hepatic steatosis, weight gain, and metabolic disorders | p-AMPK, p-AMPK/AMPK, p-ACC1/ACC1, p-SREBP-1c/SREBP-1↑  ACC1, SREBP-1c↓ | body weight, WAT weight, insulin, leptin, TC, TG, glucose, hepatocellular vacuolar degeneration, lipid droplet accumulation, AST, ALT↓ | (Kamikubo et al., 2024) |
|  | Ginsenoside Rg5(C_42_H_70_O_12_) | *Panax ginseng C. A. Mey.* | *Araliaceae* | C57BL/6J mice fed by HFD | (50, 100 mg/kg/day, p.o) for 12w | - | Enhancing energy metabolism and modulating autophagy | MDA, p-mTOR↓  SOD, CAT, GSH-Px, LKB1, p-AMPK↑ | body weight, AUC of OGTT, AUC of ITT, lipid deposition, TG, TC, LDL-C, AST, ALT↓ | (Shi et al., 2024) |
|  | Platycodon D(C_36_H_58_O_12_) | *Platycodon grandiflorus (Jacq.) A. DC.* | *Campanulaceae* | C57/BL mice fed by HFD | (10 mg/kg/day, p.o) for 8w | - | Improving glucose and lipid metabolism | CD36, Srebp1c, Chrebp, IL-1β, IL-6, NPC1L1, Apob, Mttp, Abca1↓  p-AMPK↑ | body weight, AUC of GTT, AUC of ITT, TG, TC, AST, ALT, insulin, HOMA-IR↓ | (Tang et al., 2024) |
|  |  |  |  | Caco2 cells | (4 µM) for 12h |  |  |  |  |  |
|  | Morroniside(C_17_H_26_O_11_) | *Cornus officinalis Siebold & Zucc.* | *Cornaceae* | C57BL/6 mice fed by HFFD | (10, 20 mg/kg/day, i.g) for 8w | Fenofibrate (20 mg/kg/day, p.o) for 8w | Promoting hepatic lipid droplet degradation by enhancing lipophagy and fatty acid oxidation | p-AMPKα, ATG12-ATG5, LC3II, PLIN2, PPARα, CPT1α, T-SOD↑  p62, ROS, MDA, NLRP3, ASC, Caspase-1 p20, IL-18, IL-1, TNF-α, IL-6, IL-1β | TG, TC, AST, ALT, LDL-C, NAS score↓  HDL-C↑ | (Zhang et al., 2024) |
|  |  |  |  | HepG2 cells stimulated by PA | (0, 25, 50 µM) for 24h |  |  |  |  |  |
|  | Ginkgolide C(C_20_H_24_O_11_) | *Ginkgo biloba* | *Ginkgoaceae* | C57BL/6 mice fed by Western diet | (25 mg/kg /qw, i.p) for 16w | - | Improving steatosis and insulin resistance, reducing the inflammatory response | Ccl2, Cxcl1, IL-1β, TNF-α, Col1a1, CTGF, TGFβ, TIMP-1, SREBP-1c, FOXO1, p-p65↓  p-AMPK, PGC1a, p-IRS1↑ | AST, ALT, NAFLD activity score, TG, TC, body weight, FBG, serum insulin, HOMA-IR, AUC of GTT, AUC of ITT↓ | (Xie et al., 2024) |
| **Glycoside** | Glucoraphanin(C_12_H_23_NO_10_S_3_) | *Brassica oleracea var. italica Plenck* | *Brassicaceae* | C57BL/6 J mice fed by HFD | (0.3% glucoraphanin, p.o) for 11w | - | Reducing the expression levels of fatty acid synthesis genes and pro-inflammatory chemokine genes | Srebp-1c, Acc, Scd1, JNK, CCL2, CCR2↓  Pparα, Cpt1a, p-AMPKα/AMPK↑ | blood glucose, plasma insulin, TG, AST, ALT↓ | (Promsuwan et al., 2024) |
|  | Catalpol(C_15_H_22_O_10_) | *Rehmannia glutinosa (Gaertn.) Libosch. ex DC.* | *Rehmannia* | ob/ob mice fed by HFD | (100 mg/kg/day, i.g) for 4w | - | Inducing autophagy, attenuating hepatic steatosis and lipotoxicity | ACC1α, FAS, SQSTM1/p62↓  PPARα, CPT1, LC3-II, p-AMPK↑ | body weight, LW/BW, TG, TC, AST, ALT↓ | (Ren et al., 2019) |
|  |  |  |  | HepG2 cells stimulated by PA | (10 µg/mL) for 24h |  |  |  |  |  |
|  |  |  |  | C57BL/6J mice fed by HFD | (100, 200, 400 mg/kg/day, i.g) for 18w | Atorvastatin calcium (30 mg/kg/day, p.o) for 18w | Suppressing lipogenesis, inducing fatty acid β-oxidation | p-AMPK, p-ACC, PPARα, CPT1, ACOX1↑  SREBP-1c, FAS↓ | lipid droplet, TG, TC, AST, ALT, liver weight, body weight, lipid droplet accumulation↓ | (Tian et al., 2020) |
|  |  |  |  | HepG2 cells stimulated by PA | (100, 200, 400µM) for 24h |  |  |  |  |  |
|  | Aucubin(C_15_H_22_O_9_) | *Aucuba japonica Thunb.* | *Garryaceae* | C57/BL6 mice stimulated by tyloxapol | (10, 20, 40 mg/kg, i.p) | Fenofibrate (100 mg/kg/day, p.o) | Decreasing the lipid accumulation and oxidative damage | MMP-9, MCP-1, apoC-III, ICAM-1, VCAM-1, MPO, TNF-α, IL-1β, IL-6↓  SOD, Nrf2, HO-1, PPARα, PPARγ, p-ACC, p-AMPKα, p-AMPKβ, p-AKT↑ | TC, TG, LDL-C, VLDL, body weight, liver weight, liver index↓  HDL-C↑ | (Shen et al., 2019) |
|  |  |  |  | 3T3-L1 cells stimulated by 1μmol/L insulin | (35, 70, 140 µg/mL) for 24h |  |  |  |  |  |
|  | Cordycepin(C_10_H_13_N_5_O_3_) | *Cordyceps militaris (L.ex Fr.) Link.* | *Clavicipitaceae* | C57BL/6J mice fed by HFD | (50 mg/kg, i.g) for 8w | - | Regulating lipid formation and promoting glucose uptake and fatty acid oxidation | MIP2, mKC, VCAM1, TNF-α, lL-6 , IL-1β, MCP1, SREBP1-c, ACC, SCD-1, LXRα, CD36↓  p-AMPK, AMPK, CPT-1, PPARα↑ | body weight, lipid droplets, TC, TG, LDL-C, AST, ALT↓  HDL-C↑ | (Gong et al., 2021) |
|  |  |  |  | C57BL/6J mice fed by HFD | (100, 200 mg/kg/day, p.o) for 8w | - | Ameliorating hepatic steatosis, inflammation, and fibrosis | Fasn, Scd1, Accα, Pparγ, IL6, TNFα, Ccl2, Ccl5, Cxcl10, p-IKKβ, p-p65, Hmgcr, Srebp1, Scd1, Cd36, Fabp1↓  IKBα↑ | lipid accumulation, TG, TC, ALT, AST↓ | (Lan et al., 2021) |
|  |  |  |  | LO2 cells stimulated by OA and PA | (50, 100 µM) for 12h |  |  |  |  |  |
|  | Salidroside(C_14_H_20_O_7_) | *Rhodiola rosea L.* | *Crassulaceae* | C57BL/6J mice fed by HFD | (100, 200 mg/kg/day, i.g) for 8w | - | Regulating fatty acid metabolism and inflammation | Acaca, Fasn, Scd1, Il6, Tnf, Il1b, NF-κB, G6pc, Pck1, Cd36, Fabp1, Fasn, Pparg, Scd1, Srebf1, PPARγ, Col1a1, Ctgf, α-Sma, Tgfb1↓  p-IRS1, p-AKT, p-GSK3β, AMPKα, p-AMPKα↑ | lipid droplet accumulation, TG, white adipose weight, WW/BW, FBG, insulin, AUC of GTT, AUC of ITT, liver weight, LW/BW, TG, TC, LDL-C, ALT, AST↓ | (Hu et al., 2021) |
|  |  |  |  | Primary hepatocytes stimulated by PO | (50, 100 µM) for 12h |  |  |  |  |  |
|  |  |  |  | Wistar rats fed by HFD | (300 mg/kg/day, i.g) for 12w | - | Improving peripheral insulin sensitivity and suppressing hepatic DNL, oxidative stress, and inflammation | MDA, IL-6, TNF-α, nuclear levels of NF-κB, SREBP1, SREBP2, FAS , HMGCoAR, miR-21↓  nuclear DNA binding activity of Nrf2, GSH, SOD, p-AMPK, PPARa↑ | body fat deposits, liver fat deposits, AUC of IPGTT, AUC of PITT, HOMA-IR, FBG, insulin, CHOL, TGs, FFA, LDL-c↓  HDL-c↑ | (Almohawes et al., 2024) |
|  | Mangiferin(C_19_H_18_O_11_) | *Mangifera indica L.* | *Anacardiaceae* | C57BL/6J mice fed by HFD | (25, 50, 100 mg/kg/day, p.o) for 12w | - | Decreasing lipid accumulation, and inflammation and Improving insulin resistance | p-AMPKα, p-ACC↑  NLRP3, caspase-1, caspase-1(p10), IL-1β↓ | body weight, FBG, TG, LDL-C, CHO-C↓  HDL-C, liver weight, liver index, AST, ALT, AUC of OGTT, AUC of ITT↑ | (Zhang et al., 2021) |
|  |  |  |  | HepG2 cells stimulated by PA-BSA | (20, 50, 100 µM) for 24h |  |  |  |  |  |
|  | Hesperidin(C_28_H_34_O_15_) | *Citrus reticulata Blanco* | *Rutaceae* | C57BL/6 mice fed by HFD | (150, 300 mg/kg/day, i.g) for 12w | - | Inhibiting fatty acid synthesis to improve hepatic lipid accumulation | ACC, FAS, SREBP-1C↓  pAMPK/AMPK↑ | TC, TG, LDL, liver index, ALT, AST, lipid droplet↓  HDL↑ | (Chen et al., 2022) |
|  |  |  |  | HepG2 cells stimulated by OA | (50, 100 µmol/L) for 24h |  |  |  |  |  |
|  | Geniposide(C_17_H_24_O_10_) | *Gardenia jasminoides J.Ellis* | *Rubiaceae* | ICR mice fed by HFD | (50 mg/kg/day, p.o) for 12w | Metformin (140 mg/kg, p.o) for 12w | Reducing hepatic lipid accumulation | LPS, TNF-α, IL-6, TLR4, p-IKK-β, p-IκB-α↓  p-AMPK, p-ACC, CPT1A, p-ULK1, p-LC3B II↑ | body weight, AST, ALT, TC, TG↓ | (Yi et al., 2023) |
|  |  |  |  | Nrf2−/− C57BL/6 mice stimulated by tyloxapol | (50, 75, 100 mg/kg/day, i.p) for 18h | Fenofibrate (100 mg/kg, p.o) for 18h | Inducing oxidative stress and inflammation | Nrf2, PPARα, PPARγ, HO-1, p-ACC, p-AKT, p-AMPKα, p-AMPKβ, p-GSK 3β↑  PI3K, P-mTORC, P-S6K, P-S6, SREBP-1c , HMGB1, MMP-9, ApoC3, VCAM-1, ICAM-1, MCP-1, TNF-α, IL-1β, IL-6↓ | lipid droplets, TC, TG, LDL, VLDL↓  HDL↑ | (Shen et al., 2020) |
|  |  |  |  | HepG2 cells stimulated by OA and PA | (65, 130, 260 µmol/L) for 24h |  |  |  |  |  |
| **Stilbenes** | Resveratrol(C_14_H_12_O_3_) | *Reynoutria japonica Houtt* | *Polygonaceae* | Sprague–Dawley rats fed by HFD | (100 mg/kg/day, p.o) for 8w | - | Improving lipid metabolism and redox homeostasis | PPARα, CPT-1, SOD, CAT, p-AMPK, p-ACC, p-PKA↑  SREBP-1c, FAS, ROS, mtROS, MDA↓ | body weight, liver index, TG, TC, LDL-C, glucose, insulin, ALT, AST, lipid droplets↓ | (Huang et al., 2020) |
|  |  |  |  | HepG2 cells stimulated by PA | (40 µmol/L) for 24h |  |  |  |  |  |
|  | Polydatin(C_20_H_22_O_8_) | *Reynoutria japonica Houtt* | *Polygonaceae* | C57BL/6J mice fed by 30% fructose | (50 mg/kg/day, p.o) for 10w | Resveratrol (50 mg/kg, p.o) for 10w | Reducing the oxidative stress, anti-oxidant, anti-inflammatory | p-AMPK, CPT-1α, PPAR-α, PPAR-γ, p-ACC, , p-AKT, SOD↑  MDA↓ | TC, TG, ITT, Insulin, FBG, HbA1c, FFA, LDL-C↓ | (Zhao et al., 2022) |
|  | Pterostilbene(C_16_H_16_O_3_) | *Pterocarpus indicus Willd.* | *Fabaceae* | WT C57BL/6 mice fed by tyloxapol | (30, 45, 60 mg/kg/day, i.p) for 24h | Fenofibrate  (100 mg/kg, p.o) for 24h | Improving intracellular energy metabolism disorders and inhibiting DNL | PPARα, HO-1, p-ACC, p-AKT, p-AMPKα, p-AMPKβ, PI, ATG7, ATG16L1, ATG12-ATG5, ATG5, Beclin-1, SOD, GSH↑  p-mTORC1, p-S6K, S6, SREBP-1c , HMGB1, ROS, MPO, MMP-9, VCAM-1, MDA↓ | TG, TC, VLDL, LDL↓  HDL↑ | (Shen et al., 2023a) |
|  |  |  |  | HepG2 cells stimulated by OA and PA | (12.5, 25 µg/ml) for 12h |  |  |  |  |  |
| **Others** | L-theanine(C_7_H_14_N_2_O_3_) | *Camellia sinensis (L.) Kuntze* | *Theaceae* | C57BL/6J mice fed by HFD | (150, 300, 600 mg/kg/day, i.g) for 16w | Saline(300mg/kg/day, p.o) for 16w | Inhibiting fatty acid synthesis and promoting fatty acid β-oxidation | FASN, ACC1, PPARγ, SREBP-1c-N, Srebf1↓  PPARα, CPT1A, p-CaMKKβ↑ | TG, ALT, AST, body weight, lipid droplets, NAFLD activity score, average size of inguinal and epididymal adipocytes↓  HDL-C↑ | (Liang et al., 2022a) |
|  |  |  |  | HepG2 cells stimulated by OA | (1, 2, 4 mM) for 24h |  |  |  |  |  |
|  | Plantamajoside(C_29_H_36_O_16_) | *Plantago asiatica L.* | *Plantaginaceae* | Sprague Dawley rats fed by HFD | (20, 40, 80 mg/kg/day, i.p) for 4w | - | Improving immune dysregulation and abnormal hepatic lipid metabolism | IL-1β, IL-6, TNF-α, SREBF1, PPARγ, FABP1↓  CPT1α, p-AMPK/AMPK, Nrf2, HO-1↑ | body weight, lipid vacuoles, ALT, AST, LDL-C, TG, TC↓  HDL-C↑ | (Wu et al., 2023) |
|  | Tartaric acid(C_4_H_6_O_6_) | *Rumex acetosa L.* | *Polygonaceae* | C57BL/6 J mice fed by WD | (50, 100, 200 mg/kg/day, i.g) for 12w | - | Suppressing fatty acid synthesis | p-AMPK, p-ACC↑  SREBF1C, FATP4, FABP2, PANK2, ABHD15↓ | body weight, AUC of IPGTT, AUC of IPITT, TG, TC, AST, ALT, lipid droplets, NAS↓ | (Pei et al., 2024) |
|  |  |  |  | HepG2 cells stimulated by OA | (1, 2, 3, 4 mM) for 12h |  |  |  |  |  |
|  | Sulforaphane(C_6_H_11_NOS_2_) | *Raphanus sativus L.* | *Brassicaceae* | C57BL/6J mice fed by HFD | (10 mg/kg/day, i.p) for 3w | - | Reducing hepatic de novo lipogenesis and inhibiting excessive lipid accumulation | p-AMPK↑  SREBP1c, FAS, cleaved caspase 3, ROS↓ | body weight, TG, TC, ALT, blood glucose↓ | (Li et al., 2022a) |
|  |  |  |  | HepG2 cells stimulated by PA | (5, 10 µM) for 24h |  |  |  |  |  |
|  |  |  |  | albino Wistar rats fed by HFD | (10 mg/kg/day, p.o) for 4w | - | Regulating ER stress, lipid metabolism, and glucose homeostasis | MDA, IL-1β, leptin, resistin, FAS, ATF4, IRE-1α, PERK, sXBP-1, CHOP↓  SOD, CAT, GSH, PPAR-α, AMPK, p-PI3K, p-AKT↑ | body weight, ALT, AST, ALP, TG, TC, LDL-c, FFA, HOMA-IR, glucose, insulin↓  HDL-c↑ | (Mansour et al., 2022) |
|  | Atractylodin(C_13_H_10_O) | *Atractylodes Lancea (Thunb.) DC.* | *Asteraceae* | C57BL/6N mice fed by HFD | (5, 10 mg/kg/day, p.o) for 4w | - | Downregulating the lipogenic genes and improving insulin resistance | Gclc, Angptl, Pdk4, Lipin1, p-AMPK, p-ACC↑  Gale, Fasn, SREBF1, SREBP1c, Scd2, Mogat1, FAS, Dgat2, FASN, ACACA↓ | weight of white adipose tissues, TG, TC, AST, ALT, fat droplets, liver weight, FBG, insulin, HOMA-IR, AUC of GTT, AUC of ITT↓ | (Song et al., 2024) |
|  |  |  |  | HepG2 cells stimulated by LXR agonist, T0901317 (T090) | (5, 10, 20, 40, 80 µM) for 24h |  |  |  |  |  |
|  | Antrodan(C_19_H_22_O_4_) | *Antrodia cinnamomea T. T. Chang & W. N. Chou* | *Basidiomycetes* | C57BL/6 mice fed by HFD(high-fat and high-fructose diet) | (20, 40 mg/kg/day, p.o) for 45days | Orlistat (10 mg/kg/day ,p.o) for 45 days | Inhibiting lipid biosynthesis and promoting obese- and steatohepatitis-associated insulin resistance | p-AMPK, Sirt1↑  PPARγ, SREBP-1c↓ | liver weight, body weight, LW/BW, plasma levels of malondialdehyde, TC,  TG, GOT, GPT, uric acid, glucose, insulin, leptin, adiponectin↓ | (Chyau et al., 2020) |
|  | Salsalate(C_14_H_10_O_5_) | */* |  | C57BL/6J mice fed by HFD | (300 mg/kg/day, i.p) for 1w | - | Regulating adipocyte differentiation and lipogenesis | p-AMPK↑  IL-1β, IL-6, TNF-α, PPARγ, C/EBPα, SREBP-1c, aP2↓ | body weight, FBG, TG↓ | (Li et al., 2021) |
|  |  |  |  | HEK293T cells and HepG2 cells | - |  |  |  |  |  |
|  | Diosgenin(C_27_H_42_O_3_) | *Dioscorea polystachya Turcz.* | *Dioscoreaceae* | Sprague Dawley rats fed by HFD and streptozotocin | (10, 20 mg/kg/day, i,g) for 8w | Metformin (300 mg/kg/day, p.o) for 8w | Suppressing DNL and enhancing FAO | Bax, CytC, Apaf-1, cleaved caspase 9, cleaved caspase 3, SREBP1c, FASN, CD36, p-CHOP, ATF4, Caspase 12, PERK, p-PERK, p-EIF2α, IRE1, p-IRE1, XBP1, MDA, MCP-1, IL-1β, TNF-α, Bax, CytC, Apaf-1, DRP1, p-DRP1↓  PPARα, CPT1, p-ACC, AMPK, p-AMPK, SOD, CAT, GPx, IL-10, MFN1, MFN2↑ | TG, TC, FFA, LDL, LW/BW↓  HDL↑ | (Zhong et al., 2022) |
|  | Folic acid(C_19_H_19_N_7_O_6_) | *Morus alba L.* | *Moraceae* | Sprague-Dawley rats fed by high-fructose diet (HF) | (40 mg/kg day, p.o) for 8w | - | Inhibiting liver lipid synthesis and accumulation | p-LKB1, p-AMPK, p-ACC↑ | FBG, GPT, TC, TG, number and size of fat droplets, hepatic SAM↓ | (Kim and Min, 2020) |

MCD, Methionine-Choline Deficient Diet; PA, Palmitic acid; HFHSD, High Fat High Sugar Diet; HFD, High-Fat Diet; OA, Oleic acid; CDAHFD, Choline-deficient, L-amino acid-defined, high-fat diet; FFA, Free fatty acids; HFHFD, high-fat plus high-fructose diet; CDHFD, Choline-deficient high-fat diet; HFHS, High-Fat High-Sugar; HFSW, High-fat diet and glucose-fructose water; HFFD, High-Fat and High-Fructose Diet; PO, Palmitic acid/oleic acid; PA-BSA, Palmitic acid coupled with bovine serum albumin; WD, Western diet; TG, Triglycerides; TC, Total cholesterol; AST, Aspartate aminotransferase; ALT, Alanine aminotransferase; LDL-C, Low-density lipoprotein cholesterol; HDL-C, High-density lipoprotein cholesterol; GLU, Glucose; GTT, Glucose tolerance test; OGTT, Oral glucose tolerance test; IPGTT, Intraperitoneal glucose tolerance test; ITT, Insulin tolerance test; liver weight/body weight; NEFA, Non-esterified fatty acids; FFA, Free fatty acids; HOMA-IR, Homeostatic model assessment of insulin resistance; ALP, Alkaline phosphatase; GOT, glutamic oxaloacetic transaminase; GPT, glutamic pyruvic transaminase; FBG, Fasting blood glucose; WAT, White adipose tissue; BMI, body mass index; VLDL, Very low-density lipoprotein cholesterol;HbA1c, Glycated Hemoglobin A1c; NAS, NAFLD activity score; SAT, Subcutaneous adipose tissue; VAT, Visceral adipose tissue; GSP Glycosylated serum protein; WW/BW, White adipose weight/body weight ratio**.**

**References：**

Almohawes, Z.N., El-Kott, A., Morsy, K., Shati, A.A., El-Kenawy, A.E., Khalifa, H.S., et al. (2024). Salidroside inhibits insulin resistance and hepatic steatosis by downregulating miR-21 and subsequent activation of AMPK and upregulation of PPARα in the liver and muscles of high fat diet-fed rats. *Archives of Physiology and Biochemistry* 130(3)**,** 257-274. doi: 10.1080/13813455.2021.2024578.

Askar, M.E., Ali, S.I., Younis, N.N., Shaheen, M.A., and Zaher, M.E. (2023). Raspberry ketone ameliorates nonalcoholic fatty liver disease in rats by activating the AMPK pathway. *Eur J Pharmacol* 957**,** 176001. doi: 10.1016/j.ejphar.2023.176001.

Atteia, H.H., AlFaris, N.A., Alshammari, G.M., Alamri, E., Ahmed, S.F., Albalwi, R., et al. (2023). The Hepatic Antisteatosis Effect of Xanthohumol in High-Fat Diet-Fed Rats Entails Activation of AMPK as a Possible Protective Mechanism. *Foods* 12(23). doi: 10.3390/foods12234214.

Cai, T.Q., Xu, X.X., Dong, L., Liang, S.F., Xin, M.L., Wang, T.Q., et al. (2024). Oroxin A from Oroxylum indicum improves disordered lipid metabolism by inhibiting SREBPs in oleic acid-induced HepG2 cells and high-fat diet-fed non-insulin-resistant rats. *Heliyon* 10(7). doi: 10.1016/j.heliyon.2024.e29168.

Cao, P., Wang, Y., Zhang, C., Sullivan, M.A., Chen, W., Jing, X., et al. (2023). Quercetin ameliorates nonalcoholic fatty liver disease (NAFLD) via the promotion of AMPK-mediated hepatic mitophagy. *J Nutr Biochem* 120**,** 109414. doi: 10.1016/j.jnutbio.2023.109414.

Chang, T.C., Chiou, W.C., Lai, W.H., Huang, H.C., Huang, Y.L., Liu, H.K., et al. (2021). Ugonin J improves metabolic disorder and ameliorates nonalcoholic fatty liver disease by regulating the AMPK/AKT signaling pathway. *Pharmacol Res* 163**,** 105298. doi: 10.1016/j.phrs.2020.105298.

Chen, C., Liu, X.C., and Deng, B. (2024). Protective Effects of Berberine on Nonalcoholic Fatty Liver Disease in db/db Mice via AMPK/SIRT1 Pathway Activation. *Curr Med Sci* 44(5)**,** 902-911. doi: 10.1007/s11596-024-2914-y.

Chen, H., Nie, T., Zhang, P.L., Ma, J., and Shan, A.S. (2022). Hesperidin attenuates hepatic lipid accumulation in mice fed high-fat diet and oleic acid induced HepG2 via AMPK activation. *Life Sciences* 296. doi: 10.1016/j.lfs.2022.120428.

Chen, S.W., Lu, H.F., Yin, G.L., Zhang, X., Meng, D.C., Yu, W.F., et al. (2025). Hesperitin prevents non-alcoholic steatohepatitis by modulating mitochondrial dynamics and mitophagy via the AMPKα-Drp1/ PINK1-Parkin signaling pathway. *Biochimica Et Biophysica Acta-Molecular and Cell Biology of Lipids* 1870(1). doi: 10.1016/j.bbalip.2024.159570.

Chen, W., Zhang, X., Xu, M., Jiang, L., Zhou, M., Liu, W., et al. (2021). Betaine prevented high-fat diet-induced NAFLD by regulating the FGF10/AMPK signaling pathway in ApoE(-/-) mice. *Eur J Nutr* 60(3)**,** 1655-1668. doi: 10.1007/s00394-020-02362-6.

Cheng, J., Liu, Y., Liu, Y.J., Liu, D., Liu, Y., Guo, Y.T., et al. (2020). Ursolic acid alleviates lipid accumulation by activating the AMPK signaling pathway<i>in vivo</i>and<i>in vitro</i>. *Journal of Food Science* 85(11)**,** 3998-4008. doi: 10.1111/1750-3841.15475.

Cheng, S.C., Liou, C.J., Wu, Y.X., Yeh, K.W., Chen, L.C., and Huang, W.C. (2024). Gypenoside XIII regulates lipid metabolism in HepG2 hepatocytes and ameliorates nonalcoholic steatohepatitis in mice. *Kaohsiung J Med Sci* 40(3)**,** 280-290. doi: 10.1002/kjm2.12795.

Chhimwal, J., Goel, A., Sukapaka, M., Patial, V., and Padwad, Y. (2022). Phloretin mitigates oxidative injury, inflammation, and fibrogenic responses via restoration of autophagic flux in <i>in vitro</i> and preclinical models of NAFLD. *Journal of Nutritional Biochemistry* 107. doi: 10.1016/j.jnutbio.2022.109062.

Chyau, C.C., Wang, H.F., Zhang, W.J., Chen, C.C., Huang, S.H., Chang, C.C., et al. (2020). Antrodan Alleviates High-Fat and High-Fructose Diet-Induced Fatty Liver Disease in C57BL/6 Mice Model via AMPK/Sirt1/SREBP-1c/PPARγ Pathway. *Int J Mol Sci* 21(1). doi: 10.3390/ijms21010360.

Ding, X.Q., Jian, T.Y., Li, J.W., Lv, H., Tong, B., Li, J., et al. (2020). Chicoric Acid Ameliorates Nonalcoholic Fatty Liver Disease via the AMPK/Nrf2/NF<i>κ</i>B Signaling Pathway and Restores Gut Microbiota in High-Fat-Diet-Fed Mice. *Oxidative Medicine and Cellular Longevity* 2020. doi: 10.1155/2020/9734560.

Dong, Y., Yu, M., Wu, Y., Xia, T., Wang, L., Song, K., et al. (2022). Hydroxytyrosol Promotes the Mitochondrial Function through Activating Mitophagy. *Antioxidants (Basel)* 11(5). doi: 10.3390/antiox11050893.

Elseweidy, M.M., Elesawy, A.E., Sobh, M.S., and Elnagar, G.M. (2022). Ellagic acid ameliorates high fructose-induced hyperuricemia and non-alcoholic fatty liver in Wistar rats: Focusing on the role of C1q/tumor necrosis factor-related protein-3 and ATP citrate lyase. *Life Sci* 305**,** 120751. doi: 10.1016/j.lfs.2022.120751.

Fan, N., Zhao, J., Zhao, W., Zhang, X., Song, Q., Shen, Y., et al. (2022). Celastrol-loaded lactosylated albumin nanoparticles attenuate hepatic steatosis in non-alcoholic fatty liver disease. *J Control Release* 347**,** 44-54. doi: 10.1016/j.jconrel.2022.04.034.

Fan, W., Pan, M., Zheng, C., Shen, H., Pi, D., Song, Q., et al. (2024). Leonurine Inhibits Hepatic Lipid Synthesis to Ameliorate NAFLD via the ADRA1a/AMPK/SCD1 Axis. *Int J Mol Sci* 25(19). doi: 10.3390/ijms251910855.

Gao, C.Y., Zhang, H., Nie, L.L., He, K.W., Li, P.M., Wang, X.X., et al. (2023a). Chrysin prevents inflammation-coinciding liver steatosis via AMPK signalling. *Journal of Pharmacy and Pharmacology* 75(8)**,** 1086-1099. doi: 10.1093/jpp/rgad041.

Gao, G., Zhao, J., Ding, J., Liu, S., Shen, Y., Liu, C., et al. (2024). Alisol B regulates AMPK/mTOR/SREBPs via directly targeting VDAC1 to alleviate hyperlipidemia. *Phytomedicine* 128**,** 155313. doi: 10.1016/j.phymed.2023.155313.

Gao, Y., Liu, J., Hao, Z., Sun, N., Guo, J., Zheng, X., et al. (2023b). Baicalin ameliorates high fat diet-induced nonalcoholic fatty liver disease in mice via adenosine monophosphate-activated protein kinase-mediated regulation of SREBP1/Nrf2/NF-κB signaling pathways. *Phytother Res* 37(6)**,** 2405-2418. doi: 10.1002/ptr.7762.

Gong, X., Li, T., Wan, R., and Sha, L. (2021). Cordycepin attenuates high-fat diet-induced non-alcoholic fatty liver disease via down-regulation of lipid metabolism and inflammatory responses. *Int Immunopharmacol* 91**,** 107173. doi: 10.1016/j.intimp.2020.107173.

Gong, Z.Q., Han, S., Li, C.L., Meng, T.X., Huo, Y., Liu, X.F., et al. (2023). <i>Rhinacanthin C</i> Ameliorates Insulin Resistance and Lipid Accumulation in NAFLD Mice via the AMPK/SIRT1 and SREBP-1c/FAS/ACC Signaling Pathways. *Evidence-Based Complementary and Alternative Medicine* 2023. doi: 10.1155/2023/6603522.

Gwon, S.Y., Ahn, J., Jung, C.H., Moon, B., and Ha, T.Y. (2020). Shikonin Attenuates Hepatic Steatosis by Enhancing Beta Oxidation and Energy Expenditure via AMPK Activation. *Nutrients* 12(4). doi: 10.3390/nu12041133.

Hu, M., Zhang, D., Xu, H., Zhang, Y., Shi, H., Huang, X., et al. (2021). Salidroside Activates the AMP-Activated Protein Kinase Pathway to Suppress Nonalcoholic Steatohepatitis in Mice. *Hepatology* 74(6)**,** 3056-3073. doi: 10.1002/hep.32066.

Huang, R., Guo, F., Li, Y., Liang, Y., Li, G., Fu, P., et al. (2021). Activation of AMPK by triptolide alleviates nonalcoholic fatty liver disease by improving hepatic lipid metabolism, inflammation and fibrosis. *Phytomedicine* 92**,** 153739. doi: 10.1016/j.phymed.2021.153739.

Huang, Y.J., Lang, H.D., Chen, K., Zhang, Y., Gao, Y.X., Ran, L., et al. (2020). Resveratrol protects against nonalcoholic fatty liver disease by improving lipid metabolism and redox homeostasis via the PPARα pathway. *Applied Physiology Nutrition and Metabolism* 45(3)**,** 227-239. doi: 10.1139/apnm-2019-0057.

Inamdar, S., Joshi, A., Malik, S., Boppana, R., and Ghaskadbi, S. (2019). Vitexin alleviates non-alcoholic fatty liver disease by activating AMPK in high fat diet fed mice. *Biochem Biophys Res Commun* 519(1)**,** 106-112. doi: 10.1016/j.bbrc.2019.08.139.

Kachouei, R.A., Doagoo, A., Jalilzadeh, M., Khatami, S.H., Rajaei, S., Jahan-Abad, A.J., et al. (2023). Acetyl-11-Keto-Beta-Boswellic Acid Has Therapeutic Benefits for NAFLD Rat Models That Were Given a High Fructose Diet by Ameliorating Hepatic Inflammation and Lipid Metabolism. *Inflammation* 46(5)**,** 1966-1980. doi: 10.1007/s10753-023-01853-y.

Kamikubo, R., Yoshida, H., Fushimi, T., Kamei, Y., and Akagawa, M. (2024). β-Caryophyllene, a dietary phytocannabinoid, alleviates high-fat diet-induced hepatic steatosis in mice via AMPK activation. *Biosci Biotechnol Biochem* 88(12)**,** 1465-1471. doi: 10.1093/bbb/zbae129.

Kim, H., and Min, H. (2020). Folic acid supplementation prevents high fructose-induced non-alcoholic fatty liver disease by activating the AMPK and LKB1 signaling pathways. *Nutr Res Pract* 14(4)**,** 309-321. doi: 10.4162/nrp.2020.14.4.309.

Kim, K.D., Jung, H.Y., Ryu, H.G., Kim, B., Jeon, J., Yoo, H.Y., et al. (2019). Betulinic acid inhibits high-fat diet-induced obesity and improves energy balance by activating AMPK. *Nutr Metab Cardiovasc Dis* 29(4)**,** 409-420. doi: 10.1016/j.numecd.2018.12.001.

Kim, M., Yoo, G., Randy, A., Son, Y.J., Hong, C.R., Kim, S.M., et al. (2020). Lemon Balm and Its Constituent, Rosmarinic Acid, Alleviate Liver Damage in an Animal Model of Nonalcoholic Steatohepatitis. *Nutrients* 12(4). doi: 10.3390/nu12041166.

Lan, T., Yu, Y., Zhang, J., Li, H., Weng, Q., Jiang, S., et al. (2021). Cordycepin Ameliorates Nonalcoholic Steatohepatitis by Activation of the AMP-Activated Protein Kinase Signaling Pathway. *Hepatology* 74(2)**,** 686-703. doi: 10.1002/hep.31749.

Li, B., Xiao, Q., Zhao, H., Zhang, J., Yang, C., Zou, Y., et al. (2024). Schisanhenol ameliorates non-alcoholic fatty liver disease via inhibiting miR-802 activation of AMPK-mediated modulation of hepatic lipid metabolism. *Acta Pharm Sin B* 14(9)**,** 3949-3963. doi: 10.1016/j.apsb.2024.05.014.

Li, J., Chen, C., Zhang, W., Bi, J., Yang, G., and Li, E. (2021). Salsalate reverses metabolic disorders in a mouse model of non-alcoholic fatty liver disease through AMPK activation and caspase-6 activity inhibition. *Basic Clin Pharmacol Toxicol* 128(3)**,** 394-409. doi: 10.1111/bcpt.13535.

Li, J.W., Xie, S.Y., and Teng, W.D. (2022a). Sulforaphane Attenuates Nonalcoholic Fatty Liver Disease by Inhibiting Hepatic Steatosis and Apoptosis. *Nutrients* 14(1). doi: 10.3390/nu14010076.

Li, N., Yin, L., Shang, J.M., Liang, M.D., Liu, Z.Y., Yang, H.G., et al. (2023a). Kaempferol attenuates nonalcoholic fatty liver disease in type 2 diabetic mice via the Sirt1/AMPK signaling pathway. *Biomedicine & Pharmacotherapy* 165. doi: 10.1016/j.biopha.2023.115113.

Li, P., Zhang, R.Y., Wang, M., Chen, Y.W., Chen, Z.W., Ke, X.M., et al. (2022b). Baicalein Prevents Fructose-Induced Hepatic Steatosis in Rats: In the Regulation of Fatty Acid De Novo Synthesis, Fatty Acid Elongation and Fatty Acid Oxidation. *Frontiers in Pharmacology* 13. doi: 10.3389/fphar.2022.917329.

Li, Q., Tan, J.X., He, Y., Bai, F., Li, S.W., Hou, Y.W., et al. (2022c). Atractylenolide III ameliorates Non-Alcoholic Fatty Liver Disease by activating Hepatic Adiponectin Receptor 1-Mediated AMPK Pathway. *Int J Biol Sci* 18(4)**,** 1594-1611. doi: 10.7150/ijbs.68873.

Li, S., Qian, Q., Ying, N., Lai, J., Feng, L., Zheng, S., et al. (2020). Activation of the AMPK-SIRT1 pathway contributes to protective effects of Salvianolic acid A against lipotoxicity in hepatocytes and NAFLD in mice. *Front Pharmacol* 11**,** 560905. doi: 10.3389/fphar.2020.560905.

Li, Y.H., Liu, J.X., Ye, B.J., Cui, Y.Y., Geng, R.Q., Liu, S., et al. (2022d). Astaxanthin Alleviates Nonalcoholic Fatty Liver Disease by Regulating the Intestinal Flora and Targeting the AMPK/Nrf2 Signal Axis. *Journal of Agricultural and Food Chemistry* 70(34)**,** 10620-10634. doi: 10.1021/acs.jafc.2c04476.

Li, Y.P., Liu, Y., Chen, Z.W., Tang, K.Y., Yang, L.L., Jiang, Y.W., et al. (2023b). Protopanaxadiol ameliorates NAFLD by regulating hepatocyte lipid metabolism through AMPK/SIRT1 signaling pathway. *Biomedicine & Pharmacotherapy* 160. doi: 10.1016/j.biopha.2023.114319.

Liang, C., Li, Y., Bai, M., Huang, Y., Yang, H., Liu, L., et al. (2020). Hypericin attenuates nonalcoholic fatty liver disease and abnormal lipid metabolism via the PKA-mediated AMPK signaling pathway in vitro and in vivo. *Pharmacol Res* 153**,** 104657. doi: 10.1016/j.phrs.2020.104657.

Liang, J., Gu, L., Liu, X., Yan, X., Bi, X., Fan, X., et al. (2022a). L-theanine prevents progression of nonalcoholic hepatic steatosis by regulating hepatocyte lipid metabolic pathways via the CaMKKβ-AMPK signaling pathway. *Nutr Metab (Lond)* 19(1)**,** 29. doi: 10.1186/s12986-022-00664-6.

Liang, L., Ye, S., Jiang, R., Zhou, X., Zhou, J., and Meng, S. (2022b). Liensinine alleviates high fat diet (HFD)-induced non-alcoholic fatty liver disease (NAFLD) through suppressing oxidative stress and inflammation via regulating TAK1/AMPK signaling. *Int Immunopharmacol* 104**,** 108306. doi: 10.1016/j.intimp.2021.108306.

Lim, J.Y., Liu, C., Hu, K.Q., Smith, D.E., Wu, D.Y., Lamon-Fava, S., et al. (2019). Dietary β-Cryptoxanthin Inhibits High-Refined Carbohydrate Diet-Induced Fatty Liver via Differential Protective Mechanisms Depending on Carotenoid Cleavage Enzymes in Male Mice. *JOURNAL OF NUTRITION* 149(9)**,** 1553-1564. doi: 10.1093/jn/nxz106.

Lin, W., Jin, Y., Hu, X., Huang, E., and Zhu, Q. (2021). AMPK/PGC-1α/GLUT4-Mediated Effect of Icariin on Hyperlipidemia-Induced Non-Alcoholic Fatty Liver Disease and Lipid Metabolism Disorder in Mice. *Biochemistry (Mosc)* 86(11)**,** 1407-1417. doi: 10.1134/s0006297921110055.

Liou, C.J., Dai, Y.W., Wang, C.L., Fang, L.W., and Huang, W.C. (2019a). Maslinic acid protects against obesity-induced nonalcoholic fatty liver disease in mice through regulation of the Sirt1/AMPK signaling pathway. *Faseb j* 33(11)**,** 11791-11803. doi: 10.1096/fj.201900413RRR.

Liou, C.J., Lee, Y.K., Ting, N.C., Chen, Y.L., Shen, S.C., Wu, S.J., et al. (2019b). Protective Effects of Licochalcone A Ameliorates Obesity and Non-Alcoholic Fatty Liver Disease Via Promotion of the Sirt-1/AMPK Pathway in Mice Fed a High-Fat Diet. *Cells* 8(5). doi: 10.3390/cells8050447.

Liou, C.J., Wu, S.J., Shen, S.C., Chen, L.C., Chen, Y.L., and Huang, W.C. (2020). Phloretin ameliorates hepatic steatosis through regulation of lipogenesis and Sirt1/AMPK signaling in obese mice. *Cell Biosci* 10**,** 114. doi: 10.1186/s13578-020-00477-1.

Liou, C.J., Wu, S.J., Shen, S.C., Chen, L.C., Chen, Y.L., and Huang, W.C. (2022). Acacetin Protects against Non-Alcoholic Fatty Liver Disease by Regulating Lipid Accumulation and Inflammation in Mice. *Int J Mol Sci* 23(9). doi: 10.3390/ijms23094687.

Liu, F., Shi, K., Dong, J., Jin, Z., Wu, Y., Cai, Y., et al. (2020). Ganoderic acid A attenuates high-fat-diet-induced liver injury in rats by regulating the lipid oxidation and liver inflammation. *Arch Pharm Res* 43(7)**,** 744-754. doi: 10.1007/s12272-020-01256-9.

Liu, G., Cui, Z., Gao, X., Liu, H., Wang, L., Gong, J., et al. (2021a). Corosolic acid ameliorates non-alcoholic steatohepatitis induced by high-fat diet and carbon tetrachloride by regulating TGF-β1/Smad2, NF-κB, and AMPK signaling pathways. *Phytother Res* 35(9)**,** 5214-5226. doi: 10.1002/ptr.7195.

Liu, J., Zhang, T., Zhu, J., Ruan, S., Li, R., Guo, B., et al. (2021b). Honokiol attenuates lipotoxicity in hepatocytes via activating SIRT3-AMPK mediated lipophagy. *Chin Med* 16(1)**,** 115. doi: 10.1186/s13020-021-00528-w.

Liu, Y., Sun, Z., Dong, R., Liu, P., Zhang, X., Li, Y., et al. (2024). Rutin ameliorated lipid metabolism dysfunction of diabetic NAFLD via AMPK/SREBP1 pathway. *Phytomedicine* 126**,** 155437. doi: 10.1016/j.phymed.2024.155437.

Liu, Y.Z., Li, D., Wang, S., Peng, Z., Tan, Q., He, Q.F., et al. (2023). 6-Gingerol Ameliorates Hepatic Steatosis, Inflammation and Oxidative Stress in High-Fat Diet-Fed Mice through Activating LKB1/AMPK Signaling. *International Journal of Molecular Sciences* 24(7). doi: 10.3390/ijms24076285.

Lu, J., Zhou, H.J., Hu, J.P., Zhang, R.R., Meng, Z.Q., and Guan, S. (2024). Apigenin Reduces Lipid Droplet Accumulation in Hepatocytes by Enhancing Chaperone-Mediated Autophagy via AMPK. *Journal of Agricultural and Food Chemistry*. doi: 10.1021/acs.jafc.4c08430.

Lu, Y., Zhang, C., Song, Y., Chen, L., Chen, X., Zheng, G., et al. (2023). Gallic acid impairs fructose-driven de novo lipogenesis and ameliorates hepatic steatosis via AMPK-dependent suppression of SREBP-1/ACC/FASN cascade. *Eur J Pharmacol* 940**,** 175457. doi: 10.1016/j.ejphar.2022.175457.

Luo, L., Fang, K., Dan, X., and Gu, M. (2019). Crocin ameliorates hepatic steatosis through activation of AMPK signaling in db/db mice. *Lipids Health Dis* 18(1)**,** 11. doi: 10.1186/s12944-018-0955-6.

Ma, C., Wang, Z., Xia, R., Wei, L., Zhang, C., Zhang, J., et al. (2021). Danthron ameliorates obesity and MAFLD through activating the interplay between PPARα/RXRα heterodimer and adiponectin receptor 2. *Biomed Pharmacother* 137**,** 111344. doi: 10.1016/j.biopha.2021.111344.

Mansour, S.Z., Moustafa, E.M., and Moawed, F.S.M. (2022). Modulation of endoplasmic reticulum stress via sulforaphane-mediated AMPK upregulation against nonalcoholic fatty liver disease in rats. *Cell Stress & Chaperones* 27(5)**,** 499-511. doi: 10.1007/s12192-022-01286-w.

Mohammed, H.M. (2022). Zingerone ameliorates non-alcoholic fatty liver disease in rats by activating AMPK. *J Food Biochem* 46(7)**,** e14149. doi: 10.1111/jfbc.14149.

Oh, E., Lee, J., Cho, S., Kim, S.W., Won, K., Shin, W.S., et al. (2023). Gossypetin Prevents the Progression of Nonalcoholic Steatohepatitis by Regulating Oxidative Stress and AMP-Activated Protein Kinase. *Mol Pharmacol* 104(5)**,** 214-229. doi: 10.1124/molpharm.123.000675.

Oriquat, G., Masoud, I.M., Kamel, M.A., Aboudeya, H.M., Bakir, M.B., and Shaker, S.A. (2023). The Anti-Obesity and Anti-Steatotic Effects of Chrysin in a Rat Model of Obesity Mediated through Modulating the Hepatic AMPK/mTOR/lipogenesis Pathways. *Molecules* 28(4). doi: 10.3390/molecules28041734.

Pei, Y.F., He, Y., Wang, X.F., Xie, C., Li, L., Sun, Q.Y., et al. (2024). Tartaric acid ameliorates experimental non-alcoholic fatty liver disease by activating the AMP-activated protein kinase signaling pathway. *EUROPEAN JOURNAL OF PHARMACOLOGY* 975. doi: 10.1016/j.ejphar.2024.176668.

Promsuwan, S., Sawamoto, K., Xu, L., Nagashimada, M., Nagata, N., and Takiyama, Y. (2024). A natural Nrf2 activator glucoraphanin improves hepatic steatosis in high-fat diet-induced obese male mice associated with AMPK activation. *Diabetol Int* 15(1)**,** 86-98. doi: 10.1007/s13340-023-00658-6.

Pyun, D., Kim, T.J., Park, S.Y., Lee, H.J., Abd El-Aty, A.M., Jeong, J.H., et al. (2021). Patchouli alcohol ameliorates skeletal muscle insulin resistance and NAFLD via AMPK/SIRT1-mediated suppression of inflammation. *MOLECULAR AND CELLULAR ENDOCRINOLOGY* 538. doi: 10.1016/j.mce.2021.111464.

Ren, H., Wang, D., Zhang, L., Kang, X., Li, Y., Zhou, X., et al. (2019). Catalpol induces autophagy and attenuates liver steatosis in ob/ob and high-fat diet-induced obese mice. *Aging (Albany NY)* 11(21)**,** 9461-9477. doi: 10.18632/aging.102396.

Seo, J., Kwon, D., Kim, S.H., Byun, M.R., Lee, Y.H., and Jung, Y.S. (2024). Role of autophagy in betaine-promoted hepatoprotection against non-alcoholic fatty liver disease in mice. *Current Research in Food Science* 8. doi: 10.1016/j.crfs.2023.100663.

Sharma, A., Anand, S.K., Singh, N., Dwarkanath, A., Dwivedi, U.N., and Kakkar, P. (2021). Berbamine induced activation of the SIRT1/LKB1/AMPK signaling axis attenuates the development of hepatic steatosis in high-fat diet-induced NAFLD rats. *Food Funct* 12(2)**,** 892-909. doi: 10.1039/d0fo02501a.

Shen, B., Feng, H., Cheng, J., Li, Z., Jin, M., Zhao, L., et al. (2020). Geniposide alleviates non-alcohol fatty liver disease via regulating Nrf2/AMPK/mTOR signalling pathways. *J Cell Mol Med* 24(9)**,** 5097-5108. doi: 10.1111/jcmm.15139.

Shen, B., Wang, Y., Cheng, J., Peng, Y., Zhang, Q., Li, Z., et al. (2023a). Pterostilbene alleviated NAFLD via AMPK/mTOR signaling pathways and autophagy by promoting Nrf2. *Phytomedicine* 109**,** 154561. doi: 10.1016/j.phymed.2022.154561.

Shen, B., Zhao, C., Wang, Y., Peng, Y., Cheng, J., Li, Z., et al. (2019). Aucubin inhibited lipid accumulation and oxidative stress via Nrf2/HO-1 and AMPK signalling pathways. *J Cell Mol Med* 23(6)**,** 4063-4075. doi: 10.1111/jcmm.14293.

Shen, Q., Chen, Y., Shi, J., Pei, C., Chen, S., Huang, S., et al. (2023b). Asperuloside alleviates lipid accumulation and inflammation in HFD-induced NAFLD via AMPK signaling pathway and NLRP3 inflammasome. *Eur J Pharmacol* 942**,** 175504. doi: 10.1016/j.ejphar.2023.175504.

Shi, Y., Chen, J., Qu, D., Sun, Q., Yu, Y., Zhang, H., et al. (2024). Ginsenoside Rg(5) Activates the LKB1/AMPK/mTOR Signaling Pathway and Modifies the Gut Microbiota to Alleviate Nonalcoholic Fatty Liver Disease Induced by a High-Fat Diet. *Nutrients* 16(6). doi: 10.3390/nu16060842.

Singh, A., Ansari, A., Gupta, J., Singh, H., Jagavelu, K., and Sashidhara, K.V. (2024). Androsin alleviates non-alcoholic fatty liver disease by activating autophagy and attenuating <i>de novo</i> lipogenesis. *Phytomedicine* 129. doi: 10.1016/j.phymed.2024.155702.

Song, G.Y., Kim, S.M., Back, S., Yang, S.B., and Yang, Y.M. (2024). Atractylodes Lancea and Its Constituent, Atractylodin, Ameliorates Metabolic Dysfunction-Associated Steatotic Liver Disease via AMPK Activation. *Biomol Ther (Seoul)*. doi: 10.4062/biomolther.2024.083.

Sun, Q.S., Niu, Q., Guo, Y.T., Zhuang, Y., Li, X.N., Liu, J., et al. (2021). Regulation on Citrate Influx and Metabolism through Inhibiting SLC13A5 and ACLY: A Novel Mechanism Mediating the Therapeutic Effects of Curcumin on NAFLD. *Journal of Agricultural and Food Chemistry* 69(31)**,** 8714-8725. doi: 10.1021/acs.jafc.1c03105.

Sun, W., Liu, P., Wang, T., Wang, X., Zheng, W., and Li, J. (2020). Baicalein reduces hepatic fat accumulation by activating AMPK in oleic acid-induced HepG2 cells and high-fat diet-induced non-insulin-resistant mice. *Food Funct* 11(1)**,** 711-721. doi: 10.1039/c9fo02237f.

Tang, X.K., Yang, Y., Peng, W.X., Xu, M.P., Fan, Q.T., Li, F., et al. (2024). Platycodon D reduces obesity and non-alcoholic fatty liver disease induced by a high-fat diet through inhibiting intestinal fat absorption. *Frontiers in Pharmacology* 15. doi: 10.3389/fphar.2024.1412453.

Tian, R., Yang, J., Wang, X., Liu, S., Dong, R., Wang, Z., et al. (2023). Honokiol acts as an AMPK complex agonist therapeutic in non-alcoholic fatty liver disease and metabolic syndrome. *Chin Med* 18(1)**,** 30. doi: 10.1186/s13020-023-00729-5.

Tian, X., Ru, Q., Xiong, Q., Wen, R., and Chen, Y. (2020). Catalpol Attenuates Hepatic Steatosis by Regulating Lipid Metabolism via AMP-Activated Protein Kinase Activation. *Biomed Res Int* 2020**,** 6708061. doi: 10.1155/2020/6708061.

Wang, J., Geng, T.H., Zou, Q.H., Yang, N.R., Zhao, W.Y., Li, Y.T., et al. (2020a). Lycopene prevents lipid accumulation in hepatocytes by stimulating PPARα and improving mitochondrial function. *Journal of Functional Foods* 67. doi: 10.1016/j.jff.2020.103857.

Wang, M.Y., Zhang, S.S., An, M.F., Xia, Y.F., Fan, M.S., Sun, Z.R., et al. (2023). Neferine ameliorates nonalcoholic steatohepatitis through regulating AMPK pathway. *Phytomedicine* 114. doi: 10.1016/j.phymed.2023.154798.

Wang, S.W., Lan, T., Chen, H.F., Sheng, H., Xu, C.Y., Xu, L.F., et al. (2022). Limonin, an AMPK Activator, Inhibits Hepatic Lipid Accumulation in High Fat Diet Fed Mice. *Frontiers in Pharmacology* 13. doi: 10.3389/fphar.2022.833705.

Wang, S.W., Sheng, H., Bai, Y.F., Weng, Y.Y., Fan, X.Y., Lou, L.J., et al. (2020b). Neohesperidin enhances PGC-1α-mediated mitochondrial biogenesis and alleviates hepatic steatosis in high fat diet fed mice. *Nutrition & Diabetes* 10(1). doi: 10.1038/s41387-020-00130-3.

Wang, Y., Zhao, H.L., Li, X., Wang, Q., Yan, M.H., Zhang, H.J., et al. (2019). Formononetin alleviates hepatic steatosis by facilitating TFEB-mediated lysosome biogenesis and lipophagy. *Journal of Nutritional Biochemistry* 73. doi: 10.1016/j.jnutbio.2019.07.005.

Wu, J.M., Zhaori, G., Mei, L., Ren, X.M., Laga, A.T., and Deligen, B. (2023). Plantamajoside modulates immune dysregulation and hepatic lipid metabolism in rats with nonalcoholic fatty liver disease via AMPK/Nrf2 elevation. *Kaohsiung J Med Sci* 39(8)**,** 801-810. doi: 10.1002/kjm2.12712.

Wu, L., Wang, Y., Chi, G., Shen, B., Tian, Y., Li, Z., et al. (2019). Morin reduces inflammatory responses and alleviates lipid accumulation in hepatocytes. *J Cell Physiol* 234(11)**,** 19785-19798. doi: 10.1002/jcp.28578.

Wu, S.J., Huang, W.C., Yu, M.C., Chen, Y.L., Shen, S.C., Yeh, K.W., et al. (2021). Tomatidine ameliorates obesity-induced nonalcoholic fatty liver disease in mice. *JOURNAL OF NUTRITIONAL BIOCHEMISTRY* 91. doi: 10.1016/j.jnutbio.2021.108602.

Xia, Q.S., Chen, Y., Li, J.B., Huang, Z.Y., Fang, K., Hu, M.L., et al. (2024). 6-Gingerol regulates triglyceride and cholesterol biosynthesis to improve hepatic steatosis in MAFLD by activating the AMPK-SREBPs signaling pathway. *Biomedicine & Pharmacotherapy* 170. doi: 10.1016/j.biopha.2023.116060.

Xie, Y., Wei, L.Y., Guo, J.S., Jiang, Q.S., Xiang, Y., Lin, Y., et al. (2024). Ginkgolide C attenuated Western diet-induced non-alcoholic fatty liver disease via increasing AMPK activation. *INFLAMMATION*. doi: 10.1007/s10753-024-02086-3.

Xu, H., Chen, G.F., Ma, Y.S., Zhang, H.W., Zhou, Y., Liu, G.H., et al. (2020). Hepatic Proteomic Changes and Sirt1/AMPK Signaling Activation by Oxymatrine Treatment in Rats With Non-alcoholic Steatosis. *Frontiers in Pharmacology* 11. doi: 10.3389/fphar.2020.00216.

Xu, N., Luo, H., Li, M., Wu, J., Wu, X., Chen, L., et al. (2021). β-patchoulene improves lipid metabolism to alleviate non-alcoholic fatty liver disease via activating AMPK signaling pathway. *Biomed Pharmacother* 134**,** 111104. doi: 10.1016/j.biopha.2020.111104.

Xue, J.L., Liu, Y.C., Liu, B.Y., Jia, X.B., Fang, X.S., Qin, S.C., et al. (2024). <i>Celastrus orbiculatus</i> Thunb. extracts and celastrol alleviate NAFLD by preserving mitochondrial function through activating the FGF21/AMPK/PGC-1α pathway. *Frontiers in Pharmacology* 15. doi: 10.3389/fphar.2024.1444117.

Yan, L.S., Zhang, S.F., Luo, G., Cheng, B.C., Zhang, C., Wang, Y.W., et al. (2022). Schisandrin B mitigates hepatic steatosis and promotes fatty acid oxidation by inducing autophagy through AMPK/mTOR signaling pathway. *Metabolism* 131**,** 155200. doi: 10.1016/j.metabol.2022.155200.

Yang, Y., Qiu, W., Xiao, J.Y., Sun, J., Ren, X., and Jiang, L.X. (2024). Dihydromyricetin ameliorates hepatic steatosis and insulin resistance via AMPK/PGC-1α and PPARα-mediated autophagy pathway. *Journal of Translational Medicine* 22(1). doi: 10.1186/s12967-024-05060-7.

Yang, Y., Wu, Y., Zou, J., Wang, Y.H., Xu, M.X., Huang, W., et al. (2021). Naringenin Attenuates Non-Alcoholic Fatty Liver Disease by Enhancing Energy Expenditure and Regulating Autophagy via AMPK. *Frontiers in Pharmacology* 12. doi: 10.3389/fphar.2021.687095.

Yi, M., Fasina, O.B., Li, Y., Xiang, L., and Qi, J. (2023). Mixture of Peanut Skin Extract, Geniposide, and Isoquercitrin Improves the Hepatic Lipid Accumulation of Mice via Modification of Gut Microbiota Homeostasis and the TLR4 and AMPK Signaling Pathways. *Int J Mol Sci* 24(23). doi: 10.3390/ijms242316684.

Yin, X., Liu, Z., and Wang, J. (2023). Tetrahydropalmatine ameliorates hepatic steatosis in nonalcoholic fatty liver disease by switching lipid metabolism via AMPK-SREBP-1c-Sirt1 signaling axis. *Phytomedicine* 119**,** 155005. doi: 10.1016/j.phymed.2023.155005.

Yong, Z., Zibao, H., Zhi, Z., Ning, M., Ruiqi, W., Mimi, C., et al. (2022). Nootkatone, a Sesquiterpene Ketone From <i>Alpiniae oxyphyllae</i> Fructus, Ameliorates Metabolic-Associated Fatty Liver by Regulating AMPK and MAPK Signaling. *Frontiers in Pharmacology* 13. doi: 10.3389/fphar.2022.909280.

Yu, L.Y., Gong, L.H., Wang, C., Hu, N.H., Tang, Y.Q., Zheng, L., et al. (2020). Radix Polygoni Multiflori and Its Main Component Emodin Attenuate Non-Alcoholic Fatty Liver Disease in Zebrafish by Regulation of AMPK Signaling Pathway. *Drug Design Development and Therapy* 14**,** 1493-1506. doi: 10.2147/dddt.S243893.

Yuan, Z.Y., Lu, X., Lei, F., Sun, H., Jiang, J.F., Xing, D.M., et al. (2023). Novel Effect of <i>p</i>-Coumaric Acid on Hepatic Lipolysis: Inhibition of Hepatic Lipid-Droplets. *Molecules* 28(12). doi: 10.3390/molecules28124641.

Zeng, X.L., Yang, J.N., Hu, O., Huang, J., Ran, L., Chen, M.T., et al. (2019). Dihydromyricetin Ameliorates Nonalcoholic Fatty Liver Disease by Improving Mitochondrial Respiratory Capacity and Redox Homeostasis Through Modulation of SIRT3 Signaling. *Antioxidants & Redox Signaling* 30(2)**,** 163-183. doi: 10.1089/ars.2017.7172.

Zhang, C., Tong, Q., Liu, K., Mao, T., Song, Y., Qu, Y., et al. (2024). Morroniside delays the progression of non-alcoholic steatohepatitis by promoting AMPK-mediated lipophagy. *Phytomedicine* 129**,** 155703. doi: 10.1016/j.phymed.2024.155703.

Zhang, D., Zhang, Y.H., Wang, Z.L., and Lei, L. (2023a). Thymoquinone attenuates hepatic lipid accumulation by inducing autophagy via AMPK/mTOR/ULK1-dependent pathway in nonalcoholic fatty liver disease. *Phytotherapy Research* 37(3)**,** 781-797. doi: 10.1002/ptr.7662.

Zhang, J., Zhang, W., Yang, L., Zhao, W., Liu, Z., Wang, E., et al. (2023b). Phytochemical gallic acid alleviates nonalcoholic fatty liver disease via AMPK-ACC-PPARa axis through dual regulation of lipid metabolism and mitochondrial function. *Phytomedicine* 109**,** 154589. doi: 10.1016/j.phymed.2022.154589.

Zhang, J.J., Ma, X.X., and Fan, D.D. (2022). Ginsenoside CK ameliorates hepatic lipid accumulation <i>via</i> activating the LKB1/AMPK pathway <i>in vitro</i> and <i>in vivo</i>. *Food & Function* 13(3)**,** 1153-1167. doi: 10.1039/d1fo03026d.

Zhang, J.X., Yuan, Y.L., Gao, X.Y., Li, H., Yuan, F.X., Wu, D., et al. (2025). Scopoletin ameliorates hyperlipidemia and hepatic steatosis via AMPK, Nrf2/HO-1 and NF-κB signaling pathways. *BIOCHEMICAL PHARMACOLOGY* 231. doi: 10.1016/j.bcp.2024.116639.

Zhang, L., Li, H.X., Pan, W.S., Khan, F.U., Qian, C., Qi-Li, F.R., et al. (2019a). Novel hepatoprotective role of Leonurine hydrochloride against experimental non-alcoholic steatohepatitis mediated via AMPK/SREBP1 signaling pathway. *Biomed Pharmacother* 110**,** 571-581. doi: 10.1016/j.biopha.2018.12.003.

Zhang, Y., Wang, R.Q., Yao, H.J., Ma, N., Jiang, C.Z., Zhou, Y., et al. (2021). Mangiferin Ameliorates HFD-Induced NAFLD through Regulation of the AMPK and NLRP3 Inflammasome Signal Pathways. *Journal of Immunology Research* 2021. doi: 10.1155/2021/4084566.

Zhang, Y.P., Deng, Y.J., Tang, K.R., Chen, R.S., Liang, S., Liang, Y.J., et al. (2019b). Berberine Ameliorates High-Fat Diet-Induced Non-Alcoholic Fatty Liver Disease in Rats via Activation of SIRT3/AMPK/ACC Pathway. *CURRENT MEDICAL SCIENCE* 39(1)**,** 37-43. doi: 10.1007/s11596-019-1997-3.

Zhao, G., Yang, L., Zhong, W., Hu, Y., Tan, Y., Ren, Z., et al. (2022). Polydatin, A Glycoside of Resveratrol, Is Better Than Resveratrol in Alleviating Non-alcoholic Fatty Liver Disease in Mice Fed a High-Fructose Diet. *Front Nutr* 9**,** 857879. doi: 10.3389/fnut.2022.857879.

Zheng, X., Zhang, X.G., Liu, Y., Zhu, L.P., Liang, X.S., Jiang, H., et al. (2021). Arjunolic acid from<i> Cyclocarya</i><i> paliurus</i> ameliorates nonalcoholic fatty liver disease in mice via activating Sirt1/AMPK, triggering autophagy and improving gut barrier function. *Journal of Functional Foods* 86. doi: 10.1016/j.jff.2021.104686.

Zhong, Y., Li, Z., Jin, R., Yao, Y., He, S., Lei, M., et al. (2022). Diosgenin Ameliorated Type II Diabetes-Associated Nonalcoholic Fatty Liver Disease through Inhibiting De Novo Lipogenesis and Improving Fatty Acid Oxidation and Mitochondrial Function in Rats. *Nutrients* 14(23). doi: 10.3390/nu14234994.

Zhou, F., Ding, M.N., Gu, Y.Q., Fan, G.F., Liu, C.Y., Li, Y.J., et al. (2022). Aurantio-Obtusin Attenuates Non-Alcoholic Fatty Liver Disease Through AMPK-Mediated Autophagy and Fatty Acid Oxidation Pathways. *Frontiers in Pharmacology* 12. doi: 10.3389/fphar.2021.826628.

Zhu, J., Guo, J.N., Liu, Z.J., Liu, J., Yuan, A.N., Chen, H., et al. (2024). Salvianolic acid A attenuates non-alcoholic fatty liver disease by regulating the AMPK-IGFBP1 pathway. *Chemico-Biological Interactions* 400. doi: 10.1016/j.cbi.2024.111162.

Zhu, X.P., Bian, H., Wang, L., Sun, X.Y., Xu, X., Yan, H.M., et al. (2019). Berberine attenuates nonalcoholic hepatic steatosis through the AMPK-SREBP-1c-SCD1 pathway. *FREE RADICAL BIOLOGY AND MEDICINE* 141**,** 192-204. doi: 10.1016/j.freeradbiomed.2019.06.019.
